# Revealing the Impact of Aging on Perovskite Solar Cells Employing Nickel Phthalocyanine‐Based Hole Transporting Material

**DOI:** 10.1002/advs.202405284

**Published:** 2024-09-16

**Authors:** Muhammad Ans, Zekeriya Biyiklioglu, Apurba Mahapatra, Rohit D. Chavan, Joanna Kruszyńska, Muhittin Unal, Hilal Fazlı, Kostiantyn Nikiforow, Pankaj Yadav, Seckin Akin, Emre Güzel, Daniel Prochowicz

**Affiliations:** ^1^ Institute of Physical Chemistry Polish Academy of Sciences Kasprzaka 44/52 Warsaw 01–224 Poland; ^2^ Department of Chemistry Faculty of Science Karadeniz Technical University Trabzon 61080 Türkiye; ^3^ Laboratory of Advanced Materials & Photovoltaics (LAMPs) Necmettin Erbakan University Konya 42090 Türkiye; ^4^ Department of Solar Energy School of Energy Technology Pandit Deendayal Energy University Gandhinagar Gujarat 382 007 India; ^5^ Department of Physics School of Energy Technology Pandit Deendayal Energy University Gandhinagar Gujarat 382 007 India; ^6^ Department of Metallurgical and Materials Engineering Necmettin Erbakan University Konya 42090 Türkiye; ^7^ Department of Engineering Fundamental Sciences Faculty of Technology Sakarya University of Applied Sciences Sakarya 54050 Türkiye

**Keywords:** aging stability, efficiency, hole transporting material, metal phthalocyanine, perovskite solar cells

## Abstract

The enhancement of the photovoltaic performance upon the aging process at particular environment is often observed in perovskite solar cells (PSCs), particularly for the devices with 2,2′,7,7′‐tetrakis(N,N‐di(4‐methoxyphenyl)amino)−9,9′‐spirobifluorene (spiro‐OMeTAD) as hole transporting material (HTM). In this work, for the first time the effect of aging the typical n‐i‐p PSCs employing nickel phthalocyanine (coded as Bis‐PF‐Ni) solely as dopant‐free HTM is investigated and as an additive in spiro‐OMeTAD solution. This study reveals that the prolong aging of these devices at dry air condition (RH = 2%, 25 °C) is beneficial for the improvement of their performances. Various bulk and surface characterization techniques are utilized to understand the factors behind the spontaneous efficiency enhancement of the devices after storage. As a result, the changes in properties of the Bis‐PF‐Ni layer are observed and at perovskite/Bis‐PF‐Ni interface, which ultimately improves the charge transport and reduces non‐radiative recombination. In addition, the devices with Bis‐PF‐Ni HTM reveal enhanced long‐term ambient and thermal stability compared to the PSCs based on doped spiro‐OMeTAD.

## Graphical abstract

This study reveals the effect of aged Bis‐PF‐Ni HTM on the overall performance of PSCs. The observed self‐enhancement of the PCE is attributed to the enhanced conductivity, better band alignment of Bis‐PF‐Ni, reduces trap‐assisted recombination, and improves charge transport. The devices demonstrated superior ambient and thermal stability.

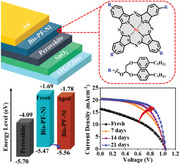

## 1 Introduction

Perovskite solar cells (PSCs) have emerged as a competitive alternative to conventional silicon‐based solar cells owing to the ease of fabrication processes, low cost and high power conversion efficiency (PCE) exceeding 26%.^[^ ^1^, ^2^ ^]^ Despite this remarkable PCE, bringing PSCs closer to commercialization for outdoor applications is only possible by achieving long‐term ambient and operational stability.^[^ ^3^, ^4^, ^5^ ^]^ Over the last couple of years, researchers have witnessed significant developments in tackling the stability issues by compositional engineering of perovskite absorbers and interfacial engineering of electron and hole transporting layers (ETLs and HTLs).^[^ ^6^, ^7^, ^8^ ^]^ The latter plays an essential role in extracting and collecting the photogenerated charges from the perovskite absorber to the respective electrode, thereby circumventing undesired recombination losses at the interfaces and improving the photovoltaic performance and stability of the device.^[^ ^9^, ^10^, ^11^, ^12^ ^]^ So far, the state‐of‐the‐art and highly efficient PSCs commonly used spiro‐OMeTAD as hole transporting materials (HTMs).^[^ ^13^, ^14^, ^15^, ^16^ ^]^ However, spiro‐OMeTAD HTM suffers not only from the expensive and tedious synthetic routes but also from low stability under thermal stress.^[^ ^17^, ^18^, ^19^ ^]^ Furthermore, the hygroscopic Bis(trifluoromethane)sulfonimide lithium salt (LiTFSI) and volatile 4‐*tert*‐butyloyridine (t‐BP) dopants required to prepare HTL solution play a negative impact on the long‐term durability of PSCs.^[^ ^20^, ^21^ ^]^ As a result, there is significant research interest in seeking alternative HTMs that can address the limitation of spiro‐OMeTAD.^[^ ^22^, ^23^ ^]^

Recently, metal phthalocyanines (MPcs) have received a great attention as potential HTMs due to their low‐cost synthesis, high carrier mobility and high thermal stability.^[^ ^24^ ^]^ By optimizing the type of metal ions and peripheral substituents on the Pc ring, the chemical and physical characteristics of MPcs could be well‐adjusted.^[^ ^25^, ^26^ ^]^ For instance, a series of MPcs was synthesized by modifying the peripheral substituents to improve the molecular arrangement in thin films and, consequently, charge transport properties.^[^ ^27^, ^28^, ^29^ ^]^ In turn, enhanced solubility and hydrophobicity of MPcs could be achieved by adding side‐chain groups to their periphery.^[^ ^30^ ^]^ Furthermore, these alkyl chains could contribute to achieving better morphology of MPc films when deposited on the perovskite, favorable π‐π interactions, and higher charge carrier mobility.^[^ ^31^, ^32^, ^33^ ^]^ Recent works demonstrated that the n‐i‐p devices with various MPcs as dopant‐free HTM can reach efficiency >17% (with the current certified PCE record of 21.03%) by adjusting the peripheral substituents in MPcs.^[^ ^27^, ^28^, ^34^, ^35^, ^36^ ^]^ However, these works lack information on whether the highest PCE was collected for the as‐fabricated or aged devices. In the work of Zhang et al., it was mentioned that the highest efficiency of their devices based on Zn(II) Pc HTM was achieved after several days of preparation.^[^ ^37^ ^]^ However, this aspect has not been studied in detail by the authors. The self‐enhancement of PCE during the aging process at particular environment was previously observed for the devices with spiro‐OMeTAD HTM.^[^ ^38^ ^]^ It was suggested that the spontaneous PCE improvement of the device after storage could result from the changes in the perovskite surface morphology (coalescence of perovskite crystals)^[^ ^39^, ^40^ ^]^ or spiro‐OMeTAD HTL properties, i.e., improved conductivity and reduced HOMO level.^[^ ^41^, ^42^, ^43^ ^]^ Recently, Cho et al. reported that the reduction of surface recombination in aged devices could explain the initial PCE improvement for stored devices.^[^ ^44^ ^]^ To our best knowledge, there is no study that explains the effect of aging devices with MPc HTM on spontaneous PCE improvement.

Herein, we study the factors contributing to the origin of initial PCE increase by the aging of n‐i‐p devices with nickel phthalocyanine (Bis‐PF‐Ni) as dopant‐free HTM. We found that PCE significantly increases with increasing aging time reaching 13.94% (from initial 7.04%) after 21 days of storage at dry air condition (RH = 2%, 25 °C). The effect of aging time on the changes in Bis‐PF‐Ni layer, perovskite/Bis‐PF‐Ni interface and the completed device was experimentally investigated using various methods including scanning electron microscopy (SEM), atomic force microscopy (AFM), X‐ray diffraction analysis (XRD), time‐resolved photoluminescence (TRPL), electrical impedance spectroscopy (EIS), optical polarizing microscopy (POM), and ultraviolet photoelectron spectroscopy (UPS). As a result, we proposed that the observed self‐improvement of PCE after aging is a combined effect of gradual changes in the electrical properties of the Bis‐PF‐Ni layer and the quality of the perovskite/Bis‐PF‐Ni interface, which improves the charge transport and reduces nonradiative recombination. In addition, an impressive self‐enhancement in the PCE from 13.78% to 19.70% was observed using Bis‐PF‐Ni as an additive in spiro‐OMeTAD solution. Finally, we demonstrate that the devices with Bis‐PF‐Ni HTM show promising long‐term stability under various conditions compared with the PSCs based on benchmark doped spiro‐OMeTAD HTM.

## 2 Results and Discussion

### 2.1 Synthesis and Characterization of the Bis‐PF‐Ni

**Figure 1:**
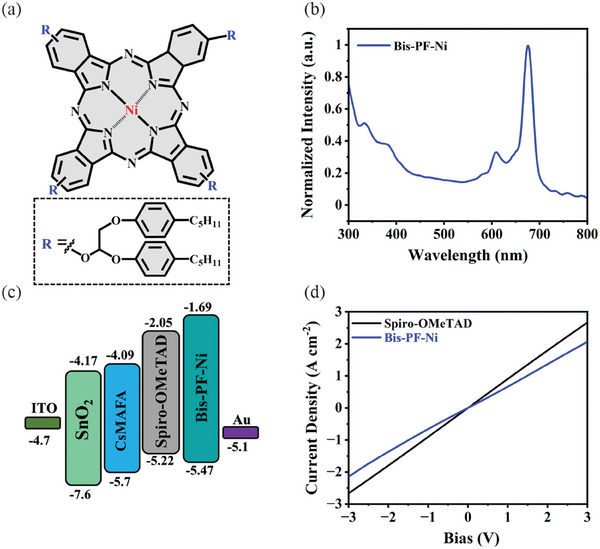
a) Molecular structure of the Bis‐PF‐Ni. b) UV–vis spectrum of the Bis‐PF‐Ni in chlorobenzene. c) Schematic energy band diagram of the device layers. The energy levels for perovskite and spiro‐OMeTAD are taken from the literature. d) Current‐voltage (*J‐V*) characteristics of the Bis‐PF‐Ni, and doped spiro‐OMeTAD films deposited on ITO glass.

The newly prepared nickel phthalocyanine derivative, namely Bis‐PF‐Ni, was molecularly engineered by the introduction of tetra‐[2,3‐bis(4‐pentylphenoxy)propoxy] substituents in the periphery of the Pc ring to improve the solubility and increase hydrophobicity (**Figure** **1**a). Bis‐PF‐Ni was synthesized by cyclotetramerization of the 4‐[2,3‐bis(4‐pentylphenoxy)propoxy]phthalonitrile with the presence of metal salt (NiCl_2_) under nitrogen atmosphere (for synthetic details see Experimental Section). Hereby, Bis‐PF‐Ni is isolated and purified with reasonable yield (31%). More detailed information regarding the NMR and mass spectroscopy characterizations of starting compounds and Bis‐PF‐Ni can be found in the Supporting Information (Figures S1–S13, Supporting Information). We also studied the thermal stability and mesogenic property of Bis‐PF‐Ni using thermogravimetric analyzer (TGA) and optical polarizing microscopy (POM), respectively. As shown in Figure S14 (Supporting Information), Bis‐PF‐Ni is relatively stable and starts degrading at over 200 °C. Figure S15 (Supporting Information) shows the textures of liquid crystalline phases observed under POM with crossed polarizers. Bis‐PF‐Ni shows dendritic and fan‐like textures formed during annealing of the samples above 40 °C, which is characteristic of the columnar hexagonal ordering of the mesogen. The X‐ray diffraction data of the Bis‐PF‐Ni is shown in Figure S16 (Supporting Information) and summarized in Table S1 (Supporting Information). The XRD pattern of Bis‐Ni‐Pc corresponds to the columnar hexagonal packing of the mesogens. Bragg reflections at small angles have typical ratios of the *d* values of 1:√3:√7:√9, which are indicative for a 2D hexagonal lattice with the unit cells ahex 27.20 (Table S1, Supporting Information). There is also an additional reflection at 3.41 Å, which can be attributed to the packing of macrocycles in columns. The diffuse halo in this region indicates the absence of long‐range order and disordering of molecules along the stack axis, and, as a consequence, the formation of a discotic hexagonal disordered mesophase (Col_h_).^[^ ^45^, ^46^, ^47^ ^]^ Based on this study, the incorporation of alkyl units as side groups in Bis‐PF‐Ni gave the phthalocyanine structure highly condensed and ordered phases of liquid crystalline characteristics by molecular alignment into columnar stacks that can improve HTL homogeneity and enhance conductivity.

Next, we employed density functional theory (DFT) calculations to study the electronic properties of Bis‐PF‐Ni. Figure S17 (Supporting Information) shows the optimized molecular structure and the frontier molecular orbitals (FMOs). The HOMO electron density distribution is mainly delocalized on the Pc ring without side chains and metal central ions. Conversely, the LUMO level is largely localized on the Pc ring as well as on the Ni metal ion, which might indicate the contribution of Ni ion toward charge transfer. The optical and electrochemical properties of Bis‐PF‐Ni were experimentally characterized by UV–vis absorption spectroscopy and cyclic voltammetry (CV). The UV–vis spectrum of Bis‐PF‐Ni solution in chlorobenzene is depicted in Figure 1b. Bis‐PF‐Ni exhibits two typical characteristic strong absorption bands of Bis‐PF‐Ni. Here, the B band that occurs because of deeper π–π* transitions is located in the ultraviolet region between 300 and 350 nm, and the Q band can be found in the region between 580 and 680 nm because of π–π* transitions. In addition, the Q band appeared as doublets (610 and 676 nm), which could be interpreted due to Q‐band vibration as π–π* excitation between bonding and antibonding molecular orbitals.^[^ ^48^ ^]^ To investigate the potential application of Bis‐PF‐Ni as HTM in PSCs, its electronic feature was measured by CV in 1,2‐dichlorobenzene solution. The HOMO level of the Bis‐PF‐Ni was estimated from the onset of the oxidation waves, referred to the Ag/AgCl oxidation couple, and was found to be −5.47 eV (Figure S18a, Supporting Information). The LUMO level was calculated by subtracting the optical band gap from the HOMO value and found to be −1.69 eV (Figure S18b, Supporting Information). For comparison, the DFT estimation of the Bis‐PF‐Ni HOMO level was found to be −5.36 eV (Figure S17, Supporting Information), which aligns with the experimental CV results. As shown in Figure 1c, the HOMO level of Bis‐PF‐Ni is energetically favorable for hole extraction from perovskite compared to the HOMO level of spiro‐OMeTAD. In addition, Bis‐PF‐Ni has a much higher lying LUMO level than that of spiro‐OMeTAD, which can enhance electron blocking from perovskite to the Au electrode.^[^ ^49^ ^]^ Another important parameter to ensure effective transport of the photogenerated holes from the perovskite to the metal electrode is hole conductivity of HTM. Figure 1d shows the current–voltage (*I–V*) relations of ITO/HTM/Au films, from which slope conductivity can be calculated. The conductivity of Bis‐PF‐Ni is estimated to be 1.38 × 10^−6^ S cm^−1^, which is higher than that of undoped spiro‐OMeTAD film (10^−7^–10^−8^ S cm^−1^).^[^ ^50^, ^51^ ^]^ For comparison, the conductivity of doped spiro‐OMeTAD was also measured under the same conditions reaching 1.79 × 10^−5^ S cm^−1^. The higher conductivity of spiro‐OMeTAD is related to the presence of dopants. In addition, the hole mobility of the Bis‐PF‐Ni determined by using the space charge limited current (SCLC) method with a device structure of ITO/PEDOT:PSS/HTM/Au was found to be 1.34 × 10^−6^ cm^2^ V^−1^ s^−1^, which is in good agreement with the literature (Figure S19, Supporting Information).^[^ ^52^ ^]^

### 2.2 Surface and Interface Properties of Bis‐PF‐Ni with the Perovskite Layer

**Figure 2:**
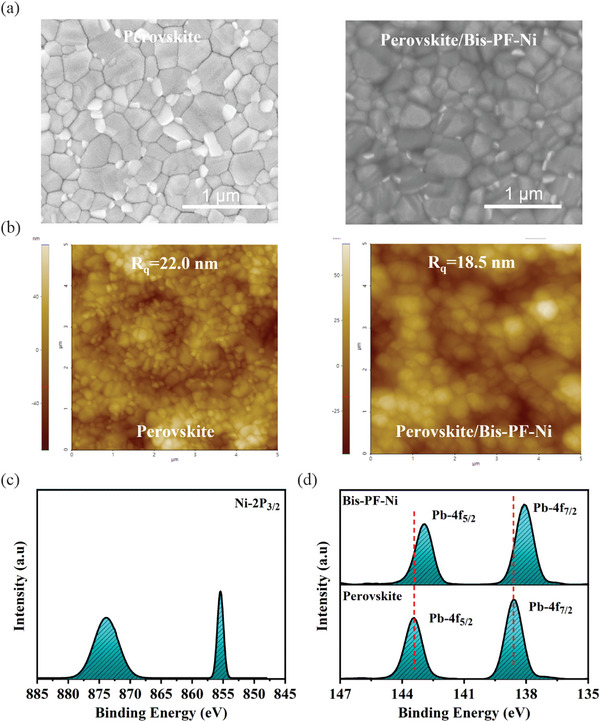
a) Top view SEM images of the pristine perovskite (left) and perovskite/Bis‐PF‐Ni (right) films. b) AFM images of the pristine perovskite (left) and perovskite/Bis‐PF‐Ni (right) films. c) Ni 2p_3/2_ XPS spectrum from the perovskite/Bis‐PF‐Ni film. d) Pb 4f XPS spectrum from the pristine perovskite and perovskite/Bis‐PF‐Ni films.

The uniform coverage of the perovskite surface with the HTM film and its low surface roughness is significant importance for high‐performance PSCs. The film‐forming ability of Bis‐PF‐Ni (using optimized concentration of 5 mg mL^−1^) was investigated with the aid of scanning electron microscopy (SEM) and atomic force microscopy (AFM) techniques. As shown in **Figure** **2**a, the perovskite layer was evenly coated with the Bis‐PF‐Ni film. Based on AFM measurements, the root‐mean‐square (RMS) roughness of the perovskite surface decreased from 22.0 nm to 18.5 nm (Figure 2b). Note that there is no significant difference in the grain size of the perovskite film after the deposition of Bis‐PF‐Ni film. Considering the presence of heteroatoms in the Bis‐PF‐Ni, we further probed the molecular interaction between perovskite and Bis‐PF‐Ni by X‐ray photoelectron spectroscopy (XPS) measurement. Figure 2c shows the Ni 2p_3/2_ XPS peaks at 855.5 eV and 873.8 eV, which confirms the presence of Ni^2+^ on the surface of perovskite film. In the Pb 4f XPS spectrum, the peaks at 143.44 and 138.61 eV assigned to the Pb 4f_5/2_ and Pb 4f_7/2_ of the pristine perovskite film shift toward lower binding energies (142.89 and 138.0 eV) after depositing Bis‐PF‐Ni (Figure 2d). This peak shift can be assigned to coordinative interaction between the perovskite and Bis‐PF‐Ni layer, which is further investigated with the aid of DFT. It is expected that the lone pair electrons on O and N atoms in Bis‐PF‐Ni molecule could act as Lewis bases and interact with the uncoordinated Pb^2+^ sites, offering enhanced photovoltaic parameters.^[^ ^53^ ^]^ To illustrate the binding sites (nucleophilic and electrophilic regions) towards perovskite, electrostatic potential (ESP) analysis was conducted. The analysis demonstrates that the O and N atoms exhibit a significant electron density, indicating their robust electron‐donating capability, as depicted in Figure S20 (Supporting Information). Thus, Bis‐PF‐Ni has the ability to create strong connection with the perovskite surface, which should result in the effective sealing of imperfections and improved durability.

### 2.3 Fabrication, Characterization and Aging of PSCs with Bis‐PF‐Ni as HTM

Encouraged by promising electrical properties, Bis‐PF‐Ni was tested as HTM in a standard n‐i‐p PSC with the architecture of ITO/SnO_2_/perovskite/HTL/Au (for the device fabrication details, see the Experimental section). The triple cation perovskite composition of [Cs_0.05_(FA_0.95_MA_0.05_)_0.95_Pb(I_0.95_Br_0.05_)_3_] was prepared according to our previous method.^[^ ^54^ ^]^ A solution of Bis‐PF‐Ni in chlorobenzene was spin‐coated on the top of the perovskite layer with various concentrations. A concentration of 5 mg mL^−1^ was found to be the optimal condition in terms of champion efficiency (Figure S21, Supporting Information). The statistic photovoltaic parameter distributions, are illustrated in Figure S22 and Table S2, Supporting Information). The cross‐sectional SEM image of the investigated device shows a well‐ordered stacked structure (Figure S23, Supporting Information). The thickness of the spin‐coated Bis‐PF‐Ni film measured using a reflectometer is about 15 nm.

**Figure 3:**
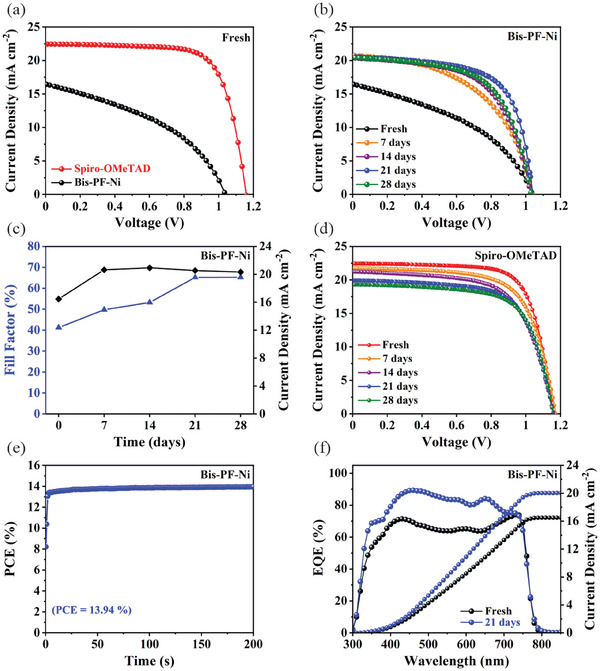
a) *J–V* curves of the best performing Bis‐PF‐Ni and spiro‐OMeTAD (as control device) based PSCs under reverse scan. b) *J–V* curves of the device based on Bis‐PF‐Ni HTM at different aging times. c) Evolution of FF and *Jsc* of the Bis‐PF‐Ni based device over time. d) *J–V* curves of the device based on spiro‐OMeTAD HTM at different aging times. e) Steady‐state PCE of the Bis‐PF‐Ni based device. f) External quantum efficiency and integrated *J_SC_*_._

The current density–voltage (*J–V*) characteristics for the best performing Bis‐PF‐Ni and spiro‐OMeTAD (as control device) based PSCs are shown in **Figure** **3**a and photovoltaic parameters are summarized in **Table** **1** and Table S3 (Supporting Information). It can be seen that the device based on Bis‐PF‐Ni HTM reaches a maximum PCE of 7.04% under the AM 1.5 G irradiation at 100 mW cm^−2^ with an open‐circuit voltage (*V_OC_*) of 1.03 V, a short‐circuit photocurrent (*J_SC_*) of 16.46 mA cm^−2^ and a fill factor (*FF*) of 41.23%. This performance was much lower than that of the best spiro‐OMeTAD based device with a PCE of 18.82%. The electrochemical impedance spectroscopy (EIS) was used under dark conditions to elucidate the reason of low performance in Bis‐PF‐Ni based devices and get more information on the effect of perovskite/HTL interface and recombination kinetics on the charge transport properties in the studied devices. The fitted Nyquist plots of the Bis‐PF‐Ni and spiro‐OMeTAD based devices are shown in Figure s24 and all the fitted parameters are shown in Table S4 (Supporting Information). The series resistance (R_S_) is mainly correlated to the ITO/ETL interface contact and the observed values of R_S_ of both devices suggest a similar interface quality. In turn, the feature at low frequencies has been mainly correlated with the recombination resistance (R_REC_) and ionic diffusion, while that at the high frequencies could be attributed to the contact resistance (*R_SH_*) related to the charge transfer process under dark conditions.^[^ ^55^ ^]^ Therefore, R_SH_ mainly represents the ETL/perovskite and perovskite/HTL interface properties by giving information about the charge transport in PSCs. The high R_SH_ of the device based on Bis‐PF‐Ni HTM indicates poor charge transport at the perovskite/HTL interface, which could be due to the high roughness and low conductivity. In addition, the value of R_REC_ of the device based on Bis‐PF‐Ni HTM is almost 5 times lower than that of the control device, which suggests a high level of charge recombination.

**Table 1:** Photovoltaic parameters of the Bis‐PF‐Ni and spiro‐OMeTAD based devices under different aging times at dry air chamber (RH = 2%, 25 °C) in the backward direction.

HTL	Aging time		V_OC_ [V]	J_SC_ [mA cm^−2^]	FF [%]	PCE [%]
Bis‐PF‐Ni	Fresh	Champion	1.032	16.46	41.23	7.04
		Average	1.021 ± 0.01	16.67 ± 0.21	40.07 ± 0.71	6.87 ± 0.12
	7 days	Champion	1.045	20.90	50.85	11.07
		Average	1.040 ± 0.01	20.77 ± 0.18	50.3 ± 0.21	10.96 ± 0.05
	14 days	Champion	1.030	20.4	59.18	12.41
		Average	1.030 ± 0.01	20.20 ± 0.15	58.29 ± 0.34	12.11 ± 0.11
	21 days	Champion	1.042	20.54	65.21	13.94
		Average	1.046 ± 0.01	20.52 ± 0.14	63.84 ± 1.30	13.74 ± 0.31
	28 days	Champion	1.032	20.45	60.58	12.77
		Average	1.031 ± 0.01	20.22 ± 0.15	59.06 ± 0.86	12.30 ± 0.41
Spiro‐OMeTAD	Fresh	Champion	1.15	22.45	72.37	18.82
		Average	1.145 ± 0.01	22.43 ± 0.03	71.175 ± 1.01	18.34 ± 0.30
	7 days	Champion	1.174	21.81	69.96	18.01
		Average	1.173 ± 0.01	21.78 ± 0.11	68.40 ± 1.02	17.54 ± 0.36
	14 days	Champion	1.164	21.45	64.50	16.14
		Average	1.152 ± 0.01	21.43 ± 0.23	63.26 ± 1.14	16.04 ± 0.45
	21 days	Champion	1.157	19.74	66.78	15.25
		Average	1.154 ± 0.01	19.67 ± 0.20	64.48 ± 2.35	14.64 ± 0.54
	28 days	Champion	1.158	19.31	67.73	15.14
		Average	1.155 ± 0.01	19.24 ± 0.18	65.35 ± 2.31	14.54 ± 0.62

We note that these performances were collected after 24 h of aging devices at dry air chamber (RH = 2%, 25 °C) as required for full oxidation of spiro‐OMeTAD.^[^ ^43^ ^]^ Interestingly, we observed the systematic enhancement of the PCE for the device with Bis‐PF‐Ni with increasing the aging time (Figure 3b and Table 1). After 21 days, the device reaches a maximum PCE of 13.94% with a V_OC_ of 1.04 V, J_SC_ of 20.54 mA cm^−2^ and FF of 65.21% (for statistic photovoltaic parameter distributions, see Figure S25, Supporting Information). Further aging led to a drop in PCE to 13.24% due to a drop in J_SC_ and FF (Figure 3c). In contrast, the PCE of the control device based on spiro‐OMeTAD continuously dropped with aging time and reached a PCE of 15.14% after 28 days (Figure 3d and Table 1). As previously reported, this decrement in PCE could be attributed to the prolonged oxidation of the spiro‐OMeTAD, which accelerates the degradation of the perovskite crystals.^[^ ^17^ ^]^ To verify the role of a dry oxygen atmosphere, we made an experiment by taking a freshly prepared Bis‐PF‐Ni device and stored under an inert atmosphere for a certain period. It is found that no enhancement in PCE is observed (Figure S26, Supporting Information). This confirms the beneficial role of making aging process under a dry oxygen atmosphere. The value of PCE for Bis‐PF‐Ni based PSC was confirmed by measuring the device under maximum power point (MPP) tracking with continuous illumination for 200 s, reaching a stabilized value of 13.94% (Figure 3e). To confirm the increase in current with increasing the aging time, the EQE studies were performed on the fresh (1 day aged) and aged device (after 21 days) as shown in Figure 3f. As seen, the EQE of the aged device shows an improvement over the whole absorption wavelength range. Simultaneously, the values of integrated J_SC_ obtained from the EQE spectra increased for the aged device. In contrast, the EQE of the aged spiro‐OMeTAD based device decreased with the aging time (Figure S27, Supporting Information).

### 2.4 Elucidating the Factors for Self‐Enhanced PCE After the Aging Process

**Figure 4:**
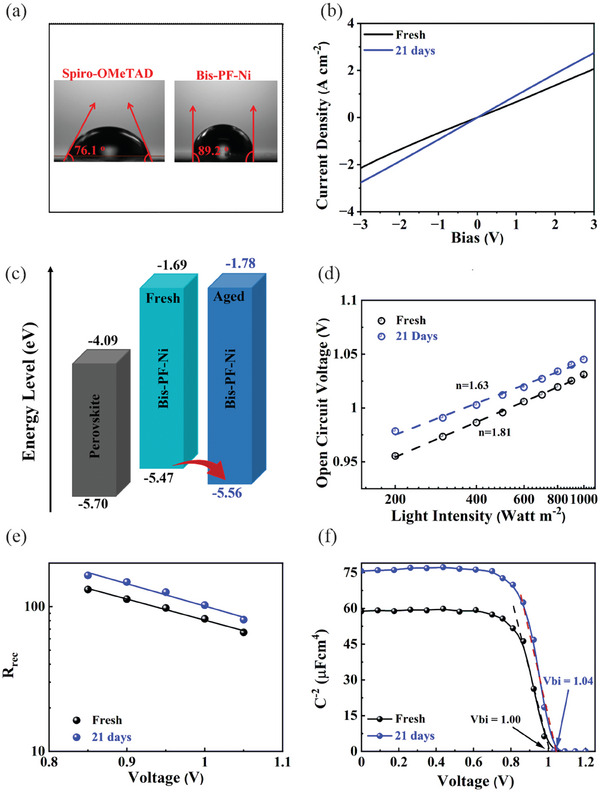
a) Water contact angles on various perovskite/HTL films. b) Conductivity and c) energy levels of Bis‐PF‐Ni film at different aging times. d) Light intensity dependence of V_OC_ under reverse scanning. e) Recombination resistance (R_rec_) derived from the high‐frequency region as a function of applied voltage. f) Mott–Schottky plots at 10 kHz in the Bis‐PF‐Ni based devices at different aging times.

Recently, Roose et. al. demonstrated that the small crystals of the perovskite film can spontaneously coalesce to form larger crystals after storing them in the dark at room temperature.^[^ ^40^ ^]^ This process leads to a decrease in the quantity of grain boundaries and trap states, which inhibits the non‐radiative recombination. To explore the factors of the self‐enhancement of the PCE, we first studied whether the crystals of the fresh perovskite film can coalesce into larger crystals in the presence of Bis‐PF‐Ni layer upon aging time. The XRD patterns of the perovskite/Bis‐PF‐Ni films at different aging times are depicted in Figure S28 (Supporting Information). In general, the XRD peak intensity can be correlated with the degree of sample crystallinity. As seen, the XRD patterns of the aged perovskite/Bis‐PF‐Ni films confirm the retention of a perovskite crystal structure with no subtle change in the peak position and intensity. Furthermore, the SEM analysis of the fresh and aged films revealed no change in the morphology of the perovskite surface after aging (Figure S29, Supporting Information). Thus, it is expected that Bis‐PF‐Ni can efficiently shield the perovskite layer from moisture erosion and hinder coalescence of perovskite crystals. The hydrophobicity of the Bis‐PF‐Ni was investigated by measuring the water contact angle on the perovskite/Bis‐PF‐Ni film. As seen in **Figure** **4**a, the water contact angle of Bis‐PF‐Ni (89.2°) is higher than that of Spiro‐OMeTAD (76.1°), presumably due to the presence of long alkyl chain of substituents (tetra‐[2,3‐bis(4‐pentylphenoxy)propoxy]) attached to the Pc ring.

Considering the above results, we exclude the age‐induced recrystallization of perovskite film as a reason for improved device performance. Therefore, we evaluate the effect of aging process on the HTL by analysing the conductivity and HOMO level of Bis‐PF‐Ni film before and after aging. Figure 4b shows the *I‐V* relations of the fresh and aged ITO/Bis‐PF‐Ni/Au film. It is calculated from the slope of curves that the conductivity of Bis‐PF‐Ni HTM increases from 1.38 × 10^−6^ S cm^−1^ to 1.85 × 10^−6^ S cm^−1^ after aging for 21 days. High conductivity is expected to reduce the series resistance and improve the FF causing a higher PCE.^[^ ^56^ ^]^ In addition, aging of the Bis‐PF‐Ni film was found to alter the HOMO level as evidenced by ultraviolet photoelectron spectroscopy (UPS). As shown in Figure S30 (Supporting Information), the HOMO energy level obtained by the equation 21.22 − (E_cutoff_ − E_onset_) equal −5.47 eV and −5.56 eV for the fresh and aged Bis‐PF‐Ni film, respectively. Consequently, the aged film shows a more favourable energy level alignment with the valence band of perovskite material, which is beneficial for hole extraction from the perovskite layer into HTL (Figure 4c). The enhanced conductivity of the aged Bis‐PF‐Ni film and its better band alignment with the perovskite layer can explain the improvement of J_SC_ and FF of associated devices. To further explore the charge transport dynamics, time‐resolved photoluminescence (TRPL) characterizations were conducted on the perovskite/Bis‐PF‐Ni films (Figure S31, Supporting Information). We fitted the decay curves using the biexponential equation: (y = A_1_exp(−x/τ_1_) + A_2_exp(−x/τ_2_) + y_0_), where τ_1_ and τ_2_ refer to the fast and slow decay associated with surface and bulk recombination's, respectively.^[^ ^57^ ^]^ The corresponding fitted parameters are listed in Table S5 (Supporting Information). As seen, the fresh perovskite/Bis‐PF‐Ni film exhibited longer carrier lifetimes (τ_1_ = 9.98 ns, τ_2_ = 436.6 ns) than that of aged film (τ_1_ = 7.24 ns, τ_2_ = 425.5 ns). This further confirms that aging process enhanced ability of Bis‐PF‐Ni to extract charge carriers from perovskite. Next, a dark *J–V* measurement of the fresh and aged devices was performed to monitor the change in the trap states of the device upon aging (Figure S32a, Supporting Information).^[^ ^58^ ^]^ The dark ideality factors (*n*) of both the devices were extracted from the semi‐log *J–V* plots using the following equation $n=1/(\frac{q}{K_{B}T}\frac{\partial \ln J}{\partial V})$, where q, k_B_, and T are charge of the electron, Boltzmann constant, and temperature respectively (Figure S32b, Supporting Information). The negligible decrease in the value of *n* over time suggests minor effect of aging on the trap states of device.

To further understand the aging effect on the charge extraction and recombination, the illumination‐dependent photovoltaic parameters were collected. The change of *J_SC_* with the light illumination intensities is shown in Figure S32c (Supporting Information), and the slope value of *α* increases upon aging process. The high value of *α* indicates better charge extraction, which helps to increase the overall performance of the aged Bis‐PF‐Ni based devices.^[^ ^58^ ^]^ The dependence of V_OC_ on the incident light intensity is commonly used to express the charge recombination process and the ideality factors (*n*) under light can be obtained using the equation:$n=\frac{q}{K_{B}T}$ (∂ *V_OC_*/∂lnI), where I is intensity of incident light (Figure 4d). The ideality factor reduces from 1.81 to 1.63 for the aged device, which indicates the reduction of trap−assisted recombination after aging process.^[^ ^58^ ^]^ To shed more light on the origin of lower ideality factor for the aged device, EIS as a function of applied bias was recorded under AM 1.5 G light illumination. Figure S32d,e (Supporting Information) shows the bias‐dependent IS spectra of the fresh and aged device in the frequency range from 10^6^ to 0.1 Hz measured under AM 1.5 G light illumination. The Nyquist plots for both devices contain two semicircles at low‐ and high‐frequency regions with a small series resistance (R_S_). Under illumination, the semicircle in the high‐frequency area provides useful information on the charge carrier recombination process (R_HF_). It is observed that R_HF_ of the fresh device increases by ≈25% after 21 days aging time (Figure 4e). The increase values of R_HF_ indicate that the charge recombination at the interface is largely suppressed. Moreover, the lower R_S_ (Figure S32f, Supporting Information) of the aged device suggests that the barrier for the transport of photogenerated charge carriers is reduced, which could be the reason behind better charge extraction and higher values of V_OC_ and FF.^[^ ^59^ ^]^ Finally, the Mott‐Schottky test was performed to further confirm the improved charge carriers transfer property and estimate the built‐in potential (V_bi_) of the aged device (Figure 4f). The high value of V_bi_ of the aged device allows efficient charge extraction and reduces recombination compared to the fresh device. The improved charge extraction and lower recombination in the aged device can be due to the improved conductivity of the aged Bis‐PF‐Ni film and better interface formation between perovskite/HTL.

Next, we investigate whether the change on the surface of the perovskite/Bis‐PF‐Ni film could also explain the self‐enhancement of the PCE during aging process. Interestingly, the AFM results show that the aged perovskite/Bis‐PF‐Ni film presented a smoother surface morphology compared to the spiro‐OMeTAD film (Figures S33, S34, Supporting Information). For the reliability of our results, we considered three different spots during AFM image collection and monitored their change on time. As seen, the relative roughness of the perovskite/Bis‐PF‐Ni film significantly decreases with increasing aging time. In contrast, the surface roughness of the perovskite/spiro‐OMeTAD films notably increases with increasing aging time. A smooth surface of the perovskite/Bis‐PF‐Ni film is desirable to enable better contact between the HTL and metal electrode, and subsequently improve the photo‐generated charge transfer efficiency. It could be attributed to the possible change of the interfacial interactions between Bis‐PF‐Ni and perovskite as observed by XPS results. To confirm it, we monitor the change of Pb 4f XPS spectrum of the perovskite/Bis‐PF‐Ni film after aging process (Figure S35, Supporting Information). As seen, the Pb 4f peaks at 142.89 and 138.0 eV of the fresh (after 1 day) perovskite/Bis‐PF‐Ni film shift toward higher binding energies (143.11 and 138.25 eV) suggesting the decrease of interaction between perovskite and Bis‐PF‐Ni. In contrast, there is no significant change in the Ni 2p_3/2_ XPS spectrum of the aged perovskite/Bis‐PF‐Ni film and no signal related to the higher oxidized state of Ni (Figure S36, Supporting Information).

### 2.5 Bis‐PF‐Ni as an Additive in the Spiro‐OMeTAD HTL

**Figure 5:**
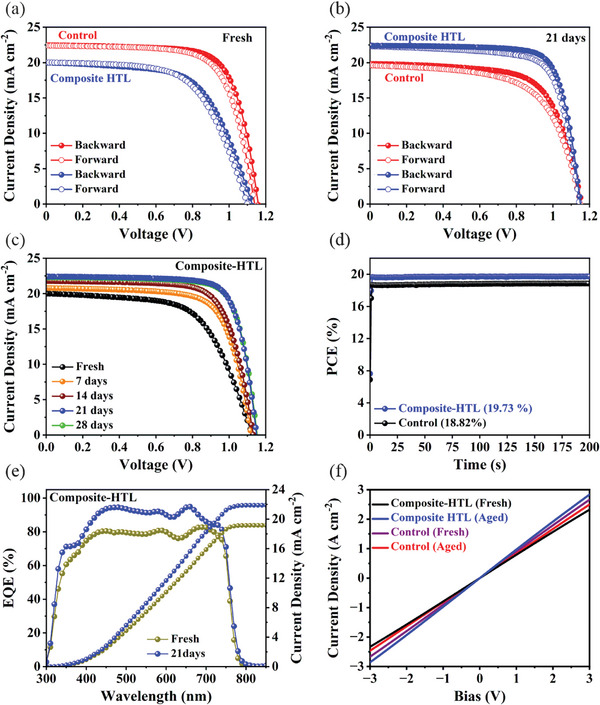
*J–V* curves of the control and composite HTL‐based devices after a) 1 day (fresh) and b) 21 days aging process. c) *J–V* curves at different aging time of the device based on composite HTL. d) Steady‐state PCE of the control and composite HTL‐based devices. e) External quantum efficiency and integrated *J_SC_*_._ f) Current‐voltage characteristics of the control and composite HTL based films deposited on ITO glass (fresh and aged).

Recent works revealed that mixing metal phthalocyanine derivatives with the spiro‐OMeTAD HTM offers better film uniformity and enhances the charge transport properties.^[^ ^49^, ^60^ ^]^ Here, we investigate whether a similar self‐enhancement of the PCE could be observed after the addition of Bis‐PF‐Ni into the spiro‐OMeTAD solution and aging of the resulting film. Initially, the 24 h aged device using Bis‐PF‐Ni with the previously optimized concentration of 5 mg mL^−1^ in the HTM layer of spiro‐OMeTAD showed a PCE of 13.78%, V_OC_ of 1.12 V, *J_SC_* of 19.99 mA cm^−2^ and FF of 61.25%, which was much lower in compared to the control device based on spiro‐OMeTAD HTL (**Figure** **5**a). When stored in a low dry chamber, the PCE of the device based on composite HTL (spiro‐OMeTAD + Bis‐PF‐Ni) increases with increasing the aging time as seen by the *J‐V* curves presented in Figure 5b,c. After a duration of 21 days, the device with the composite HTL exhibited a PCE of 19.70%, which surpasses the PCE of the freshly prepared (after 1 day) (18.82%) and aged control device (15.28%) (Table 1). The corresponding photovoltaic parameters for the composite HTL‐based devices are listed in **Table** **2**. The hysteresis index of the composite HTL‐based device increases with increasing the aging time (Figure S37 and Table S6, Supporting Information). However, the increment in hysteresis index is lower compared to the device based on the control spiro‐OMeTAD based device (Table S3, Supporting Information). The enhanced PCE was attributed to the significant improvement of V_OC_ to 1.15 V, *J_SC_* to 22.49 mA cm^−2^ and FF to 76.38%. To confirm the PCE value of the aged device based on composite HTL, the device was subjected to MPP tracking with continuous illumination for 200 seconds. As shown in Figure 5d, the device reaches a stabilized power output PCE of 19.73%. To verify the increase in *J_SC_* with increasing the aging time, the EQE studies were conducted on the fresh (1 day aged) and aged device (after 21 days) as shown in Figure 5e. It is evident that the EQE of the aged device exhibits enhancement across the entire range of wavelengths at which absorption occurs. Concurrently, the integrated *Jsc* values derived from the EQE spectra show an increase for the aged device. Furthermore, we have measured the conductivity of the control and composite‐HTL (fresh and aged films) to understand the increase in PCE with time. Figure 5f shows the current‐voltage (*I–V*) relations of ITO/HTM/Au films, from which slope conductivity can be calculated (Table S7, Supporting Information). The conductivity of the freshly prepared control and composite HTL films are 1.793 × 10^−5^ S cm^−1^ and 1.567 × 10^−5^ S cm^−1^, respectively. However, after aging, the conductivity of the control sample decreases to 1.653 × 10^−5^ S cm^−1^, while the conductivity of the composite HTL film increases to 1.921 × 10^−5^ S cm^−1^. Therefore, the increase in conductivity with time could be also responsible for boosting the PCE.

**Table 2:** Photovoltaic parameters of the composite HTL based devices under different aging times at dry air chamber (RH = 2%, 25 °C) in the backward direction.

HTL	Aging time		V_OC_ [V]	J_SC_ [mA cm^−2^]	FF [%]	PCE [%]
Composite HTL	Fresh	Champion	1.125	19.99	61.25	13.78
		Average	1.101 ± 0.010	19.90 ± 0.260	60.88 ± 0.51	13.24 ± 0.310
	7 days	Champion	1.132	20.68	73.38	17.15
		Average	1.123 ± 0.001	20.23 ± 0.060	72.15 ± 1.060	16.82 ± 0.330
	14 days	Champion	1.142	21.80	74.89	18.44
		Average	1.141 ± 0.001	21.38 ± 0.090	73.28 ± 1.37	17.78 ± 0.478
	21 days	Champion	1.150	22.49	76.38	19.70
		Average	1.148 ± 0.002	22.35 ± 0.052	74.14 ± 1.47	19.07 ± 0.462
	28 days	Champion	1.153	22.3	76.18	19.59
		Average	1.148 ± 0.003	22.30 ± 0.180	74.19 ± 1.43	18.97 ± 0.520

Next, we determined the HOMO level of the composite HTL films (fresh and aged) by UPS (Figure S38a, Supporting Information). Interestingly, the HOMO level of the composite HTL significantly shifts with increasing the aging time. The HOMO energy level for the fresh composite HTL film is −5.41 eV, while for the aged film is −5.64 eV, as indicated in Figure S38b (Supporting Information). Consequently, the aged film exhibits a profound HOMO level that is more aligned with the perovskite energy level and the improved band energy alignment can enhance hole extraction from the absorber layer to the HTL. Subsequently, we studied the alteration on the surface of the perovskite/composite‐HTL by AFM to understand the self‐enhancement of the PCE during the aging process. Interestingly, the AFM results show that the aged perovskite/composite HTL film exhibits a smoother surface morphology (RMS = 7.2 nm) compared to the respective fresh film (RMS = 8.5 nm) (Figure S39, Supporting Information) and aged control spiro‐OMeTAD film (Figure S34, Supporting Information). Therefore, the contact of HTL with perovskite becomes better after aging, which could enhance the photogenerated charge transfer efficiency.

To enhance our understanding of the hysteresis index and factors contributing to the observed increase in PCE over time, the devices were subjected to EIS characterizations. Figure S40a–d (Supporting Information) shows the EIS spectra of the fresh (1 day aged) devices with the control spiro‐OMeTAD and composite HTL as well as aged devices (21 days aged) with the control spiro‐OMeTAD and composite HTL. All the EIS measurements were collected under AM 1.5 G light illumination at different biases in the frequency range from 10^6^ to 0.1 Hz. Similar to the study performed in section 2.4, all the bias‐dependent EIS spectra contained two semicircles. Therefore, we fitted the spectra with the same equivalent circuit to get knowledge about the charge transport evaluation in the composite‐HTL based device and control device at different aging time. As expected, there is a noticeable increment in R_HF_ with time, which reconfirmed the lower recombination level at the interface in the case of aged composite HTL device (Figure S40e, Supporting Information). The higher conductivity of aged composite HTL enhances the efficient extraction of the photogenerated charge from the perovskite layer. As a result, losses due to charge recombination are reduced at the interface and in the bulk of the absorber layer leading to enhanced PCE. In the case of the control device, the value of R_HF_ reduces with increasing the aging time, indicating a higher level of recombination. This could be attributed to the over‐oxidation of spiro‐OMeTAD, which reduces the conductivity (Table S7, Supporting Information) and hole mobility of the spiro‐OMeTAD HTL layer leading to a decrease in PCE over time.^[^ ^61^ ^]^ The low‐frequency capacitance (C_LF_) is attributed to the ionic response, electrode polarization, and/or charge accumulation.^[^ ^62^, ^63^ ^]^ As shown in Figure S40f (Supporting Information), the higher C_LF_ of the aged devices (both control and composite‐HTL) compared to the fresh devices suggests an increase in ion accumulation with time. It is noted that the aged control spiro‐OMeTAD based device shows higher value of C_LF_ compared to aged composite HTL based cell. As reported, the *J–V* hysteresis is directly related to ion migration or accumulation. Therefore, the increment in hysteresis index and C_LF_ can suggest that the ion migration and charge accumulation in the device enhances with increasing the aging time. On the other hand, the higher level of *J–V* hysteresis and C_LF_ in the aged control spiro‐OMeTAD based device implies the higher level of ion accumulation and ion migration. Therefore, composite HTL not only enhances the PCE of PSCs but also suppresses the evolution of ion accumulation and ion migration in the devices.

### 2.6 Device Stability

**Figure 6:**
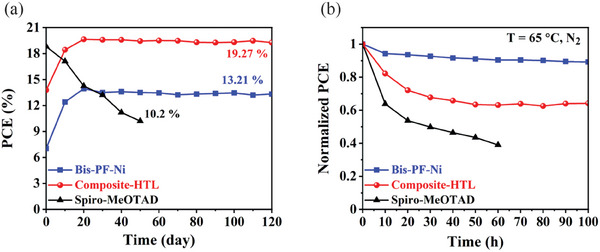
a) Shelf life stability of the devices with different HTMs at room temperature and relative humidity of ≈2%, b) Thermal stability of the devices with different HTMs at 65 °C under N_2_ atmosphere.

Long‐term stability is an essential property of PSCs for practical application.^[^ ^64^ ^]^ The devices based on Bis‐PF‐Ni, spiro‐OMeTAD and composite HTL were stored in a dry air chamber (RH = ≈2%, 25 °C) for more than 4 months, and we tracked their shelf life stability at a certain time (**Figure** **6**a). The Bis‐PF‐Ni based device shows promising long‐term stability maintaining 95% (from 13.94% to 13.21%) after 120 days of storage. Moreover, the device with a composite HTL reveals significantly improved stability reaching a PCE above 19%, whereas the PCE of the spiro‐OMeTAD‐based PSC significantly dropped (more than 50%) after 60 days. The improved stability of the devices with Bis‐PF‐Ni HTM could be attributed to its high hydrophobic character (high water contact angle of Bis‐PF‐Ni and composite HTL than spiro‐OMeTAD) as depicted in Figure 4a and Figure S41 (Supporting Information), which provides efficient protection against humidity. We further study the thermal stability of the devices by monitoring their performance at the temperature of 65 °C under an inert atmosphere (N_2_) (Figure 6b). After 60 hours, the spiro‐OMeTAD‐based device shows a notable decrease in efficiency, surpassing only 50% of its initial PCE. In contrast, devices containing Bis‐PF‐Ni HTM exhibited a notable temperature resistance where PSCs based on Bis‐PF‐Ni and composite HTL maintained more than 90% and 65% after 100 hours of its 21 days aged performance under similar conditions, respectively. The improved stability of the devices with Bis‐PF‐Ni could be attributed to its high thermal stability as depicted in Figure S14 (Supporting Information).

## 3 Conclusion

In conclusion, the effect of aging PSCs with Bis‐PF‐Ni HTM to understand the factors contributing to the self‐enhancement of the PCE was thoroughly studied. The results show that for an optimized concentration of Bis‐PF‐Ni the PCE of the device increases from an initial 7.04% to 13.94% after 21 days of storage. Notably, the improvement in PCE is only seen during storage of the devices in a dry environment, whereas no PCE increment is detected during their storage in an inert atmosphere. The impact of aging time on the changes in the perovskite layer, Bis‐PF‐Ni layer, perovskite/Bis‐PF‐Ni interface and the completed device was experimentally investigated using various methods. The age‐induced recrystallization of the perovskite film was excluded as a reason for the observed PCE self‐enhancement by analysis of its morphology and crystallinity before and after aging. Instead, it was found that the prolonged aging not only improves the conductivity of Bis‐PF‐Ni due to its liquid crystal property but also reduces its HOMO level facilitating better band alignment with the perovskite. Therefore, aging process gradually enhances the ability of Bis‐PF‐Ni to efficiently extract charge carriers from perovskite. Further analysis of the dark current and illumination‐dependent photovoltaic parameters revealed the reduction of trap−assisted recombination in the devices after the aging process. The reduction of barrier for the transport of photogenerated charge carriers in the aged devices was also supported by the EIS measurements. These findings indicate that the observed PCE self‐improvement upon aging the device is due to the improvement of the electrical properties of the Bis‐PF‐Ni layer and the quality of the perovskite/Bis‐PF‐Ni interface. In addition, the self‐enhancement in the PCE was also observed in the case of the addition Bis‐PF‐Ni to the spiro‐OMeTAD solution, where the PCE increased from 13.78% to 19.70% upon aging. All devices with Bis‐PF‐Ni exhibited superior ambient and thermal stabilities compared with spiro‐OMeTAD‐based devices, which was attributed to the high hydrophobicity and thermal stability of Bis‐PF‐Ni HTM. This work provides valuable insights into the factors that determine the self‐enhancement of the PCE upon the aging process, especially for devices with metal phthalocyanines.

## 4 Experimental Section

### Materials

Unless indicated, all compounds and solvents were utilized without additional purification. Chempure (Poland) provided the solvents. The following materials were purchased from Sigma‐Aldrich: lead iodide (PbI_2_, 99.9985%, trace metal basis), lead bromide (PbBr_2_) (98%), spiro‐OMeTAD (99%), bis(trifluoromethane)sulfonimide lithium salt (LiTFSI) (99.95%), 4‐tert‐butylpyridine (tBP), [N,N‐dimethylformamide (DMF) (anhydrous, 99.8%), dimethyl sulfoxide (DMSO) (anhydrous, 99.8%), chlorobenzene (CB) (anhydrous, 99.8%), and acetonitrile (anhydrous, 99.8%). Greatcell Solar Ltd. supplied the formamidinium iodide (>99.99%), methylammonium bromide (>99.99%), and FK 209 Co (III)TFSI (98%). Alfa Aesar supplied the tin (IV) oxide (SnO_2_) aqueous colloidal solution (15% in H_2_O colloidal dispersion), which contains SnO_2_ colloidal particles.

### Device Fabrication

Glass substrates made of indium tin oxide (ITO) were cleaned for 20 minutes using Helmanex, DI water, ethanol, acetone, and isopropyl alcohol, respectively, and then exposed to UV ozone for 20 minutes. Subsequently, a compact SnO_2_ layer with a thickness of about 20–30 nm was formed onto the ITO surface using spin coating at 4000 rpm for 30 s, followed by 30 minutes of annealing at 150 °C. A triple cation perovskite solution with the composition [Cs_0.05_(FA_0.95_MA_0.05_)_0.95_Pb(I_0.95_Br_0.05_)_3_] was prepared using the following ingredients: DMSO: DMF = 400 µL: 600 µL; CsI (50 µL from 1.5 M stock solution in DMSO), FAI (0.172 g), MABr (0.022 g), PbI_2_ (0.508 g), and PbBr_2_ (0.0807 g). Spin‐coating the perovskite solution included 10 s at 1000 rpm and 30 s at 6000 rpm. During the second step, chlorobenzene as anti‐solvent was poured on the spinning perovskite surface 15 s prior to the end of time. After that, every substrate was quickly moved to the hot plate and given an hour to anneal at 100 °C. Then, the Bis‐PF‐Ni HTM was dissolved in chlorobenzene and spin‐coated for 20 s at 4000 rpm. In case of making composite HTL, 5 mg of Bis‐PF‐Ni was added to spiro‐OMeTAD solution (70 mM) in 1 mL of chlorobenzene containing tBP, Li‐TFSI and FK209 solution in a molar ratio of spiro‐OMeTAD/FK209/Li‐TFSI/TBP of 1:0.03:0.5:3.3. Ultimately, the thermal evaporator was used to deposit 80 nm of the gold electrode.

### Current Density−Voltage Measurements

The photovoltaic performance was assessed using a Fluxim Litos Lite system, which included a Wavelabs Sinus LS2 solar simulator with an AM 1.5 spectrum for excitation. The current‐voltage characteristics were obtained by conducting forward and reverse scans at a scan rate of 50 mV s^−1^ on masking devices having a pixel size of 0.16 cm^2^. A calibrated reference solar cell made of crystalline silicon, along with a KG‐5 filter, was used to establish the light intensity at 100 mW cm^−2^. The power output was stabilized by using an MPP tracking algorithm for 200 seconds. Long‐term stability were measured after storing the devices in a dry air chamber (RH = ≈2%, 25 °C) for more than 4 months. Thermal stability experiments were maintained at a temperature of 45 ± 5 °C in a nitrogen (N_2_) environment.

### Computational Details

The molecular geometry of Bis‐PF‐Ni was optimized using the B3LYP with basis set of 6‐311+g(d,p) for C, H, N, O and LanL2DZ level of theory for Ni in the Gaussian 16 software. The optimized Cartesian coordinates were shown in Table S8 (Supporting Information). Frequency calculations were also conducted to verify the existence of stationary spots. The HOMO‐LUMO was computed using Avogadro's software, VMD and Multiwfn program.^[^ ^65^ ^]^

### Cyclic Voltammetry Measurements

The electrochemical measurements were conducted using a Bio‐Logic SP‐150e potentiostat electrochemical workstation. The samples were carried on a glassy‐carbon working electrode, while an Ag/AgCl reference electrode and a platinum‐wire auxiliary electrode were used. The measurements were performed in a deoxygenated solution of 0.1 M tetra‐n‐butylammonium hexafluorophosphate ([Bu_4_N]^+^[PF_6_]^−^) in CH_3_CN. Ferrocene was used as an internal reference to compare potentials with the ferrocenium/ferrocene (FeCp20/+) combination. The electrochemical properties of Bis‐PF‐Ni were examined by measuring the CV, using the following equations:

(1) $$\mathrm{HOMO}\left(\mathrm{eV}\right)=-4.8-\left(E_{\mathrm{onset}.\mathrm{oxi}}-E_{1/2}\left(\mathrm{ferrocene}\right)\right)$$

(2) $$\mathrm{LUMO}\left(\mathrm{eV}\right)=-4.8-\left(E_{\mathrm{onset}.\mathrm{red}}-E_{1/2}\left(\mathrm{ferrocene}\right)\right)$$

where E_onset.oxi_ and E_onset.red_ were the onset potentials of oxidation and reduction, respectively, assuming that the energy level of ferrocene was 4.8 eV below the vacuum level.

### X‐Ray Photoelectron Spectroscopy (XPS)

XPS analysis was performed using the ULVAC‐PHI VersaProbe 5000 spectrometer, which used monochromatic Al Ka radiation with a photon energy of 1486.6 electron volts (eV).

### Thermogravimetric Analysis (TGA)

Thermogravimetric analysis was acquired from a thermogravimetric analyzer (NETZSCH‐STA 449 F1).

### Scanning Electron Microscopy (SEM)

The field emission scanning electron microscopy (FE‐SEM) technique (S5500, Hitachi) was used to evaluate the top‐view and cross‐section SEM of all films.

### Contact angle measurements

Contact angle measurements were conducted using a Biolin Scientific Attention – Theta Lite contact‐angle device at room temperature, using water as the test liquid.

### Film Thickness Measurements

Reflectometer (Film metrics) was used to measure the thickness of thin films.

### Optical Polarizing Microscopy (POM)

Optical polarizing microscopy of the sample were determined with a Leitz Wetzler Orthoplan‐pol equipped with a hot stage (Linkam TMS 93) and temperature controller (Linkam LNP).

### Time‐Resolved Photoluminescence (TRPL)

Time‐resolved PL (TRPL) spectra were obtained by a fluorescence spectrometer equipped with a 405 nm pulsed laser (Edinburgh Instruments FS5 model Spectrometer).

### Ultraviolet Photoelectron Spectroscopy (UPS)

Ultraviolet photoelectron spectroscopy measurements of the films were collected with a UPS unit integrated into the PHI 5000 VersaProbe XPS system.

### External Quantum Efficiency (EQE)

EQE data were obtained at room temperature using a QE system (EnliTech) with monochromatic light focused on one pixel of the device with a cutoff frequency of 20 Hz.

### X‐Ray Diffraction (XRD)

XRD patterns of the film were obtained with PANalytical X'Pert Pro X‐ray powder diffractometer with Cu Kα radiation (λ = 1.54050 Å).

### Electrochemical Impedance Spectroscopy (EIS)

EIS measurements of all PSCs were performed on a self‐designed sample holder using Bio‐Logic SP‐150e potentiostat equipped with a frequency response analyzer under dark and AM 1.5 G light illumination condition as a function of applied bias and time.

### Atomic Force Microscopy (AFM)

Surface roughness values and surface topography of the films were obtained with an atomic force microscope (AFM) (Park System XE‐100E).

## Conflict of Interest

The authors declare no conflict of interest.

## Supporting information

Supporting Information

## Data Availability

The data that support the findings of this study are available from the corresponding author upon reasonable request.
