# Supplementary material for: Revealing the Impact of Aging on Perovskite Solar Cells Employing Nickel Phthalocyanine‐Based Hole Transporting Material
Source: Adv Sci (Weinh). 2024 Sep 16;11(41):2405284. doi: 10.1002/advs.202405284 (PMC11538661; doi:10.1002/advs.202405284)
Supplement: Supplementary file 1 — Supporting Information [file ADVS-11-2405284-s001.docx]

**Supporting Information**

**Revealing the Impact of Aging on Perovskite Solar Cells Employing Nickel Phthalocyanine-based Hole Transporting Material**

Muhammad Ans,*^a^* Zekeriya Biyiklioglu,*^b,^** Apurba Mahapatra,*^a^* Rohit D. Chavan,*^a^* Joanna Kruszyńska,*^a^* Muhittin Unal,^c^ Hilal Fazlı,*^b^* Kostiantyn Nikiforow,*^a^* Pankaj Yadav,^d,e^ Seckin Akin^c,f^, Emre Güzel^g,^* and Daniel Prochowicz^a,^*

*^a^ Institute of Physical Chemistry, Polish Academy of Sciences, Kasprzaka 44/52, 01-224 Warsaw, Poland*

*^b^ Department of Chemistry, Faculty of Science, Karadeniz Technical University, Trabzon, 61080, Türkiye*

*^c^ Laboratory of Advanced Materials & Photovoltaics (LAMPs), Necmettin Erbakan University, 42090, Konya, Türkiye*

*^d^ Department of Solar Energy, School of Energy Technology, Pandit Deendayal Energy University, Gandhinagar-382 007, Gujarat, India*

*^e^ Department of Physics, School of Energy Technology, Pandit Deendayal Energy University, Gandhinagar-382 007, Gujarat, India*

*^f^ Department of Metallurgical and Materials Engineering, Necmettin Erbakan University, 42090, Konya, Türkiye*

*^g^ Department of Engineering Fundamental Sciences, Faculty of Technology, Sakarya University of Applied Sciences, 54050, Sakarya, Türkiye*

**1. Materials and equipment**

All reagents and solvents were of reagent-grade quality and were obtained from commercial suppliers. The IR spectrum was recorded on a Perkin Elmer 1600 FT-IR Spectrophotometer, using KBr pellets. ^1^H and ^13^C NMR spectrum were recorded on Bruker Avance III 400 MHz spectrometers in CDCl_3_, DMSO-d*_6_* and chemical shifts were reported (d) relative to Me_4_Si as internal standard. MALDI-MS of nickel phthalocyanine complex was obtained using Bruker Microﬂex LT MALDI-TOF mass spectrometer Bremen, Germany). At room temperature, the UV-Vis absorption spectrum was recorded on the Perkin Elmer Lambda 25 UV-Vis spectrophotometer. Transition temperatures were determined with a Leitz Wetzler Orthoplan-pol equipped with a hot stage (Linkam TMS 93) and temperature-controller (Linkam LNP).

**2. Preparation of the phthalocyanines**

The synthesis of 2,3-bis(4-pentylphenoxy)propan-1-ol, 4-[2,3-bis(4-pentylphenoxy)propoxy]phthalonitrile and nickel(II) phthalocyanine bearing peripherally tetra-[2,3-bis(4-pentylphenoxy)propoxy] groups were shown in Figure 1. 2,3-bis(4-pentylphenoxy)propan-1-ol, 4-[2,3-bis(4-pentylphenoxy)propoxy]phthalonitrile were synthesized *via* substitution reactions. 2,3-Bis(4-pentylphenoxy)propan-1-ol was obtained form 4-pentylphenol, 2,3-dibromo-1-propanol, NaOH in ethanol. 4-[2,3-Bis(4-pentylphenoxy)propoxy]phthalonitrile was synthesized from 4-nitrophthalonitrile and 2,3-bis(4-pentylphenoxy)propan-1-ol in the presence of K_2_CO_3_ in DMF at 50 °C. Finally, nickel phthalocyanine (Bis-PF-Ni) was prepared by cyclotetramerization of the of 4-[2,3-bis(4-pentylphenoxy)propoxy]phthalonitrile with the presence of metal salt under nitrogen atmosphere (Figure S1). The synthesized compounds were characterized using FT-IR, ^1^H NMR, ^13^C NMR, UV–Vis, and MALDI-TOF mass techniques. In the FT-IR spectra, the formation of Bis-PF-OH was confirmed by the appearance of the aliphatic OH vibration frequency at 3419 cm^-1^. As expected, in the ^1^H NMR spectrum of Bis-PF-OH, the aliphatic OH signal appeared at 5.34 ppm. The ^13^C NMR spectrum of Bis-PF-OH showed signals for the new –CH-O-, -CH_2_-O, -CH_2_-OH carbon atoms at 72.13, 69.64, 66.34 ppm, respectively. The MALDI-TOF MS spectrum of Bis-PF-OH peaked at m/z 384.16 [M]^+^. In the FT-IR spectrum of Bis-PF-CN, the characteristic –C≡N peak was observed at 2231 cm^−1^. In the ^1^H NMR spectrum of Bis-PF-CN, the -OH proton signal of Bis-PF-OH disappeared and new aromatic signals were observed. Also, in the ^1^H NMR spectrum of Bis-PF-CN, aliphatic protons were observed in the range of 4-33-0.80 ppm. In the ^13^C NMR spectrum of 4-[2,3-bis(4-pentylphenoxy)propoxy]phthalonitrile (Bis-PF-CN) the specific nitrile carbon atoms were observed at 116.06 and 114.64 ppm. In the MALDI-TOF MS spectrum, 510.64 [M]^+^ peak also verified the structure of 4-[2,3-bis(4-pentylphenoxy)propoxy]phthalonitrile (Bis-PF-CN). The peak in the FT-IR spectrum for –C≡N vibrations of Bis-PF-CN (2231 cm^−1^) disappeared after conversion to nickel phthalocyanine (Bis-PF-Ni). The ^1^H NMR spectrum of the Bis-PF-Ni was measured in 400 MHz, CDCl_3_ at room temperature. While multiplet peaks in the range of 8.52–7.15 ppm indicate the presence of aromatic protons in the structure of Bis-PF-Ni, the peaks observed at around (4.61-4.58, 3.72, 2.54, 1.58, 1.29, 0.85 ppm) for Bis-PF-Ni, indicate the presence of -CH_2_-O, -CH-, Ar-CH_2_-, -CH_2_-, -CH_2_-, -CH_3_ protons. The carbon signals observed in the ^13^C NMR spectrum of Bis-PF-Ni confirm the structures of the Bis-PF-Ni. The mass spectrum of Bis-PF-Ni was obtained by the MALDI-TOF technique. The molecular ion peaks of Bis-PF-Ni were observed at 2101.79 as [M]^+^. The UV-Vis spectrum of Bis-PF-Ni exhibited characteristic Q and B bands which confirm the structure. The UV-visible spectrum of Bis-PF-Ni was recorded in CHCl_3_ at 1x10^-5^ M concentration (Figure 2a). The electronic spectrum of Bis-PF-Ni showed characteristic absorption bands at 674 nm and 329 nm in the Q band and B band regions which are characteristic for metallophthalocyanine in CHCl_3_. ^17–19^

**3.** **Synthesis and Characterization**


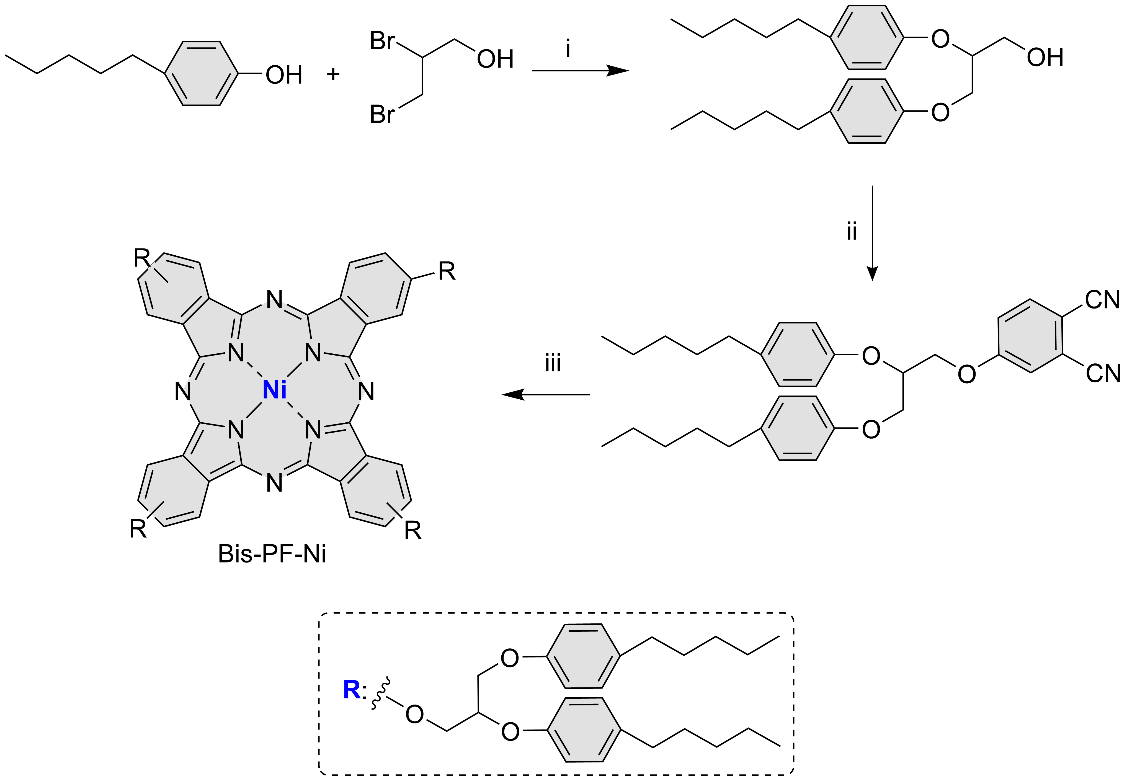


**Figure S1**. Molecular structures and synthetic pathway of peripherally tetra-[2,3-bis(4-pentylphenoxy)propoxy] substituted nickel phthalocyanine (Bis-PF-Ni). (i) NaOH, EtOH, 80 °C. (ii) 4-nitrophthalonitrile, K_2_CO_3_, 50 °C, DMF. (iii) 1-pentanol, NiCl_2_, DBU, 160 °C.

**3.1. Synthesis of 2,3-Bis(4-pentylphenoxy)propan-1-ol (Bis-PF-OH)**

4-Pentylphenol (3 g, 18.2 mmol) was added to ethyl alcohol (20 ml) and stirred for 10 min. Then, NaOH (913 mg, 22.8 mmol) was added and stirred at 50 °C for 1.5 h. 2,3-Dibromo-1-propanol (1.98 g, 9.1 mmol) in 3 ml ethanol was added dropwise to this solution with stirring for 30 min. The reaction mixture was stirred under nitrogen for 24 h at 80 °C. The solvent was removed under vacuum and crude product was washed with chloroform (150 mL), and water (100 mL). Using chloroform as an eluent, the pure compound was obtained by column chromatography (aluminum oxide). Yield: 2.20 g (63%). FT-IR (ATR) *ν* (cm^-1^): 3419 (O-H), 3030 (Ar-H), 2926-2856 (Aliph. C-H), 1611, 1509, 1458, 1378, 1297, 1239, 1176, 1114, 1041, 943, 822. ^1^H NMR (400 MHz, DMSO-d_6_), (δ): 7.04 (d, 4H, Ar-H), 6.83 (d, 4H, Ar-H), 5.34 (bs, 1H, O-H), 4.00-3.97 (m, 4H, -CH_2_-O), 3.43 (m, 1H, -CH-), 2.45 (t, 4H, Ar-CH_2_-), 1.50-1.46 (m, 4H, -CH_2_-), 1.24-1.20 (m, 8H, -CH_2_-), 0.81 (t, 6H, -CH_3_). ^13^C NMR (100 MHz, DMSO-d_6_), (δ): 157.04, 155.67, 134.72, 132.72, 129.50, 129.37, 115.38, 114.66, 72.13, 69.64, 66.34, 34.69, 31.36, 31.28, 22.43, 14.31. MALDI-TOF-MS m/z calc. 384.55; found: 384.16 [M]^+^.

- 1. **Synthesis of 4-[2,3-Bis(4-pentylphenoxy)propoxy]phthalonitrile (Bis-PF-CN)**

2,3-Bis(4-pentylphenoxy)propan-1-ol (1 g, 2.60 mmol) was dissolved in DMF (20 mL) and 4-nitrophthalonitrile (449 mg, 2.60 mmol) was added. Then, dry K_2_CO_3_ (898 mg, 6.5 mmol) was added to this mixture. The mixture was stirred under N_2_ atmosphere at 50 °C for four days. 4-[2,3-Bis(4-pentylphenoxy)propoxy]phthalonitrile was obtained using column chromatography with aluminum oxide (chloroform as solvent). Yield: 702 mg (53%). FT-IR (ATR) *ν* (cm^-1^): 3033 (Ar-H), 2926-2856 (Aliph. C-H), 2231 (C≡N), 1596, 1562, 1509, 1489, 1457, 1293, 1236, 1176, 1099, 1046, 974, 824. ^1^H NMR (400 MHz, DMSO-d_6_), (δ): 8.00 (d, 1H, Ar-H), 7.85 (s, 1H, Ar-H), 7.55 (d, 1H, Ar-H), 7.05 (d, 4H, Ar-H), 6.81 (d, 4H, Ar-H), 4.33-4.28 (m, 4H, -CH_2_-O), 3.45-3.41 (m, 1H, -CH-), 2.44 (t, 4H, Ar-CH_2_-), 1.49-1.45 (m, 4H, -CH_2_-), 1.25-1.18 (m, 8H, -CH_2_-), 0.80 (t, 6H, -CH_3_). ^13^C NMR (100 MHz, DMSO-d_6_), (δ): 161.76, 156.52, 136.16, 135.28, 129.59, 129.53, 129.51, 121.74, 121.40, 116.74, 116.06, 114.77, 114.73, 114.67, 114.64, 106.79, 76.33, 68.49, 67.07, 34.63, 31.29, 31.22, 22.39, 14.32. MALDI-TOF-MS m/z calc. 510.66, found: 510.64 [M]^+^.

#### **Synthesis of 2(3), 9(10), 16(17), 23(24)-tetrakis[2,3-bis(4-pentylphenoxy)propoxy]phthalocyaninato nickel(II) (Bis-PF-Ni)**

4-[2,3-Bis(4-pentylphenoxy)propoxy]phthalonitrile (100 mg, 0.19 mmol), NiCl_2_ (18 mg, 0.09 mmol), 1-pentanol (2 mL) and 1,8-Diazabicyclo(5.4.0)undec-7-ene (DBU) (3 drops) was stirred at 160 °C for 24 h. The green product was precipitated with ethanol. The crude product was purified using column chromatography with an aluminum oxide (chloroform as solvent). Yield: 64 mg (31%). FT-IR (ATR) n_max_/cm^-1^: 3032 (Ar-H), 2924-2854 (Aliph. C-H), 1609, 1584, 1509, 1466, 1416, 1349, 1228, 1176, 1117, 1093, 1044, 969, 820, 750. ^1^H NMR (400 MHz, CDCl_3_), (δ): 8.52-8.49 (m, 16H, Ar-H), 7.51 (m, 8H, Ar-H), 7.15 (m, 20H, Ar-H), 4.61-4.58 (m, 16H, -CH_2_-O), 3.72 (t, 4H, -CH-), 2.54 (m, 16H, Ar-CH_2_-), 1.58 (m, 16H, -CH_2_-), 1.29 (m, 32H, -CH_2_-), 0.85 (m, 24H, -CH_3_). ^13^C NMR (100 MHz, CDCl_3_), (δ): 159.16, 159.13, 156.85, 143.23, 137.46, 135.60, 129.41, 129.35, 122.65, 119.66, 114.65, 114.54, 77.20, 67.21, 67.18, 35.09, 31.43, 29.69, 22.54, 14.04. UV-Vis (CHCl_3_): λ_max_, nm (log ε) 674 (5.00), 609 (4.48), 329 (4.61). MALDI-TOF-MS m/z calc. 2101.35; found: 2101.79 [M]^+^.


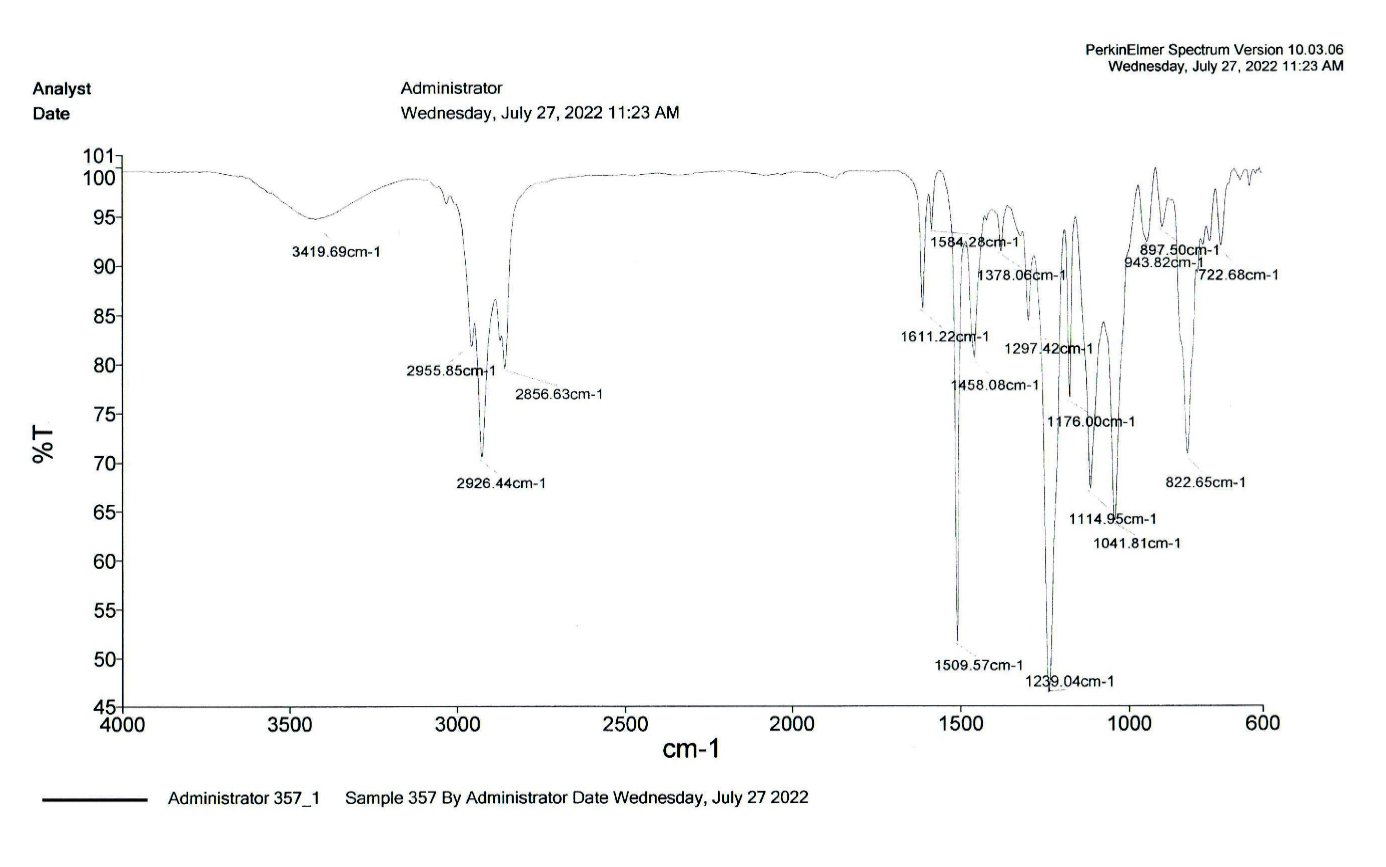


**Figure S2**. FT-IR spectrum of **Bis-PF-OF**.


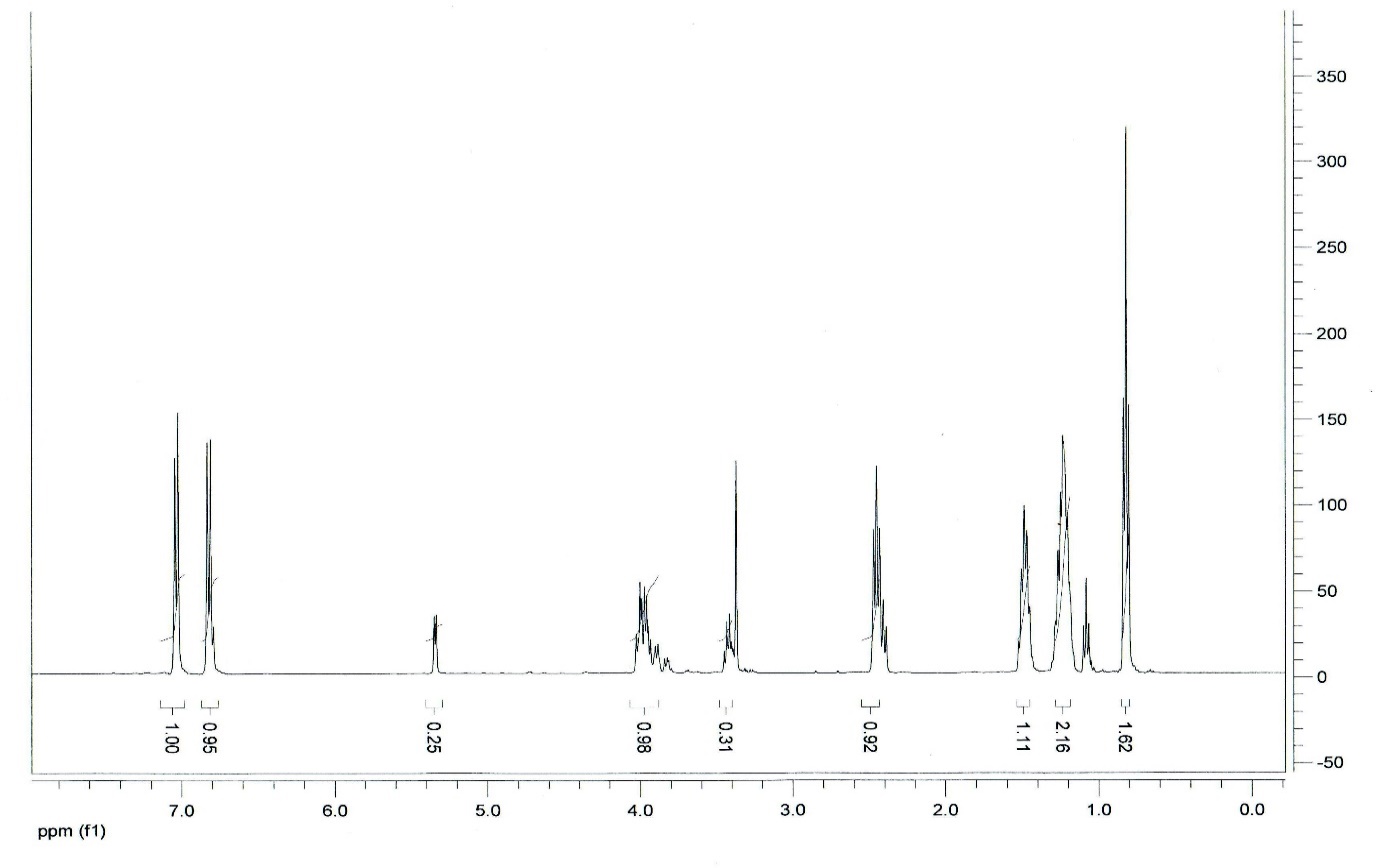
**Figure S3.** ^1^H NMR spectrum of **Bis-PF-OF**.


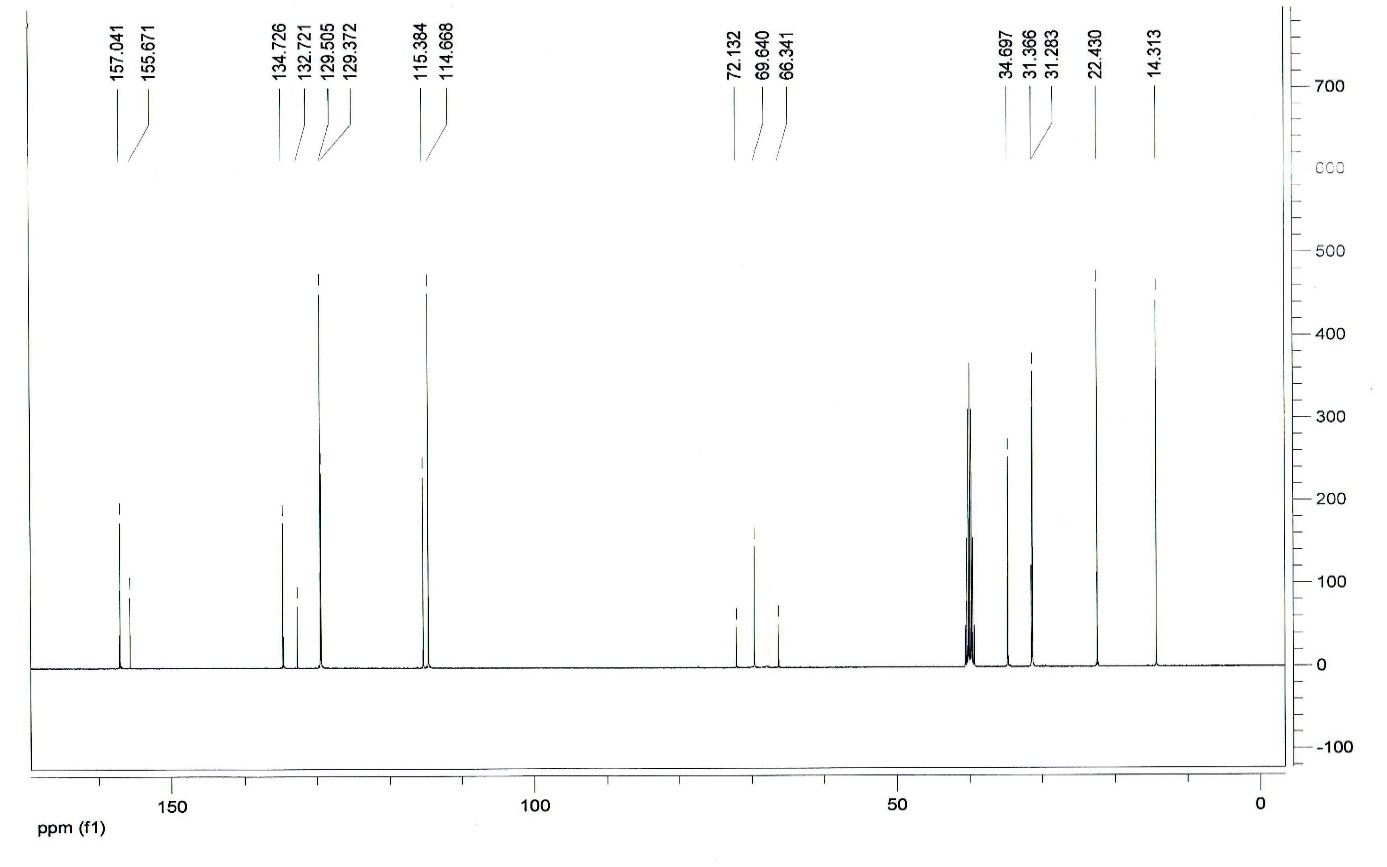
**Figure S4.** ^13^C NMR spectrum of **Bis-PF-OF**.


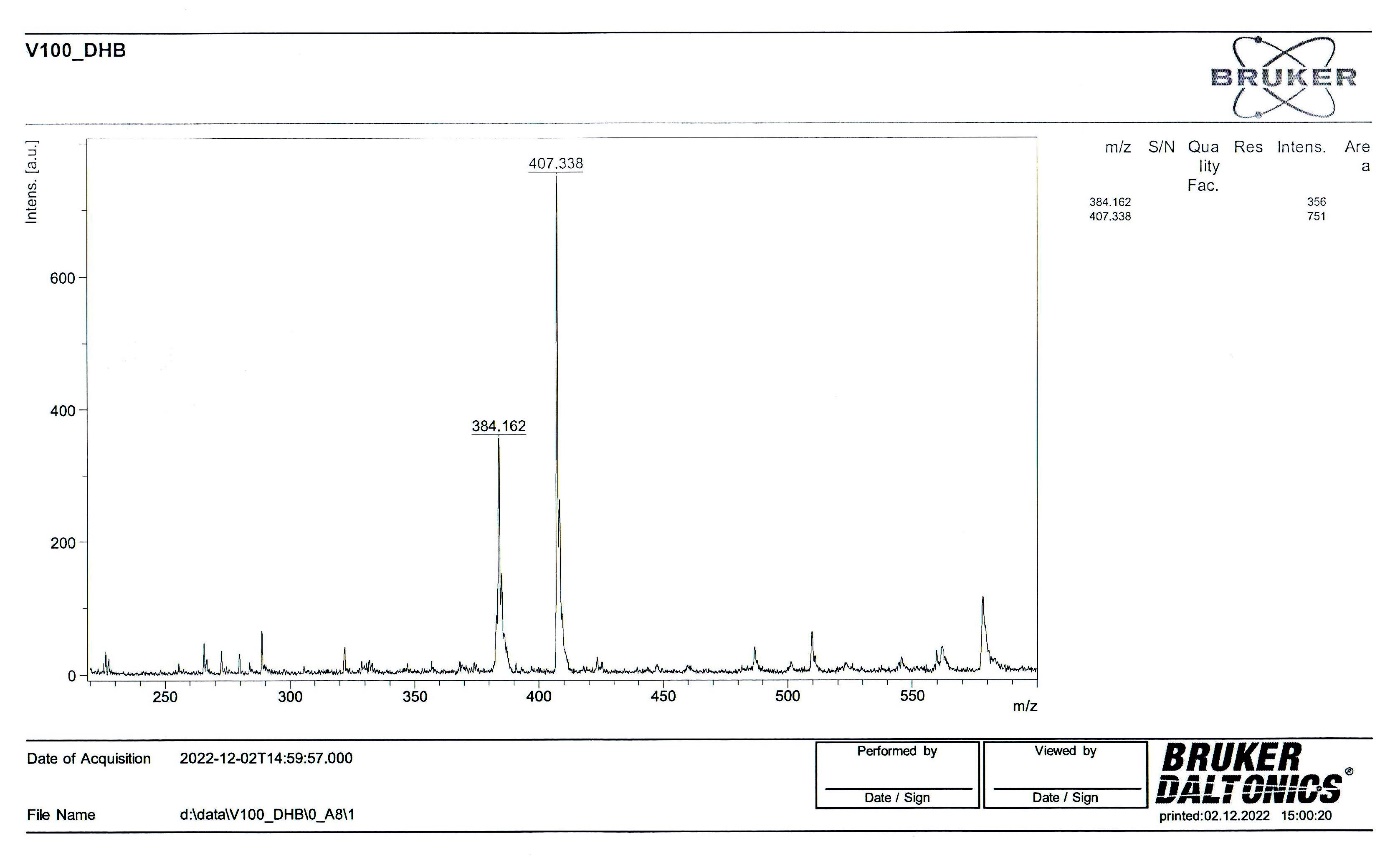
**Figure S5.** MALDI-TOF MS spectrum of **Bis-PF-OF**.


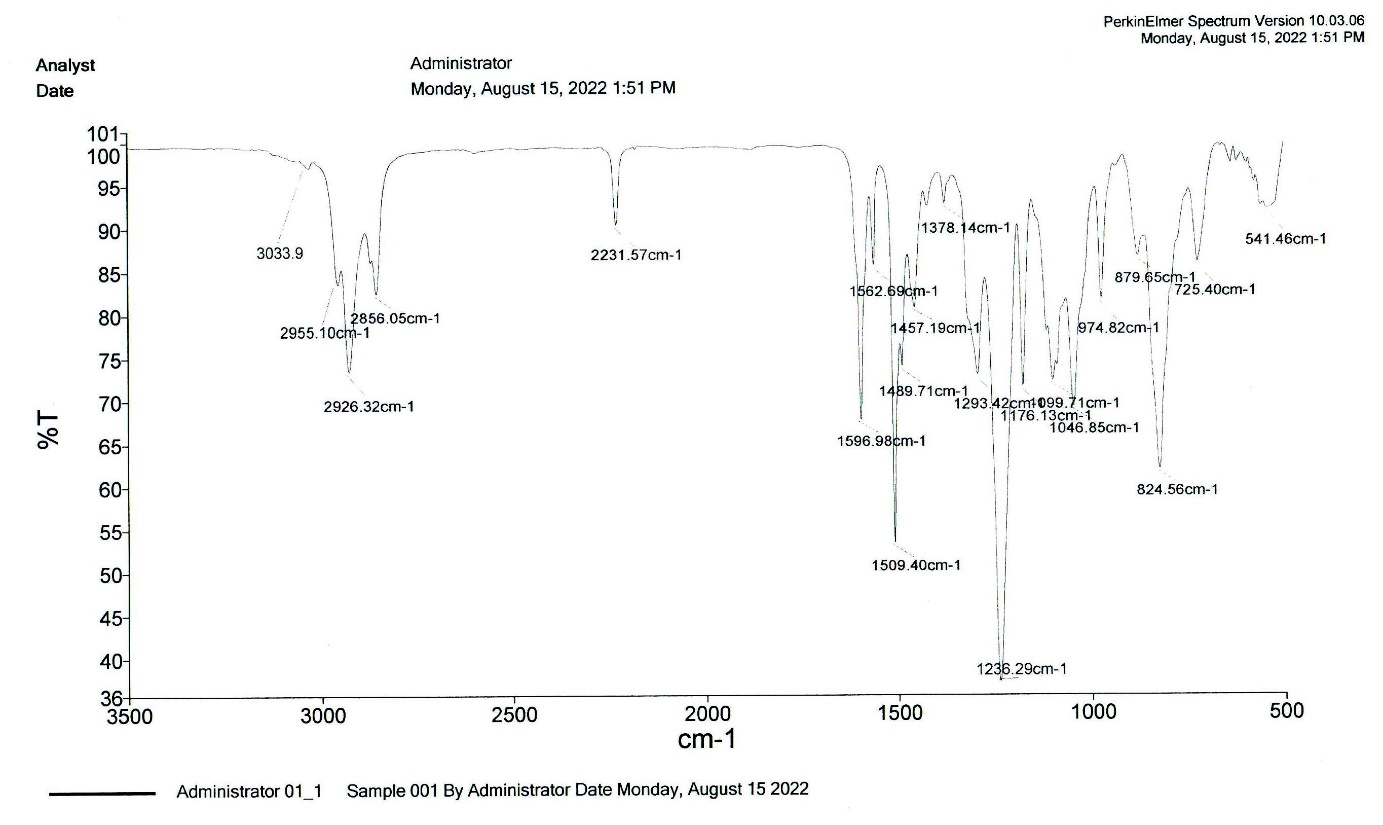
**Figure S6**. FT-IR spectrum of **Bis-PF-CN**.


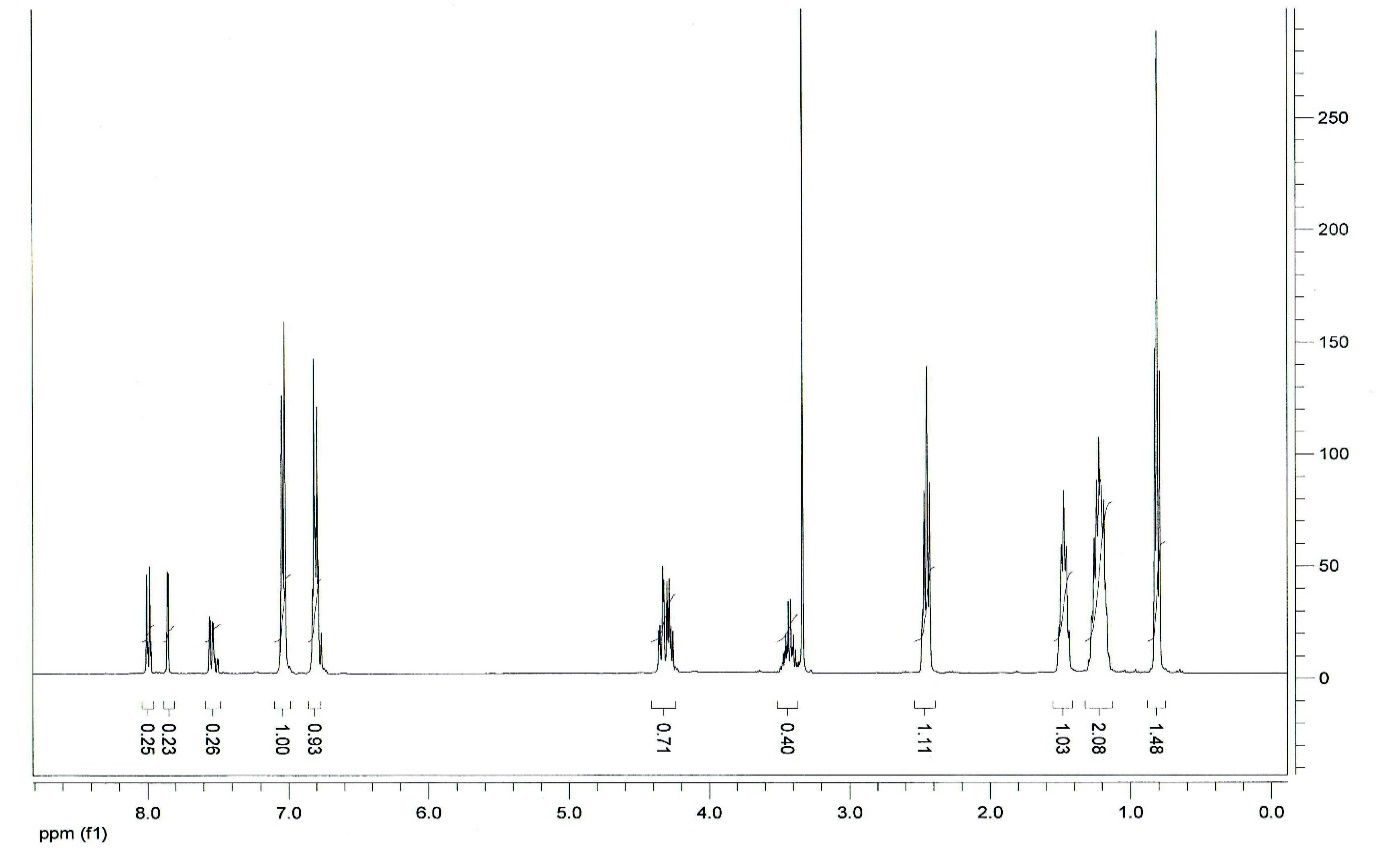


**Figure S7.** ^1^H NMR spectrum of **Bis-PF-CN**.


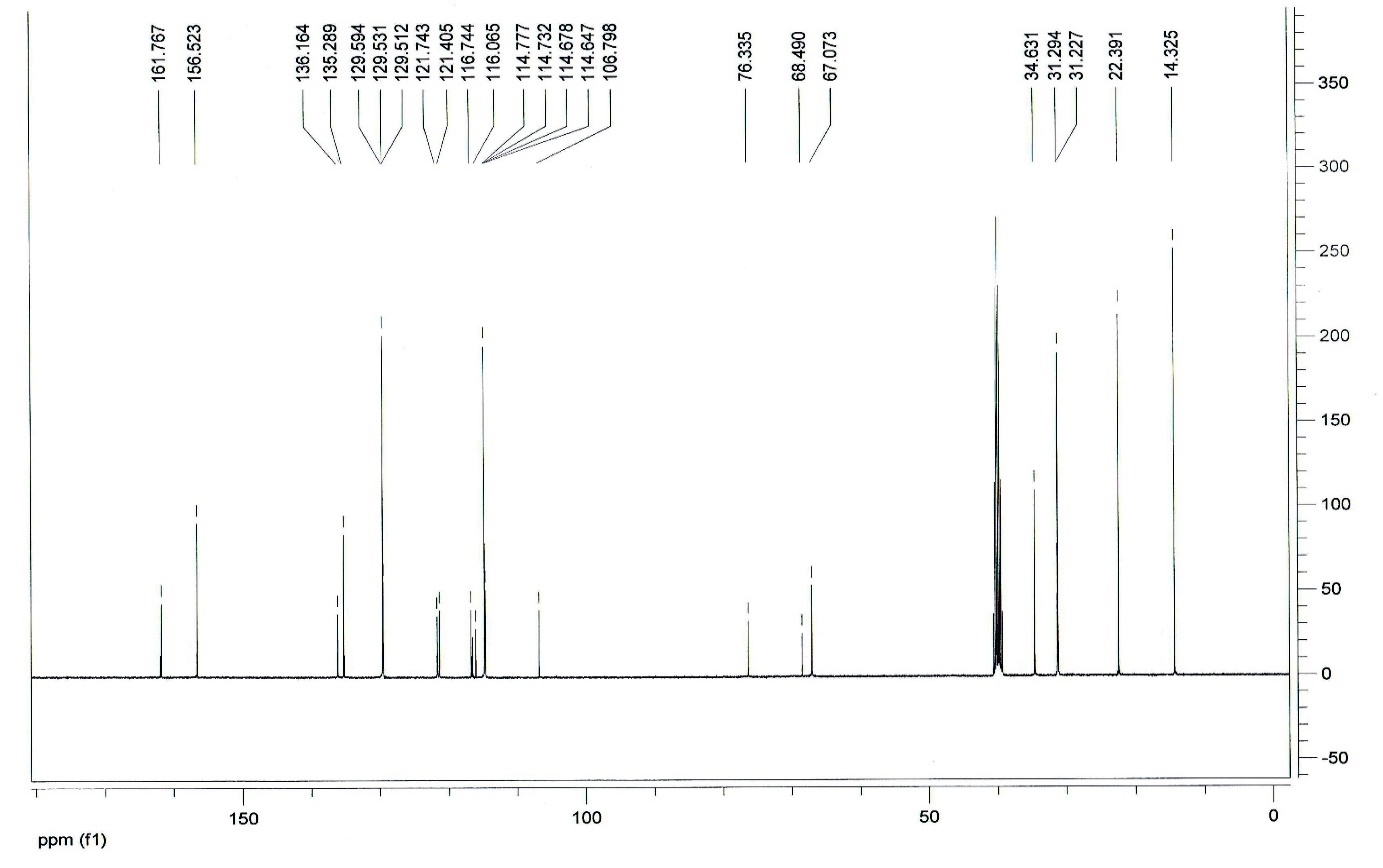
**Figure S8.** ^13^C NMR spectrum of **Bis-PF-CN**.


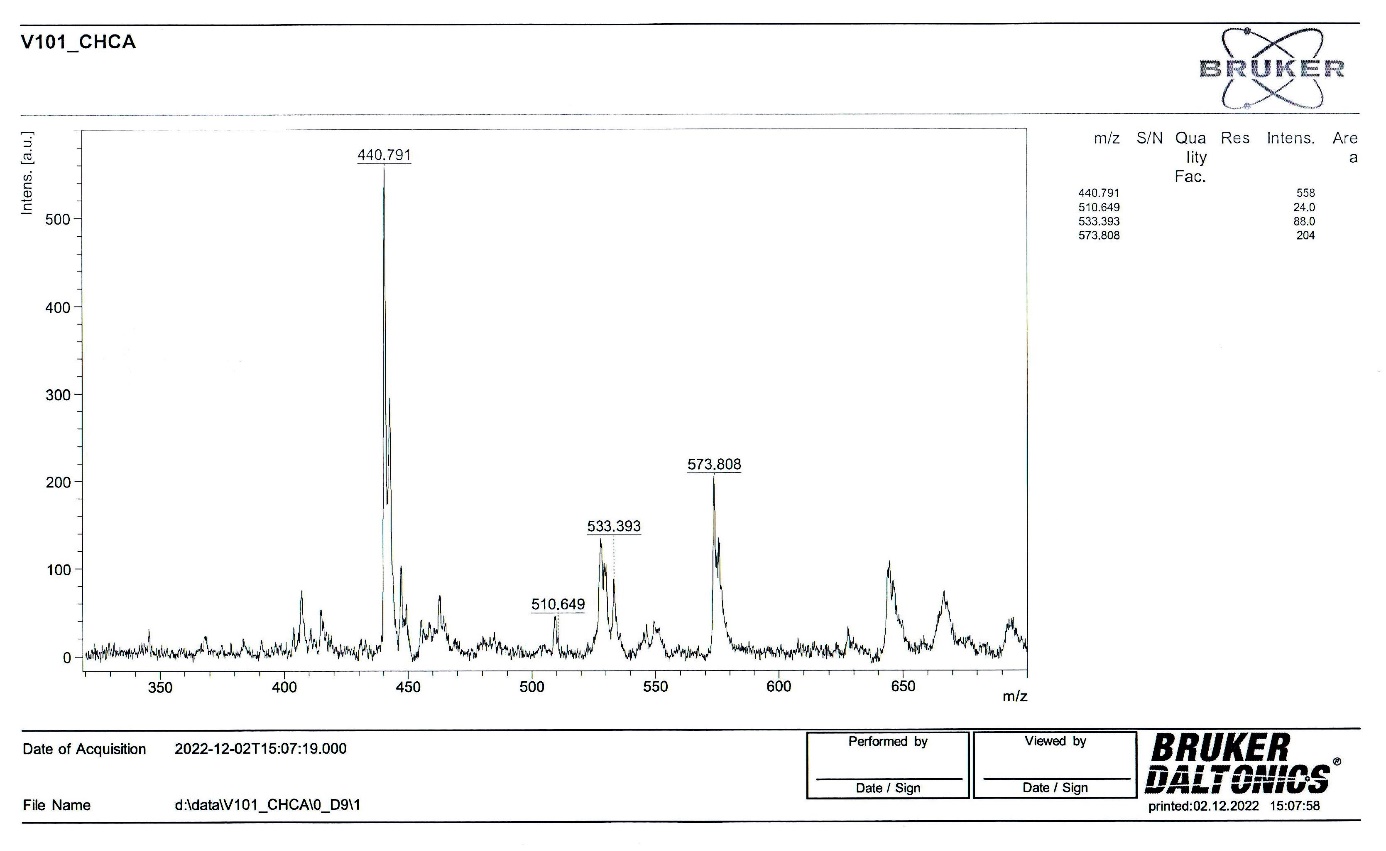
**Figure S9.** MALDI-TOF MS spectrum of **Bis-PF-CN**.


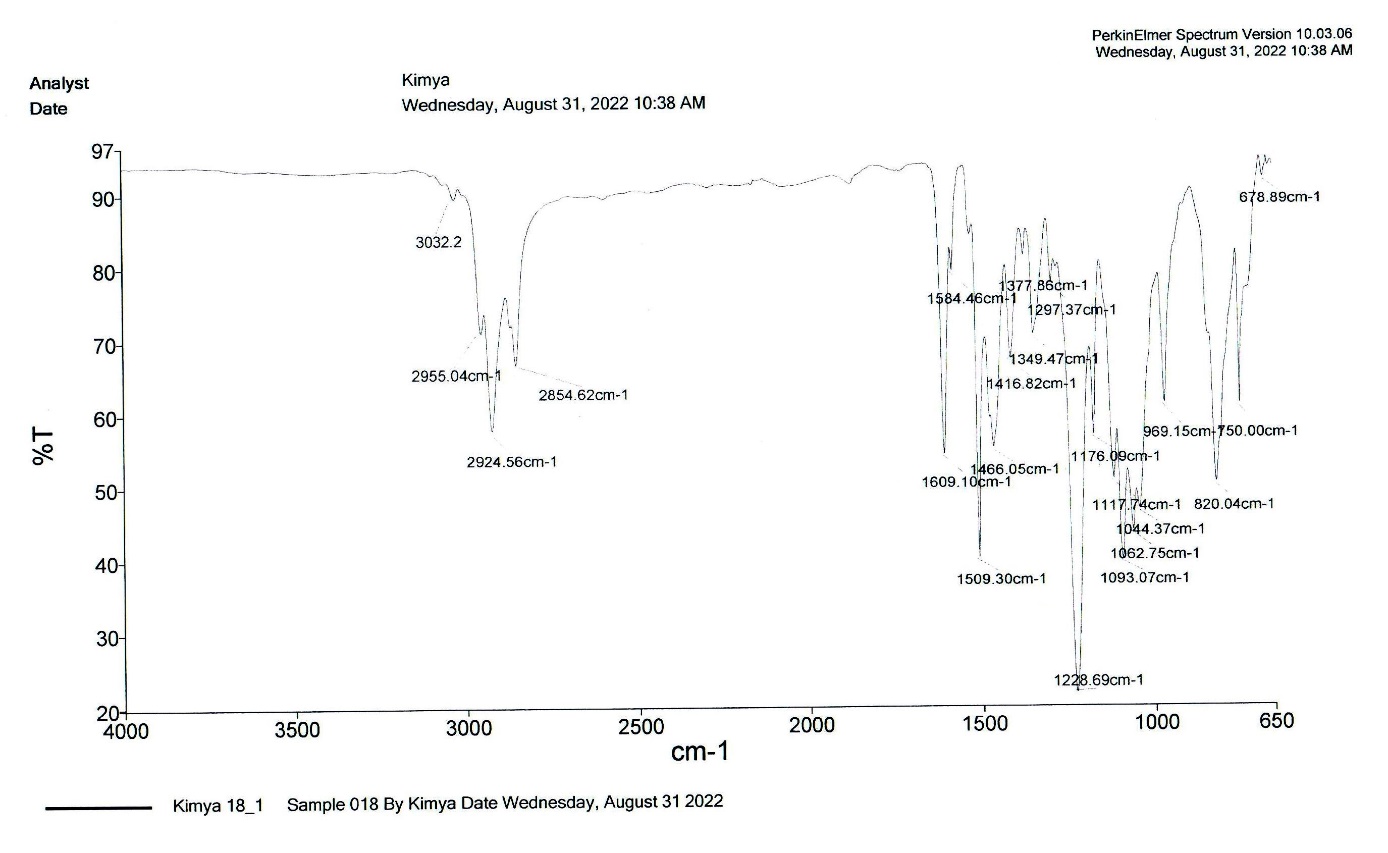
**Figure S10.** FT-IR spectrum of **Bis-PF-Ni**.


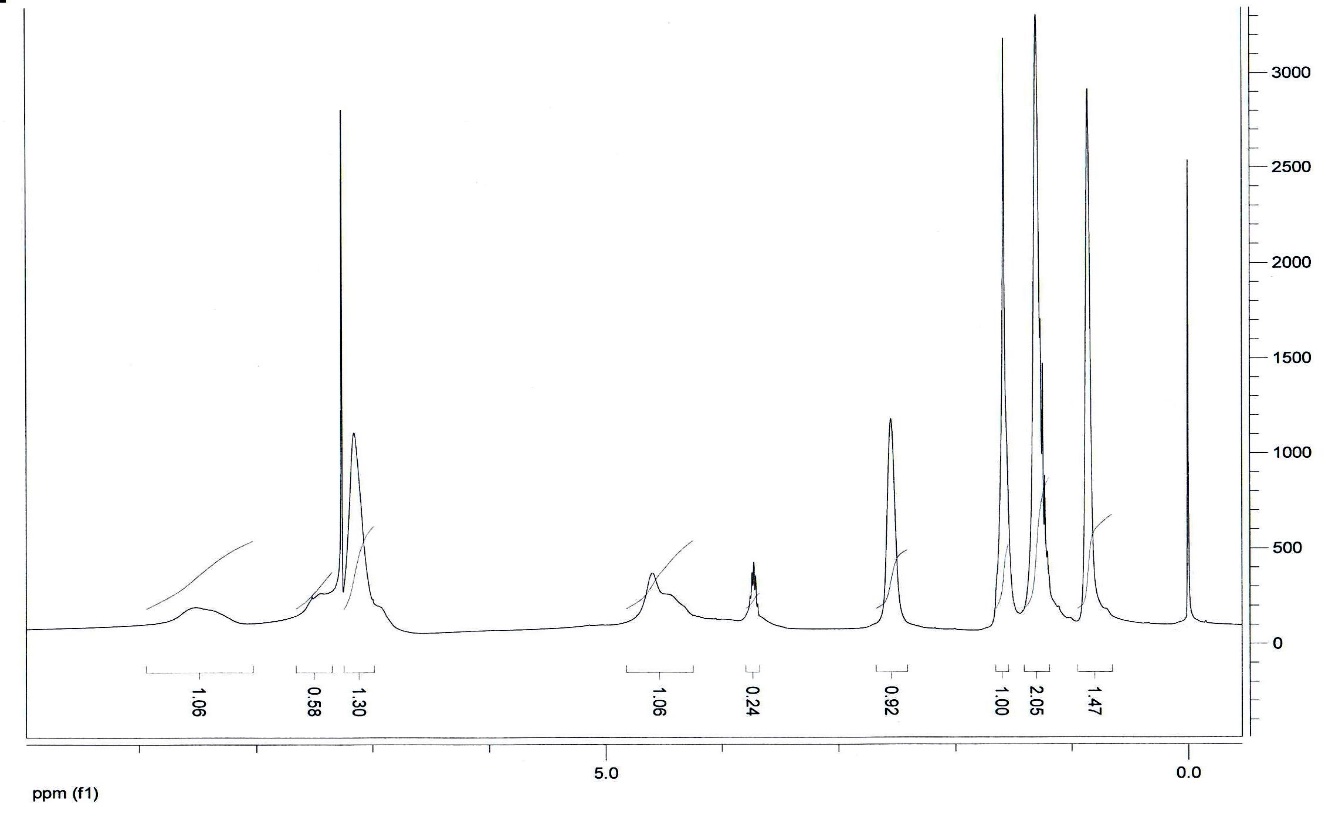


**Figure S11**. ^1^H NMR spectrum of **Bis-PF-Ni**.


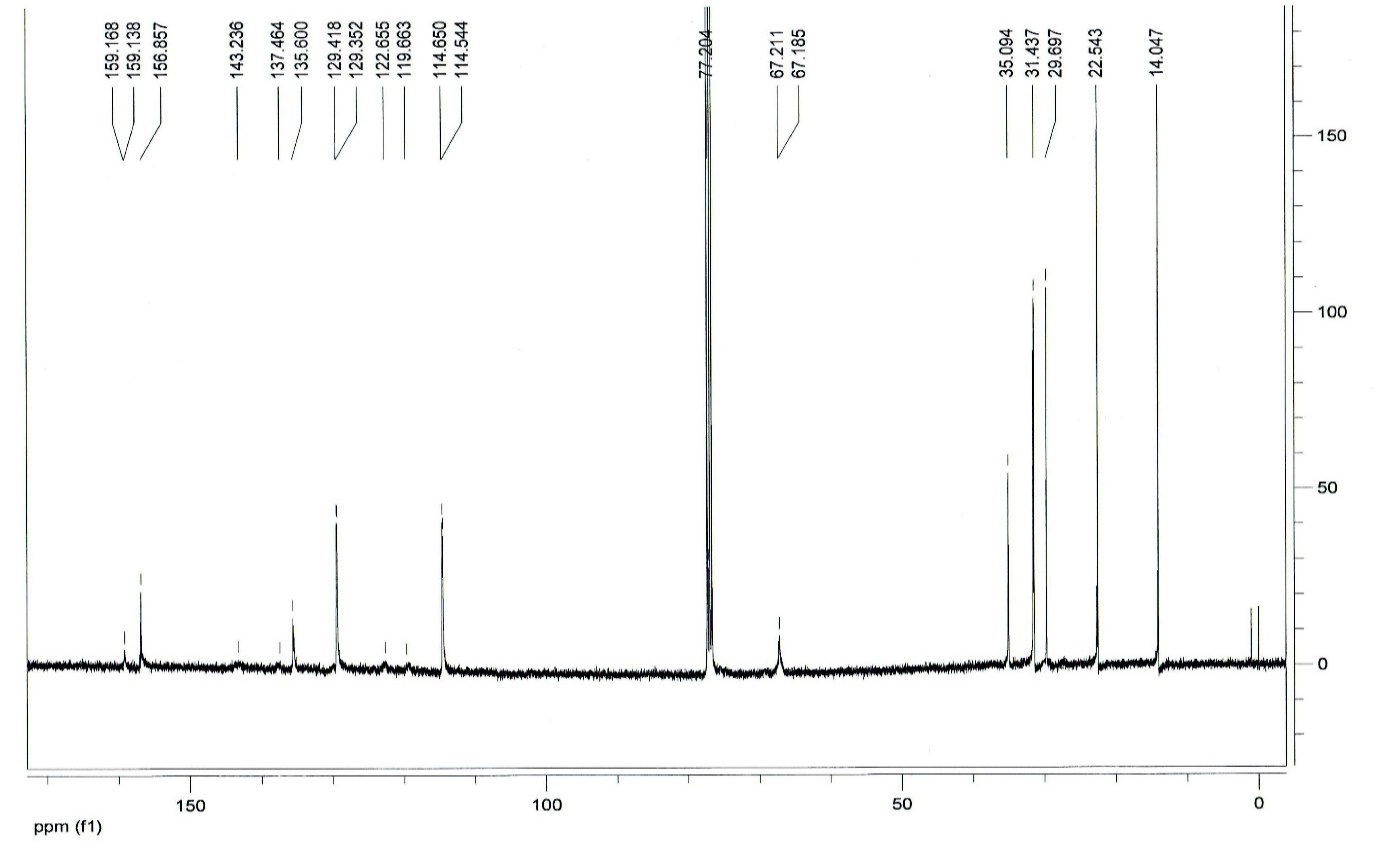
**Figure S12.** ^13^C NMR spectrum of **Bis-PF-Ni**.


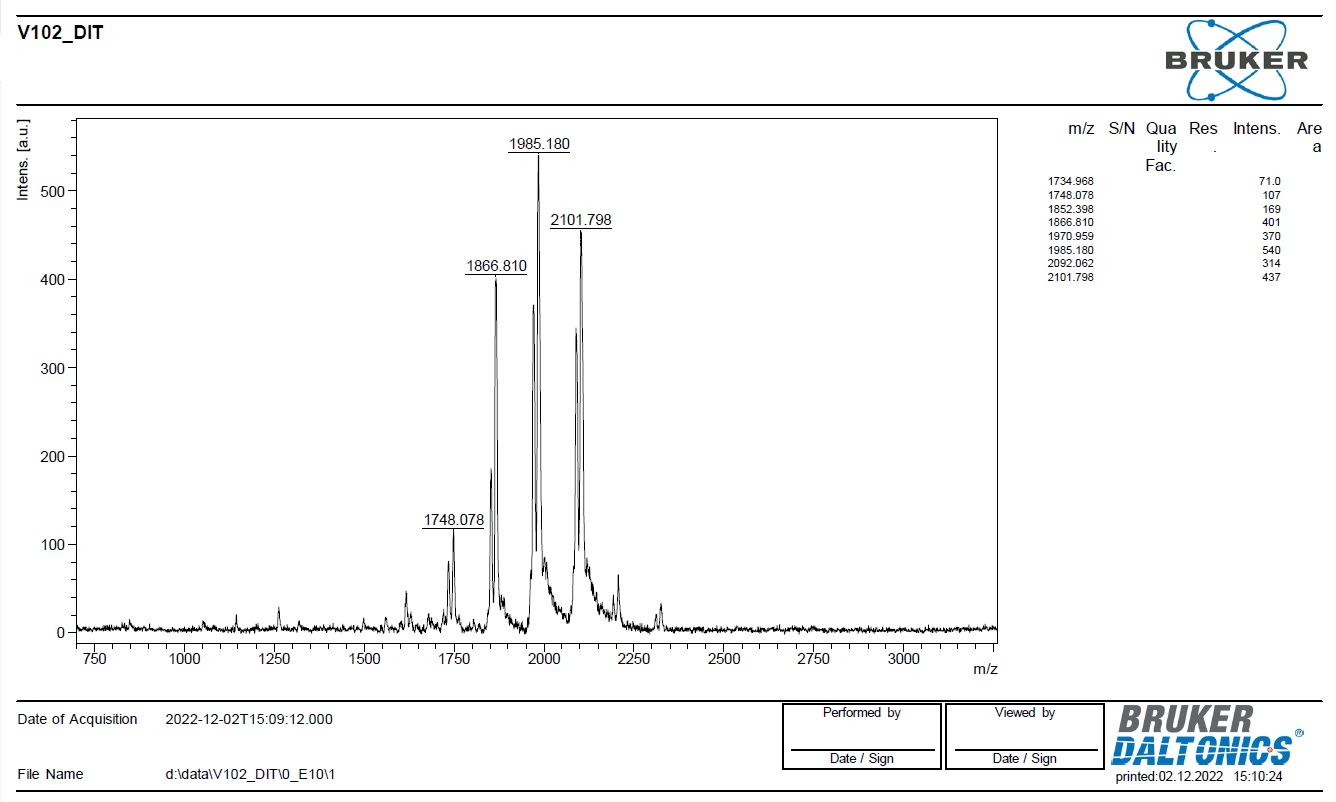
**Figure S13.** MALDI-TOF MS spectrum of **Bis-PF-Ni**.

**
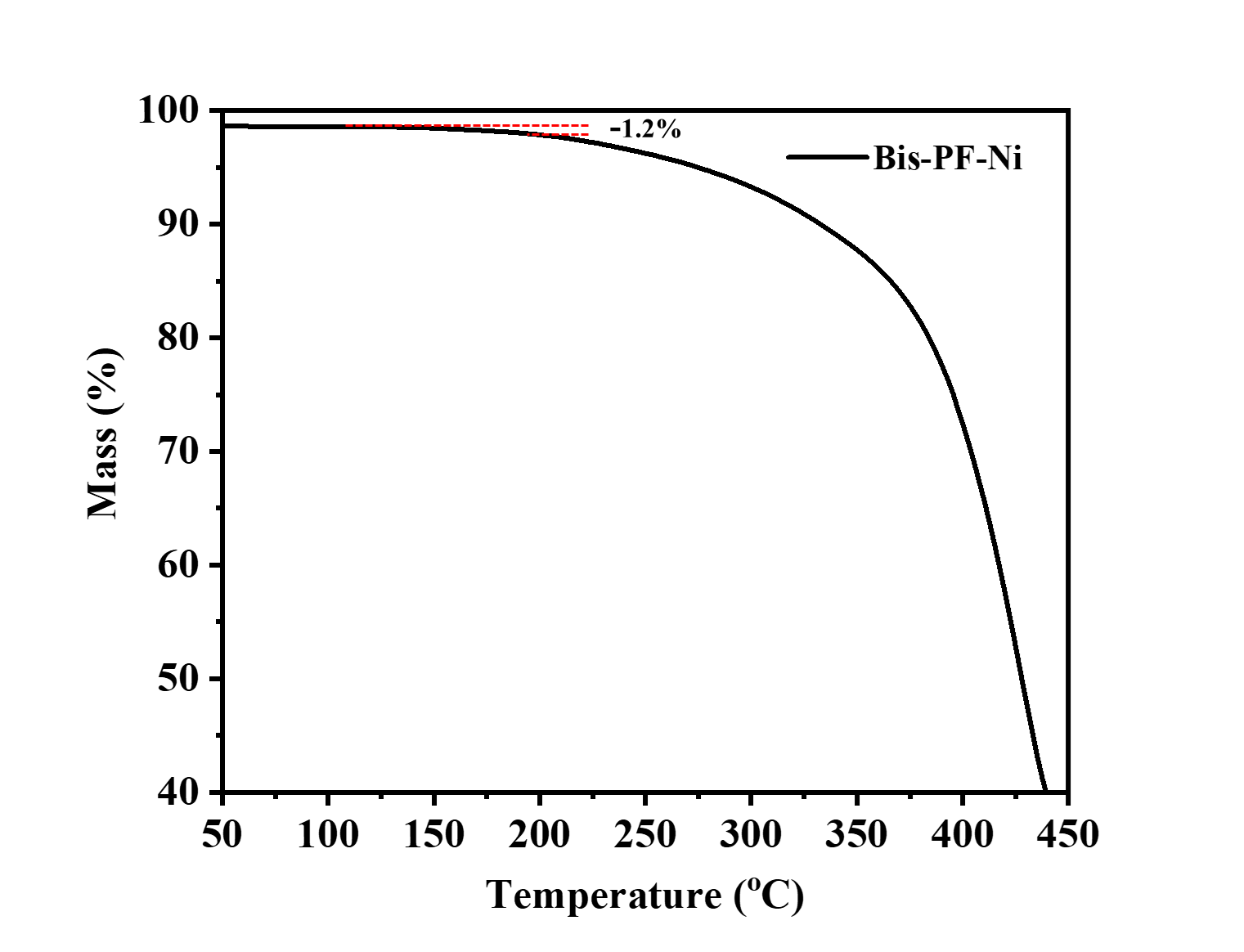
**

**Figure S14.** TGA curve of Bis-PF-Ni.


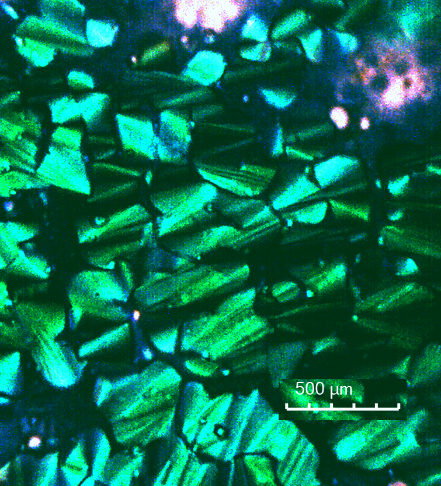


**Figure S15**. Photomicrographs of typical textures of mesophase (under crossed polarizers) of Bis-PF-Ni.


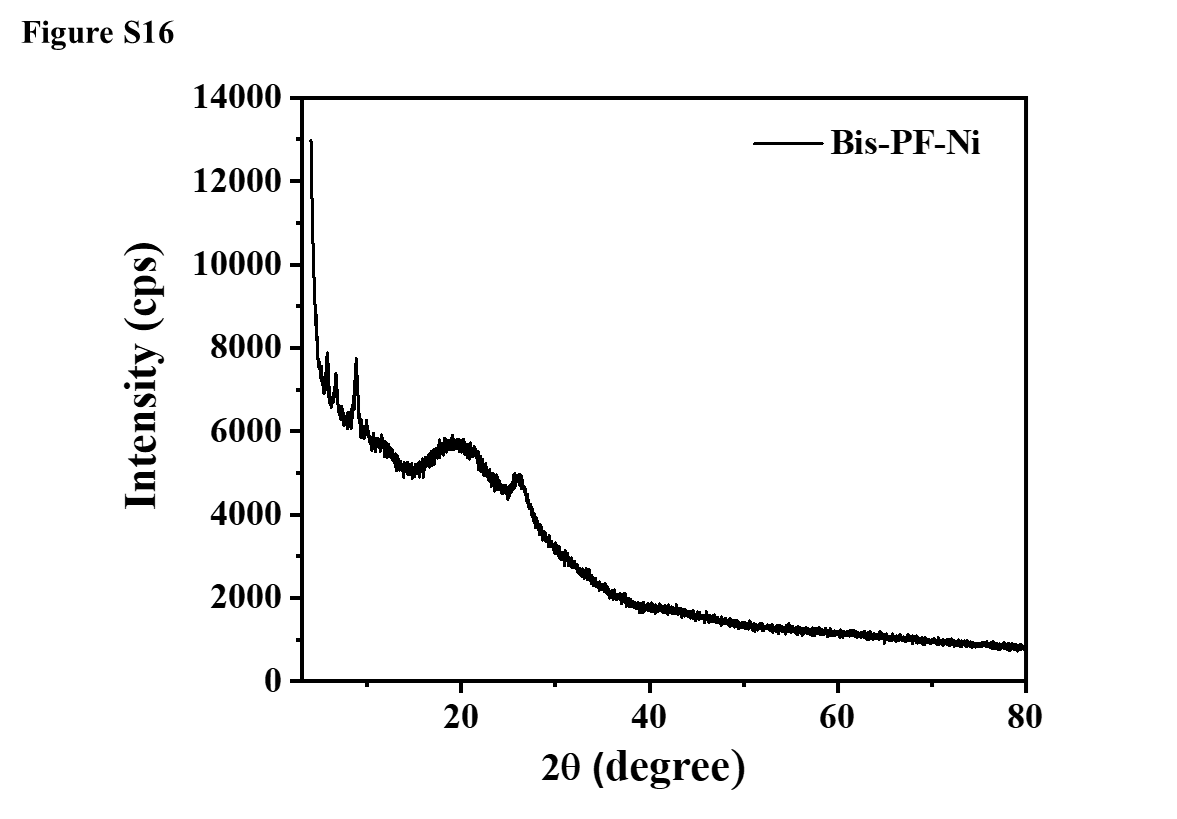


**Figure S16**. XRD pattern of Bis-PF-Ni at room temperature.


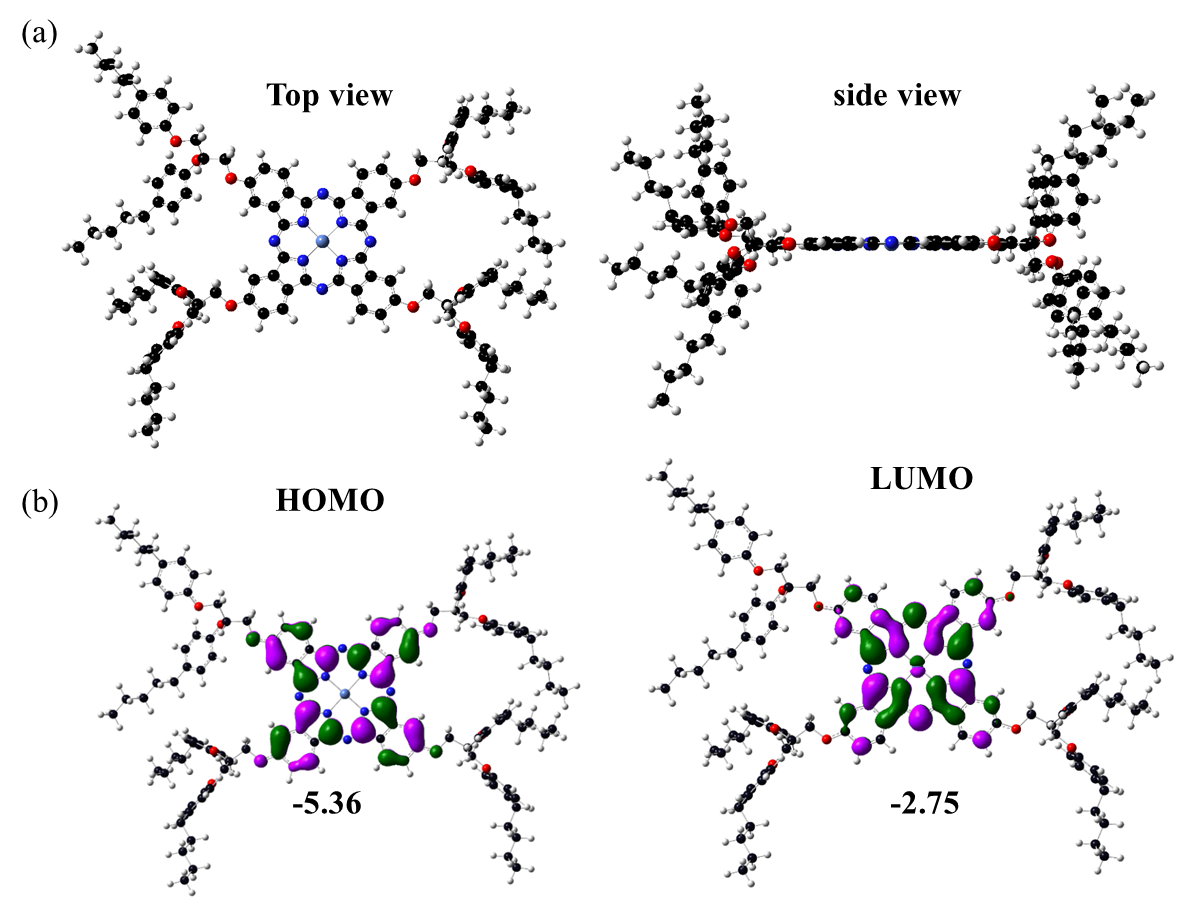


**Figure S17.** (a) Optimized geometry of Bis-PF-Ni at B3LYP with basis set of 6-311+g(d,p) for C, H, N, O and LanL2DZ for Ni. (b) Frontier molecular orbital analysis of Bis-PF-Ni HTM.


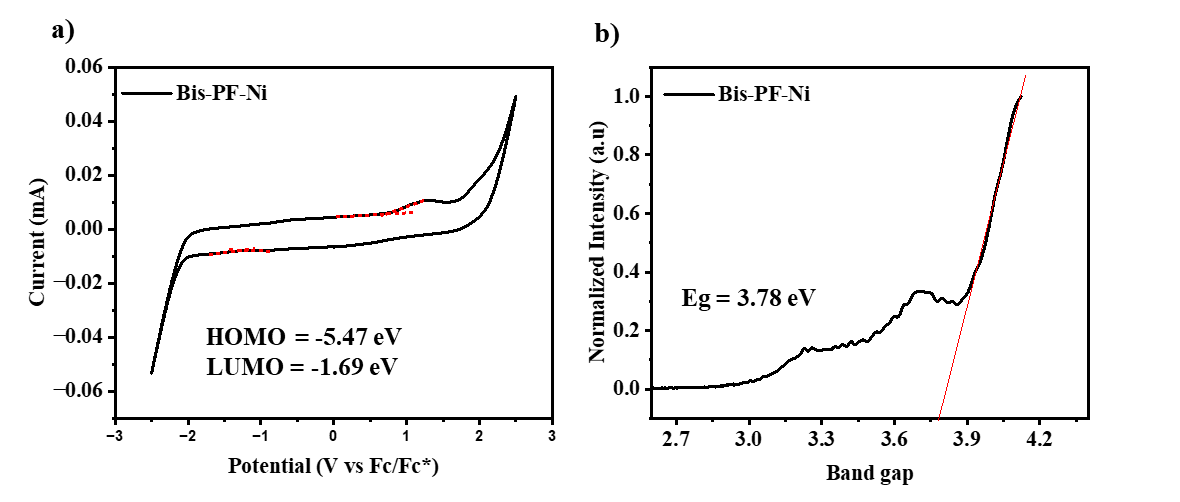


**Figure S18.** a) Cyclic voltammograms of Bis-PF-Ni. b) Optical bandgap of Bis-PF-Ni from the UV absorption edge.


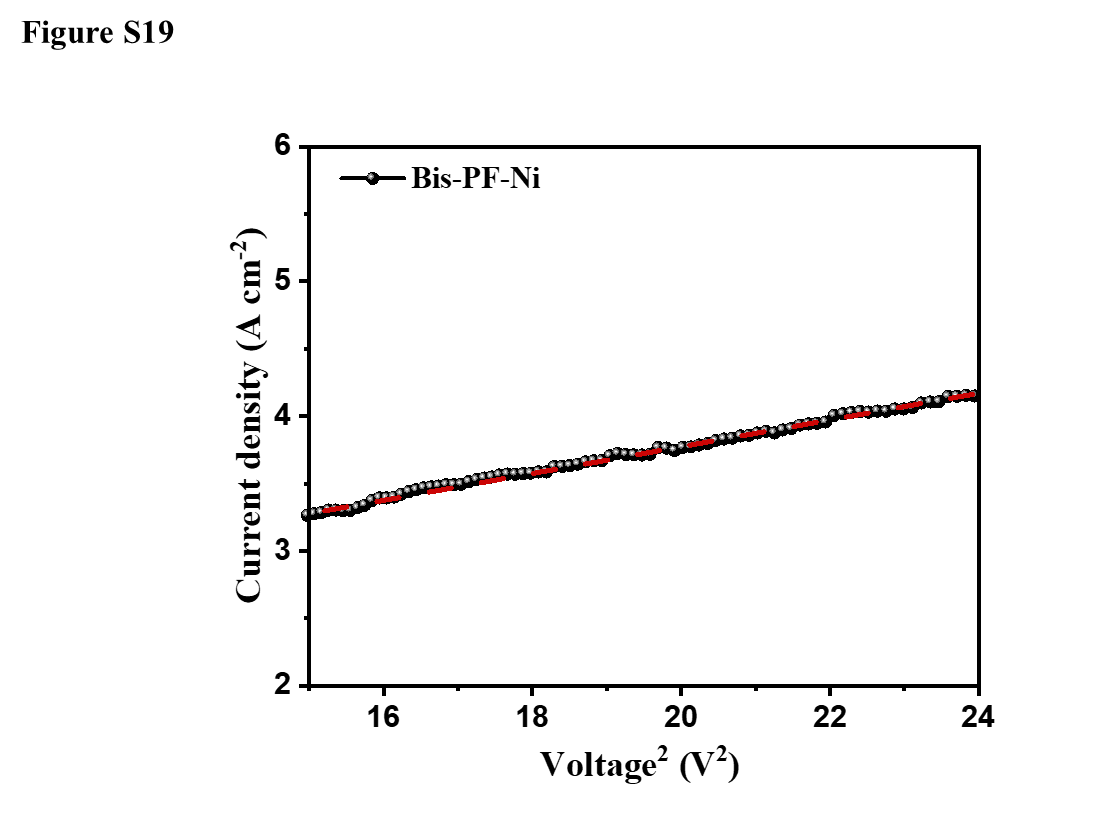


**Figure S19.** *J-V* curve of hole-only device (ITO/PEDOT:PSS/Bis-PF-Ni/Au) with a SCLC fitting for hole mobility.

**
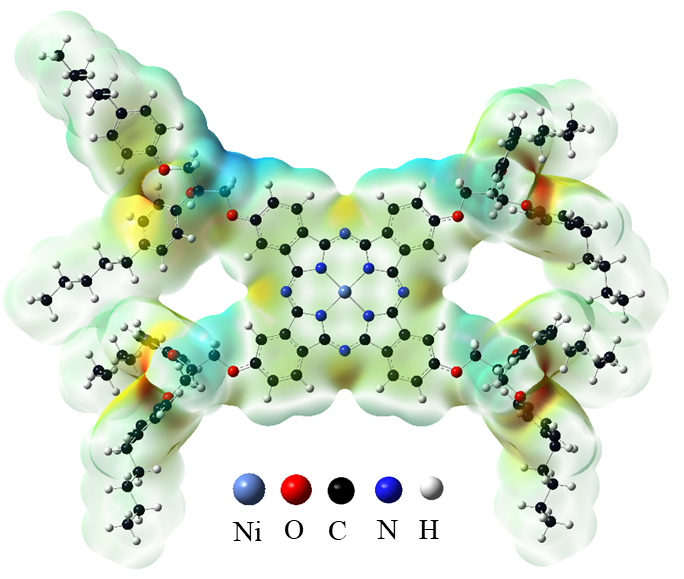
**

**Figure S20**. ESP analysis of the Bis-PF-Ni.


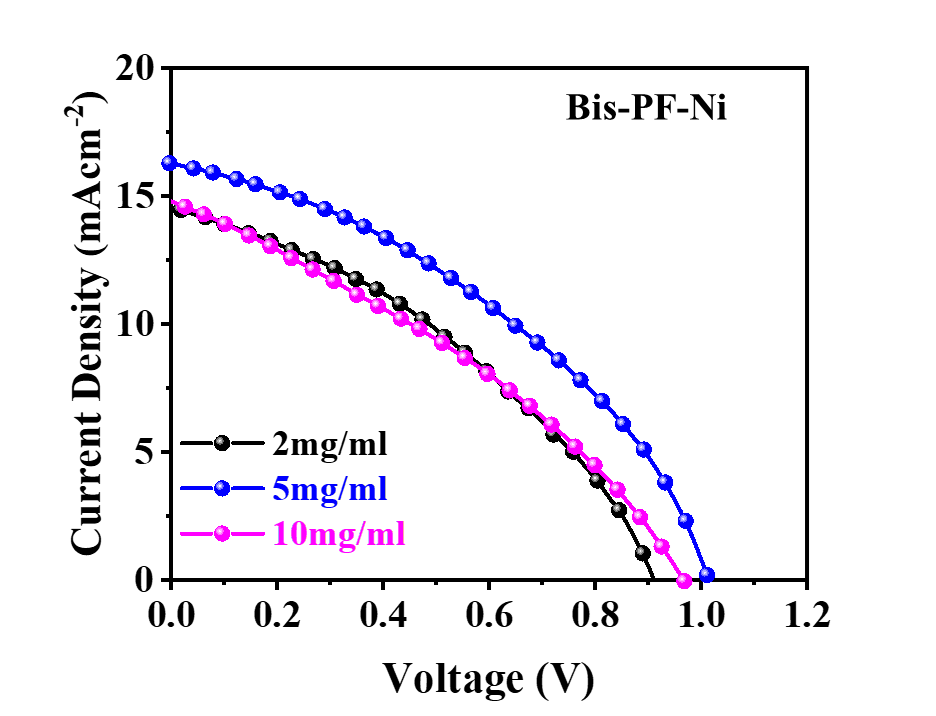


**Figure S21**. *J-V* curves of Bis-PF-Ni (dopant free) at different concentrations.


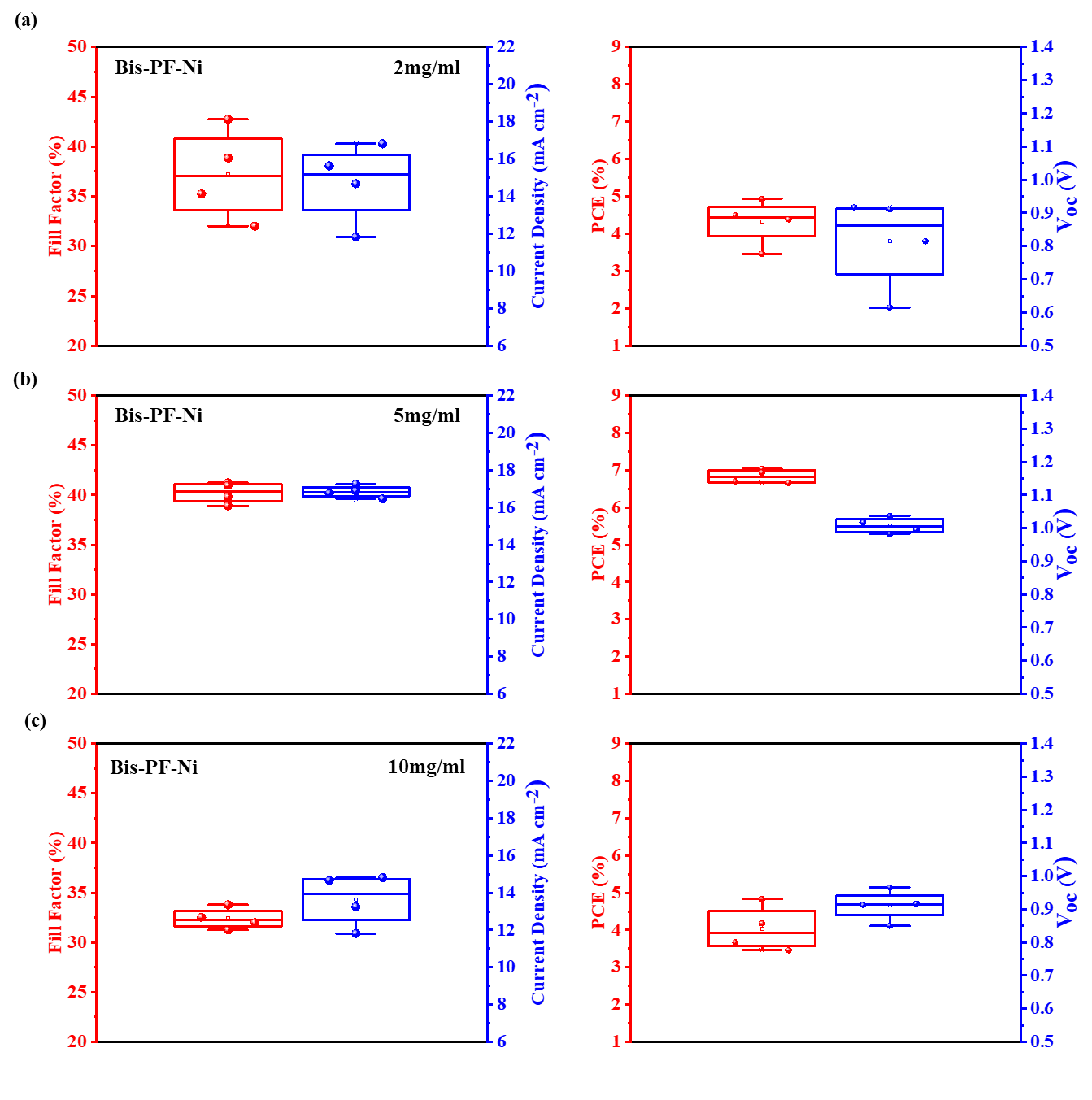


**Figure S22**. Statistics of photovoltaic parameter distributions of 4 independent devices with different concentration of Bis-PF-Ni a) 2 mg/ml b) 5 mg/ml and c) 10 mg/ml.


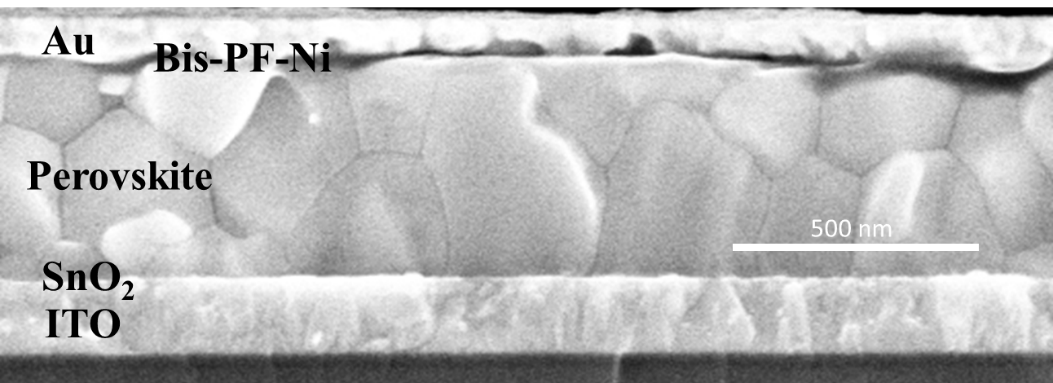


**Figure S23**. Cross sectional SEM image of the device with Bis-PF-Ni HTM.


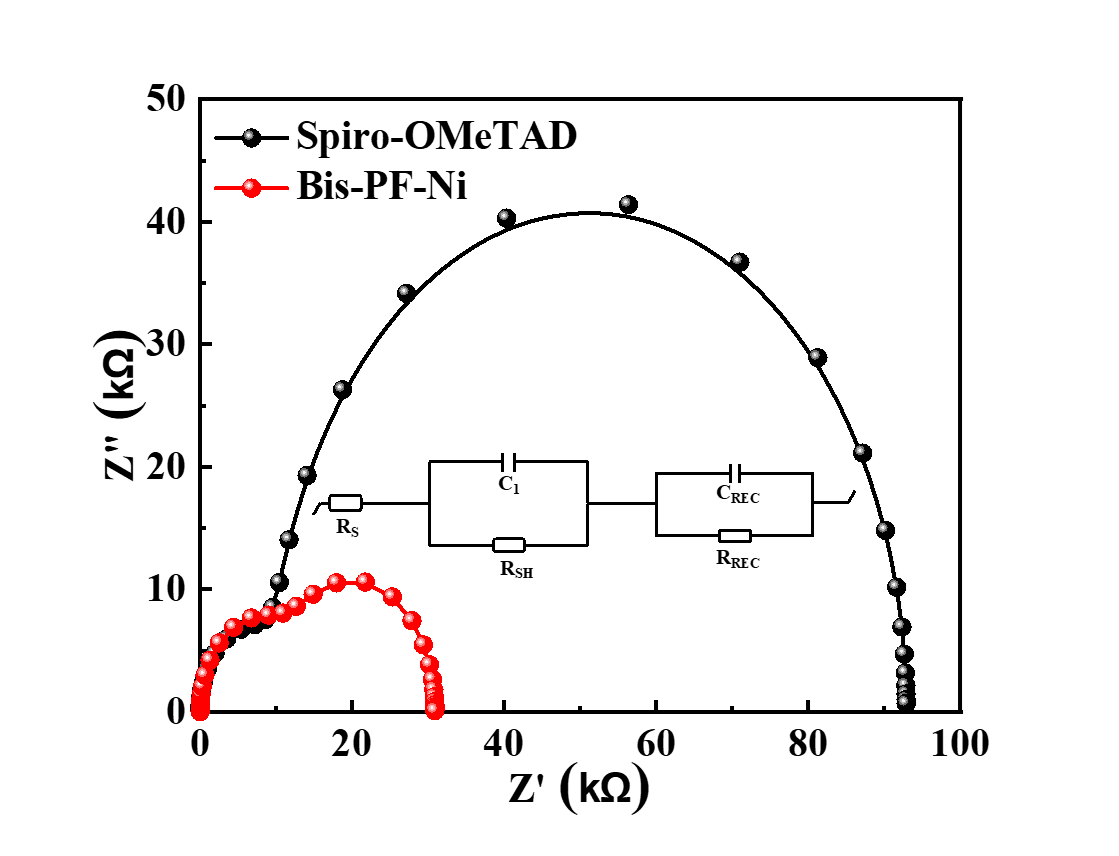


**Figure S24**. Nyquist plots of different PSCs at 0.8 V bias under dark with the fitted circuit.


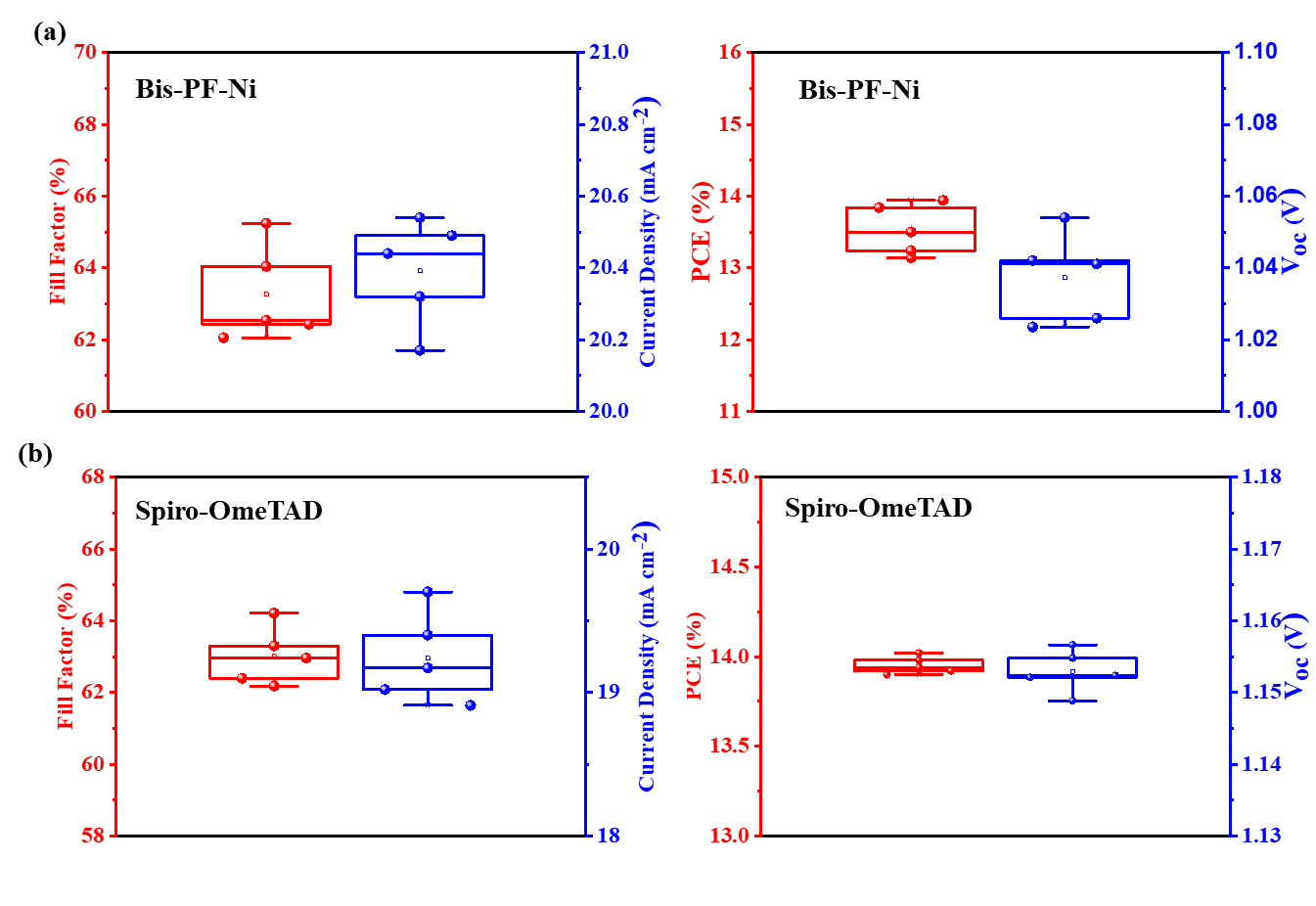


**Figure S25**. Statistics of photovoltaic parameter distributions of 5 independent devices after 21 days based on a) Bis-PF-Ni and b) spiro-oMeTAD HTLs.


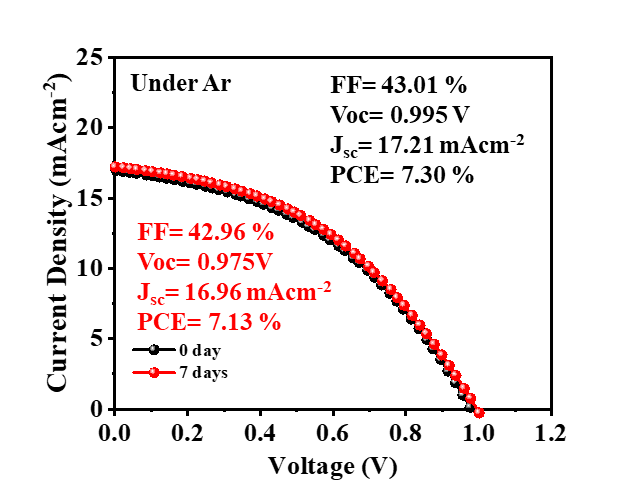


**Figure S26**. *J-V* curves of the device with Bis-PF-Ni stored under inert atmosphere.


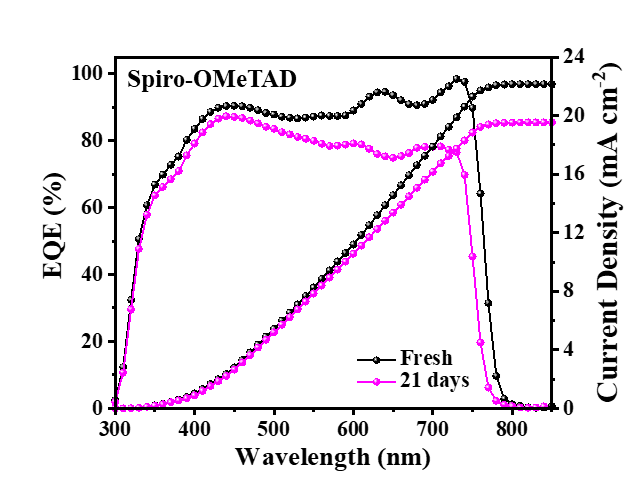


**Figure S27**. EQE and integrated *J_SC_* of the device with spiro-OMeTAD HTM (fresh and aged).


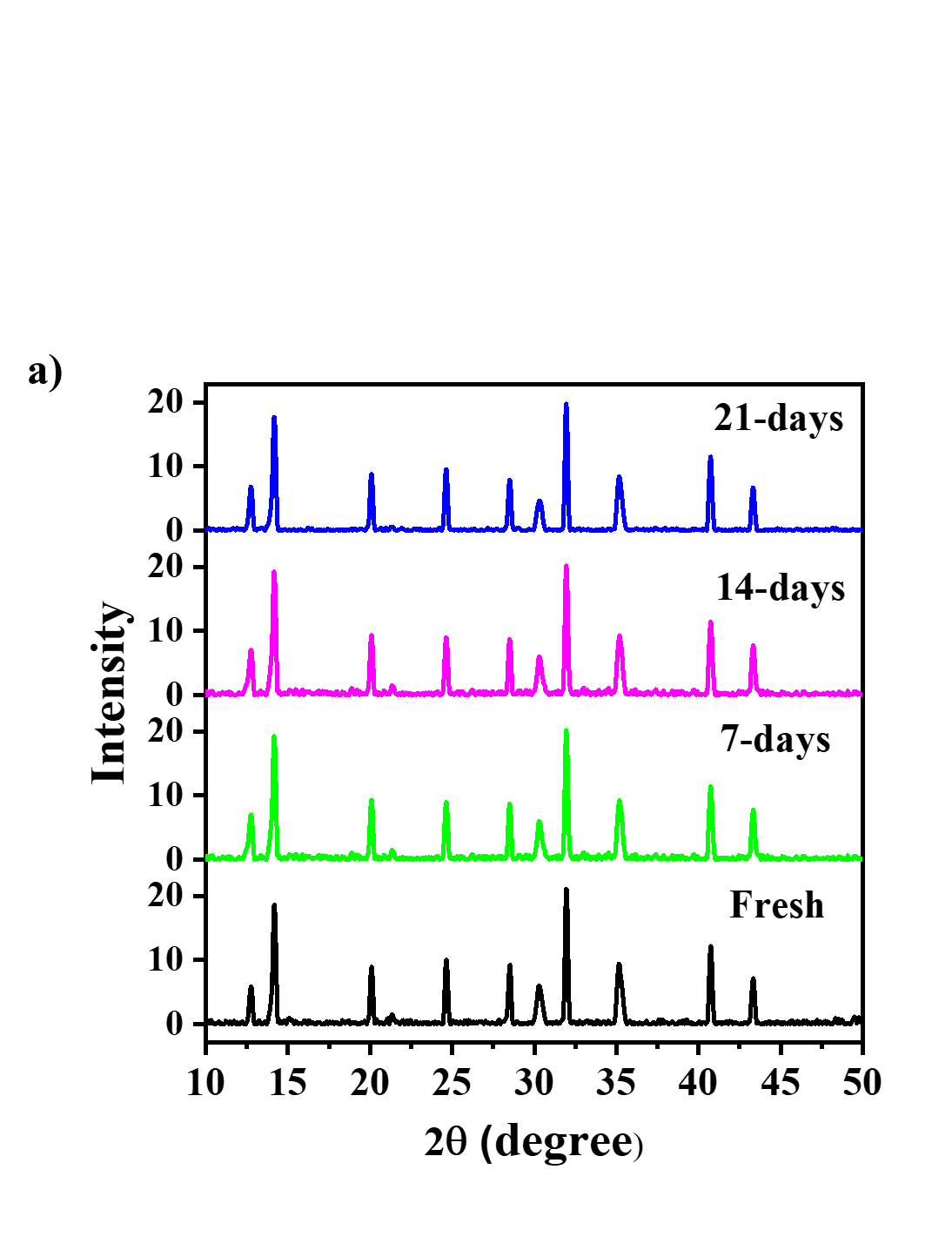


**Figure S28**. XRD patterns of the fresh and aged perovskite/Bis-PF-Ni films.


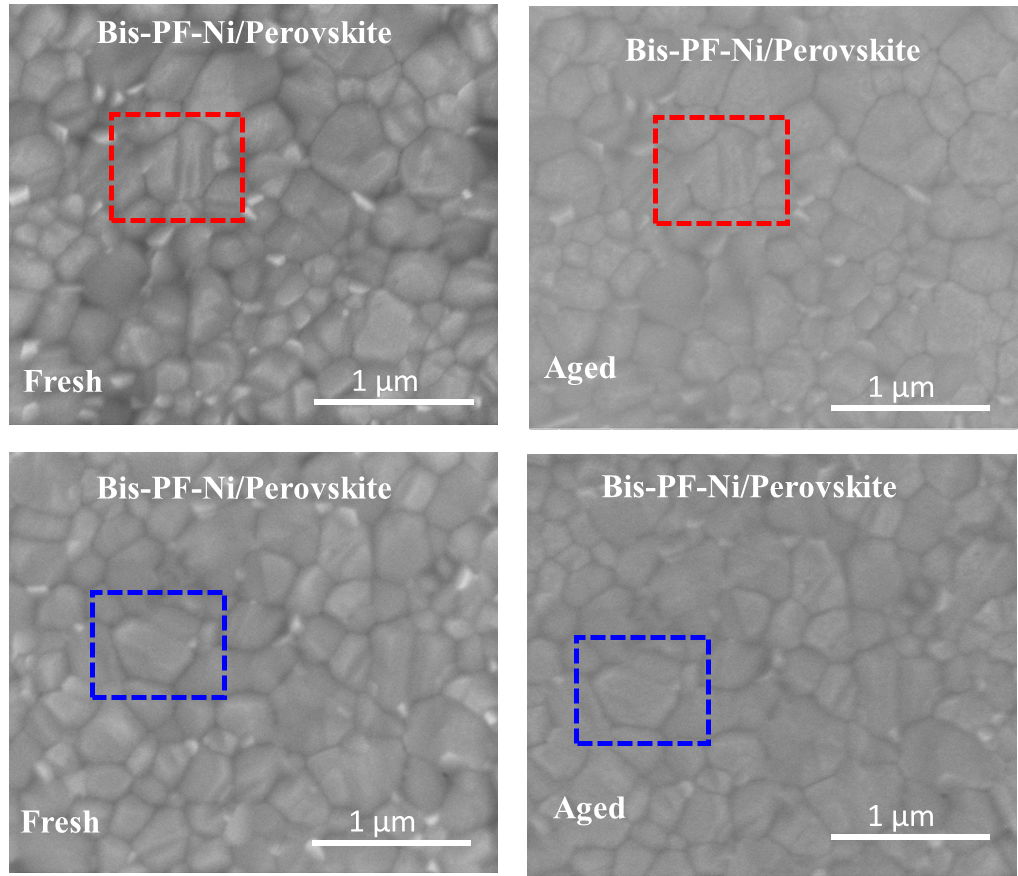


**Figure S29**. Top view SEM images of the aging study on the fresh and aged perovskite/Bis-PF-Ni films.


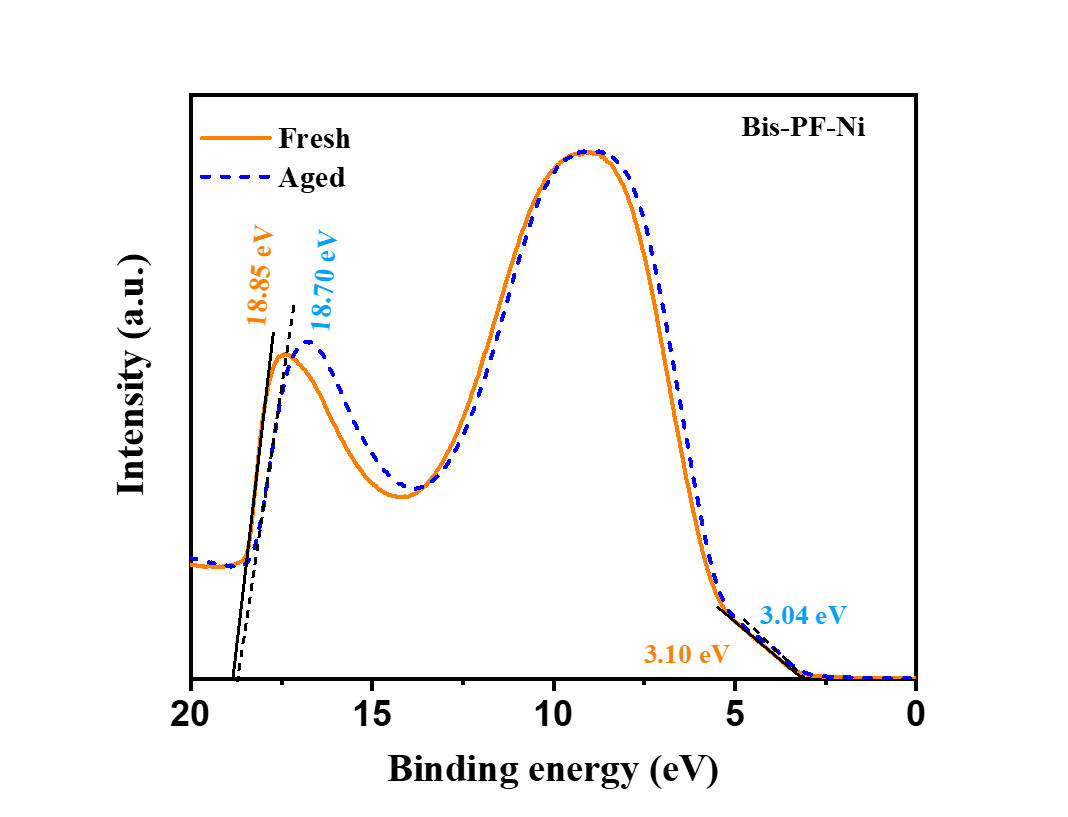


**Figure S30**. UPS spectrum of Bis-PF-Ni film. The linear interpolations reveal the photoemission cut-off energy boundary (E_cutoff_) along with the UPS photoemission onset energy (E_onset_), respectively. The work function of the film can be derived by subtracting the E_cutoff_ value from 21.22 eV. Its valence band maximum (VBM) value is estimated for fresh and aged to be -5.47 eV and -5.56 eV respectively.


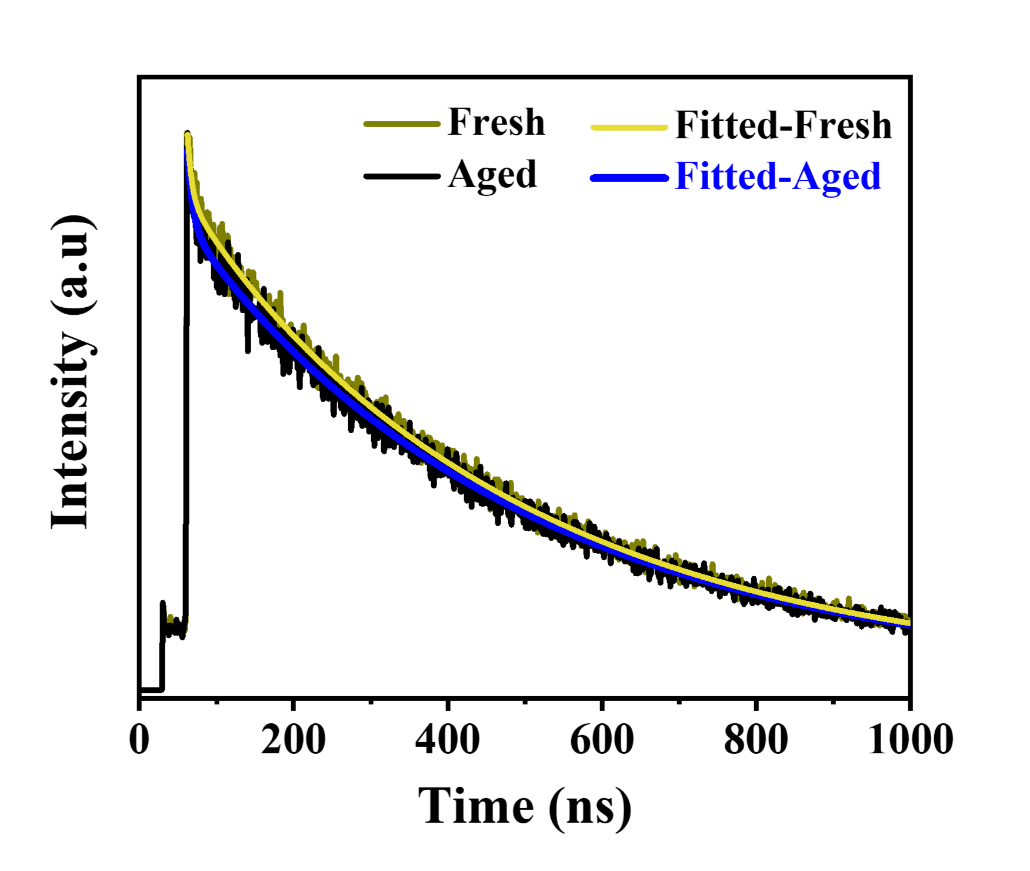


**Figure S31**. TRPL spectra of the fresh and aged perovskite/Bis-PF-Ni films.

**
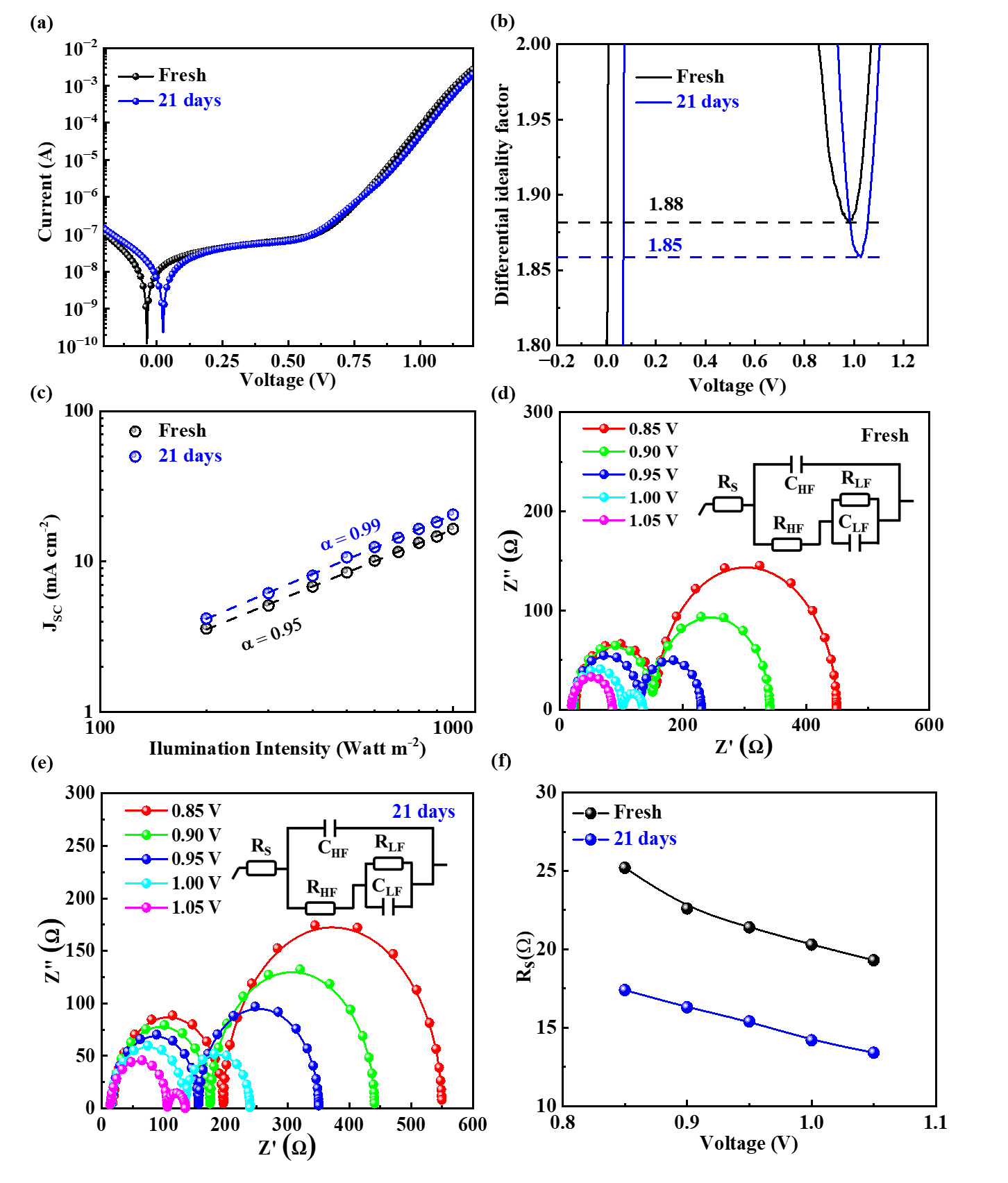
**

**Figure S32**. (a) Dark semi-log *J–V* plots; (b) differential ideality factor under dark; (c) light intensity dependant of J_SC_ of the fresh and aged Bis-PF-Ni based device; (d) Nyquist plots of the fresh and (e) aged Bis-PF-Ni based device at different bias under 1.5 G illumination; (f) series resistance of the fresh and aged Bis-PF-Ni based device extracted from Nyquist plots.


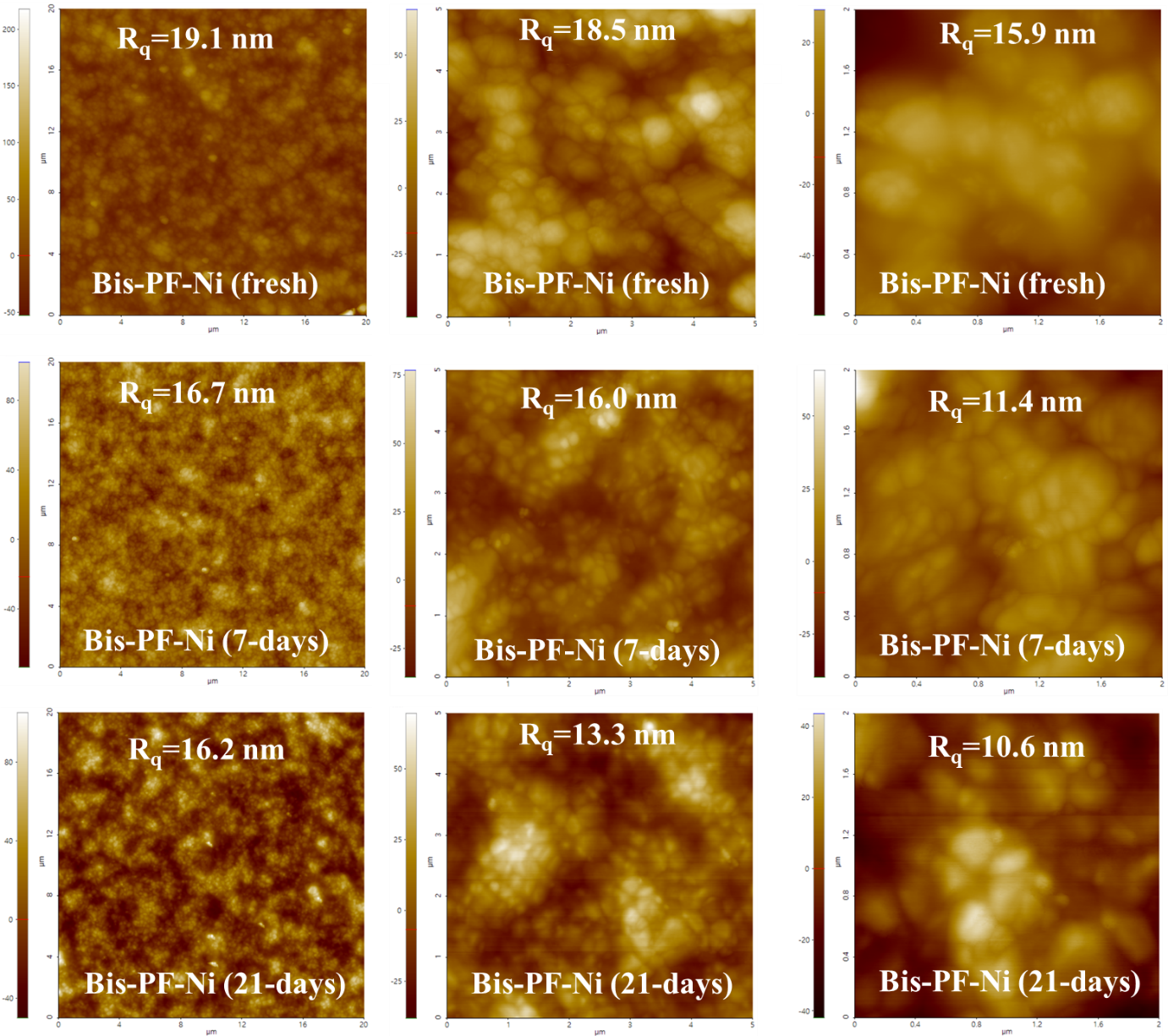


**Figure S33**. 2D AFM topography images of perovskite/Bis-PF-Ni film; fresh (top), after 7 days (middle) and 21 days (down).

**
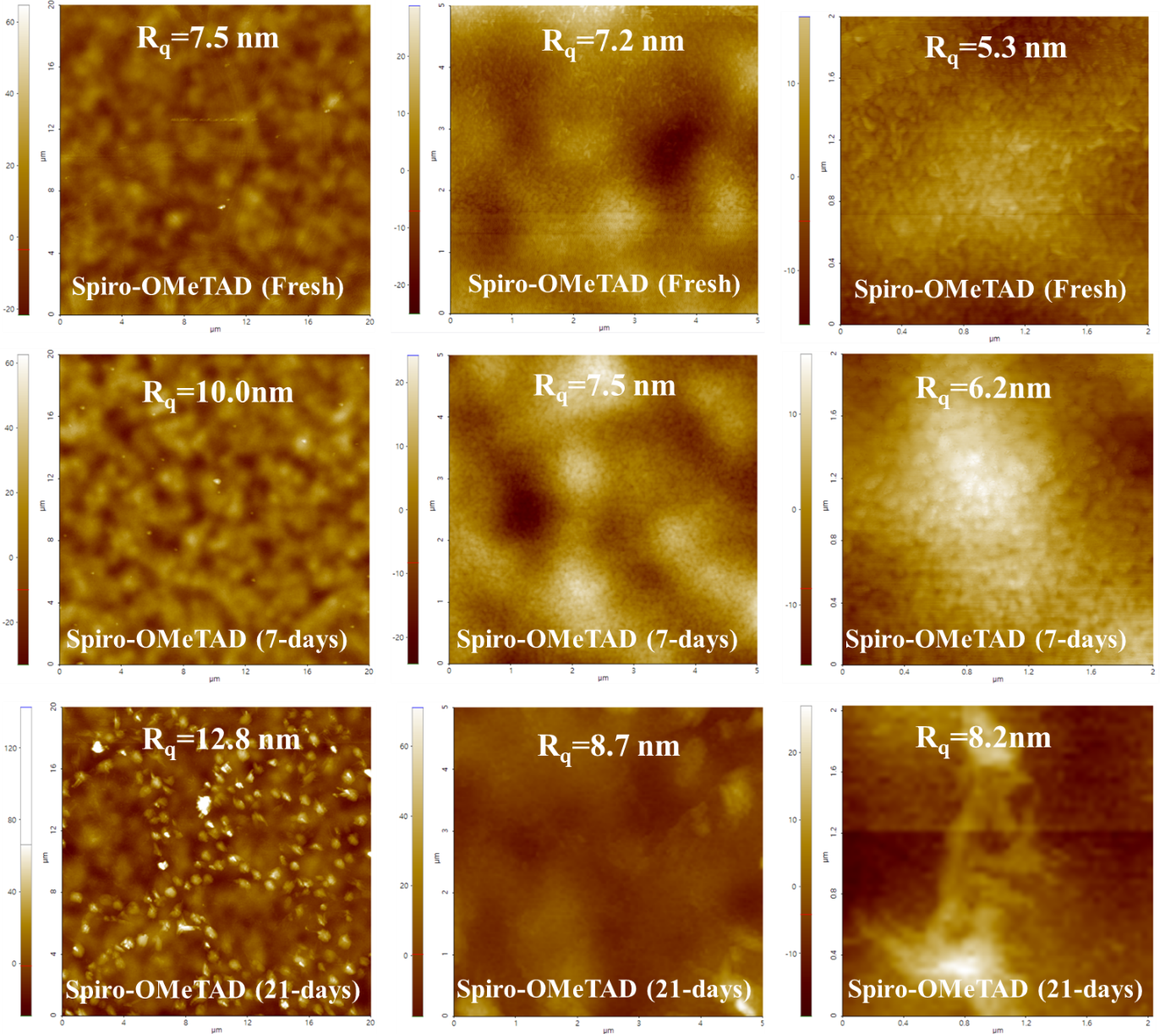
**

**Figure S34**. 2D AFM topography images of perovskite/spiro-OMeTAD/; fresh (top), after 7 days (middle) and 21 days (down).


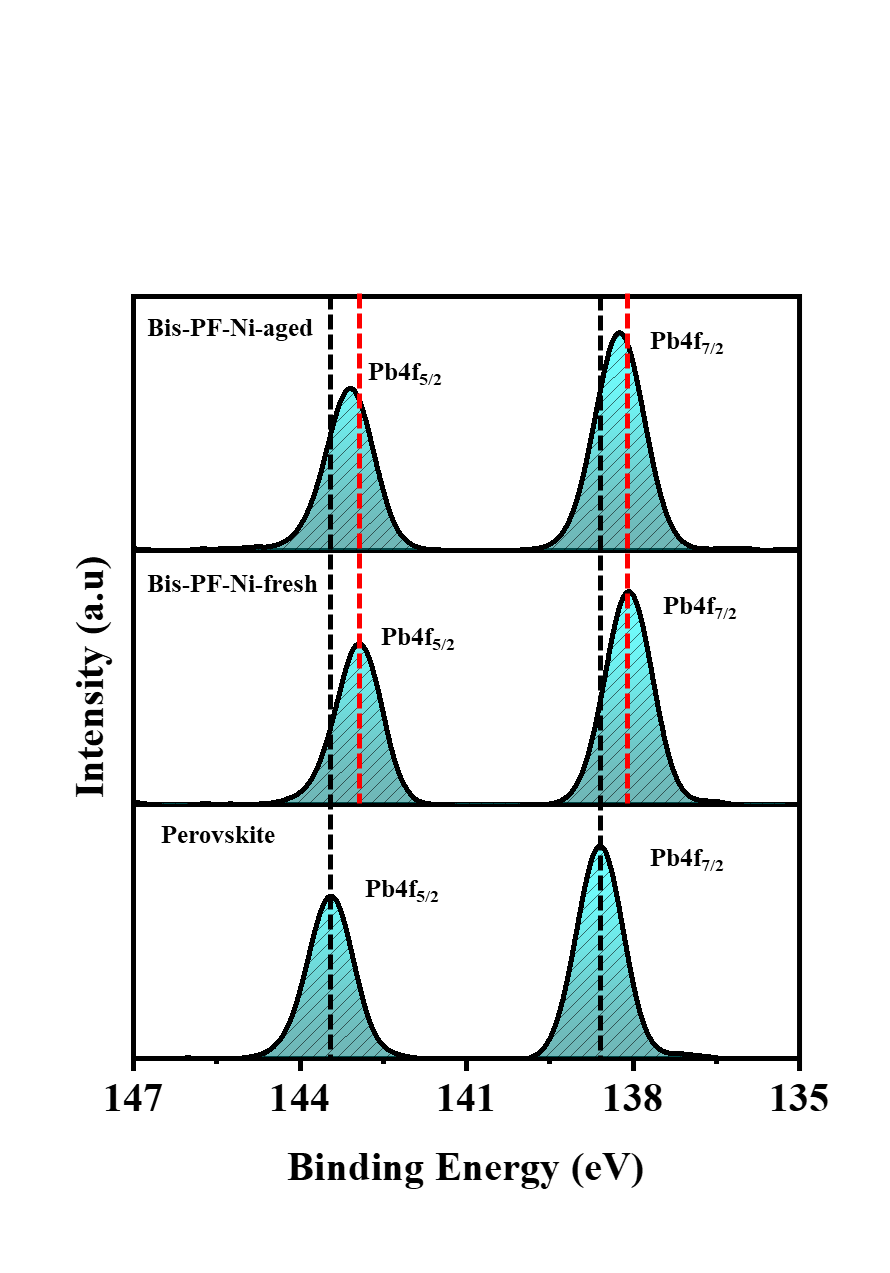


**Figure S35.** Pb 4f XPS spectrum from the pristine perovskite and perovskite/Bis-PF-Ni films (fresh and aged).


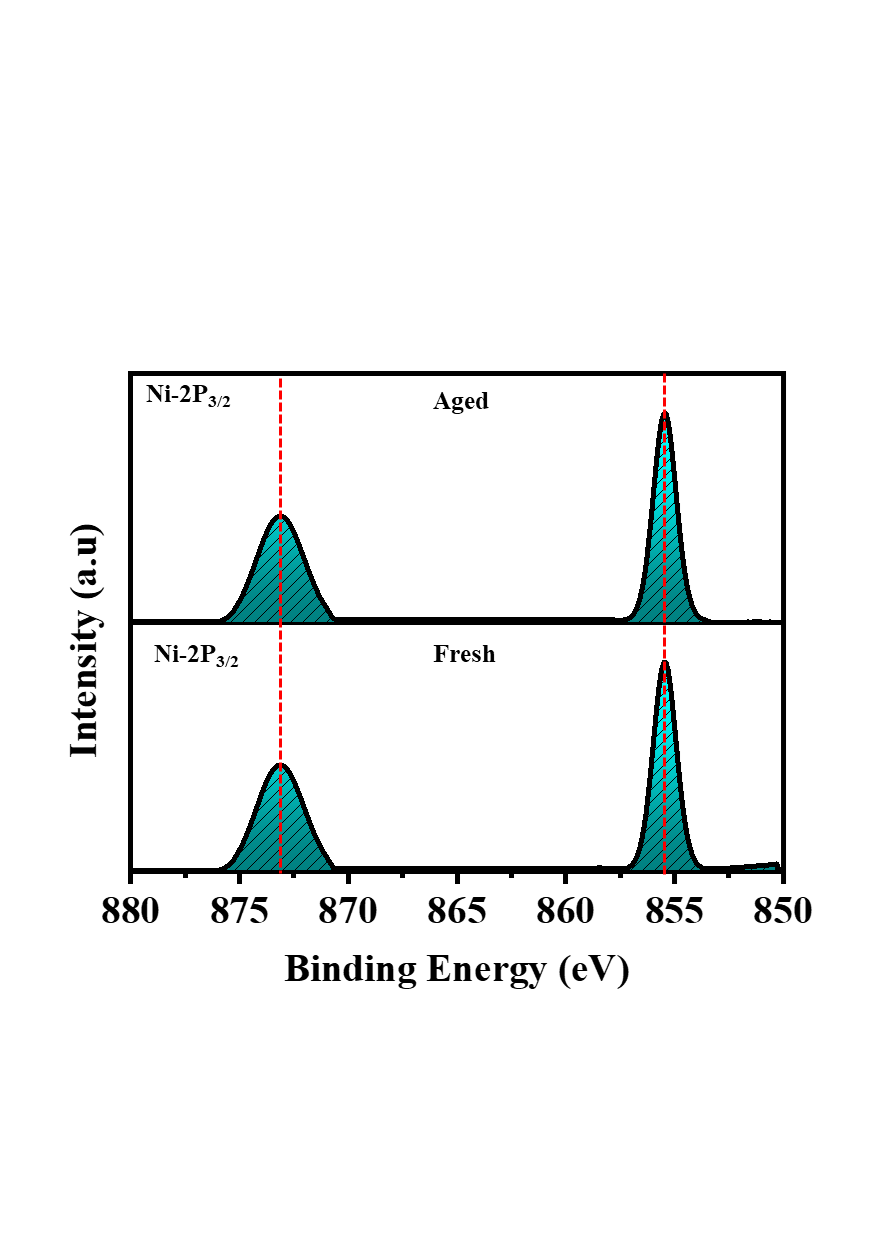


**Figure S36.** Ni 2p_3/2_ XPS spectrum from the perovskite/Bis-PF-Ni film (fresh and aged).


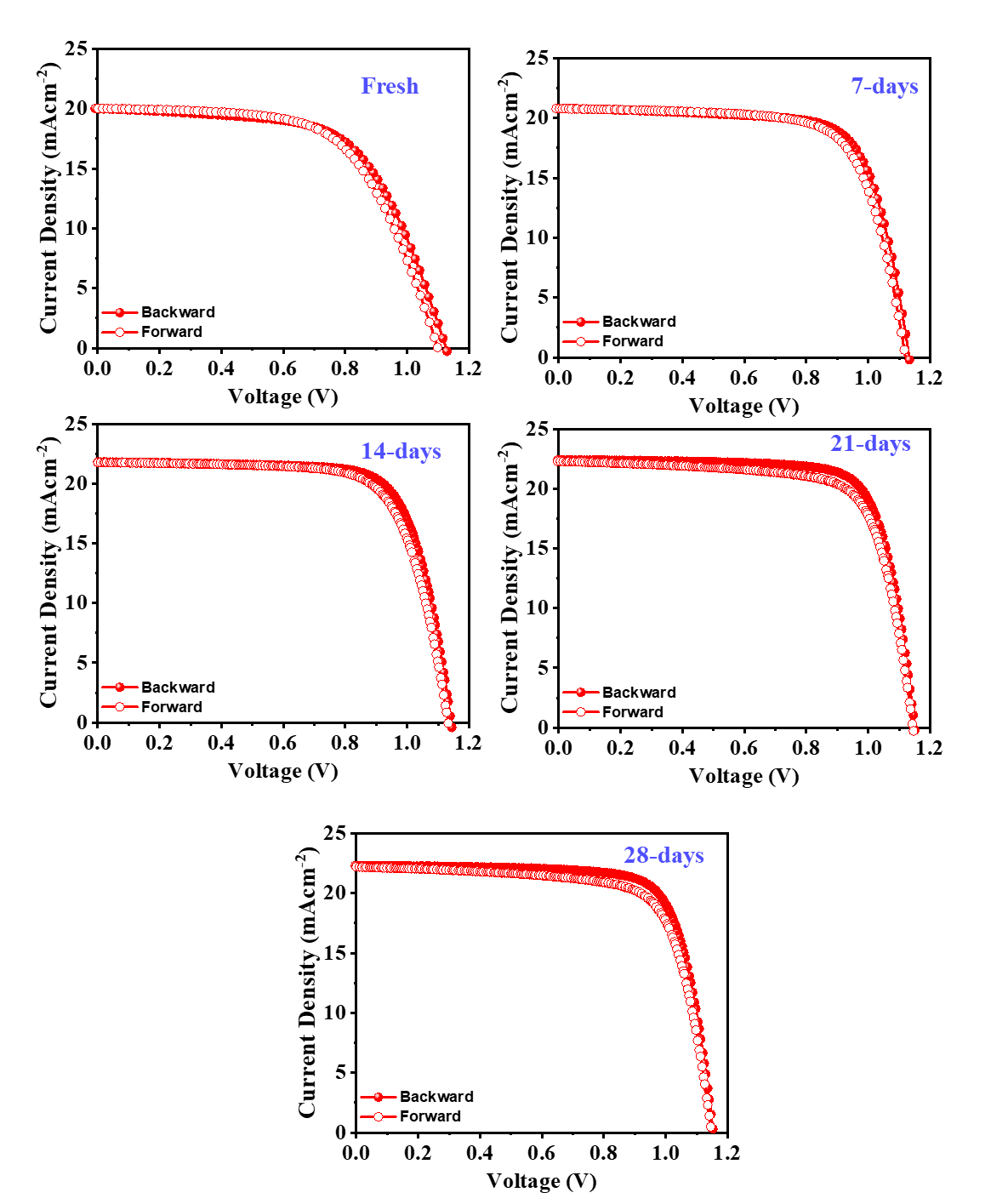


**Figure S37.** *J–V* curves of the composite HTL (fresh to aged) based devices under both forward and backward scan directions.

**
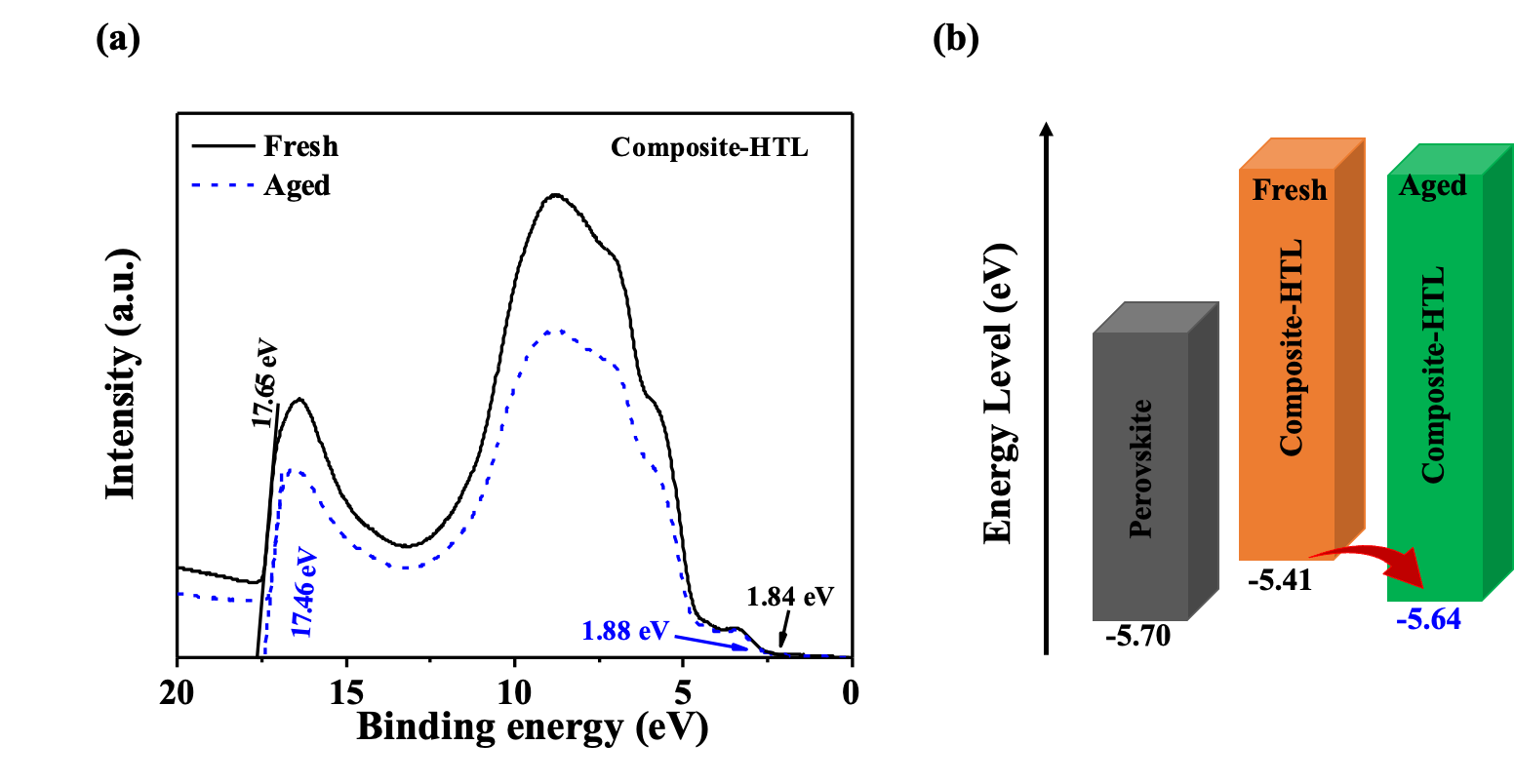
**

**Figure S38**. (a) UPS spectrum of the composite HTL film. The linear interpolations reveal the photoemission cut-off energy boundary (E_cutoff_) along with the UPS photoemission onset energy (E_onset_), respectively. The work function of the film can be derived by subtracting the E_cutoff_ value from 21.22 eV. Its valence band maximum (VBM) value is estimated for the fresh and aged films to be –5.41 eV and –5.64 eV, respectively. (b) Energy HOMO levels distribution for the perovskite and composite HTL film at different aging time.


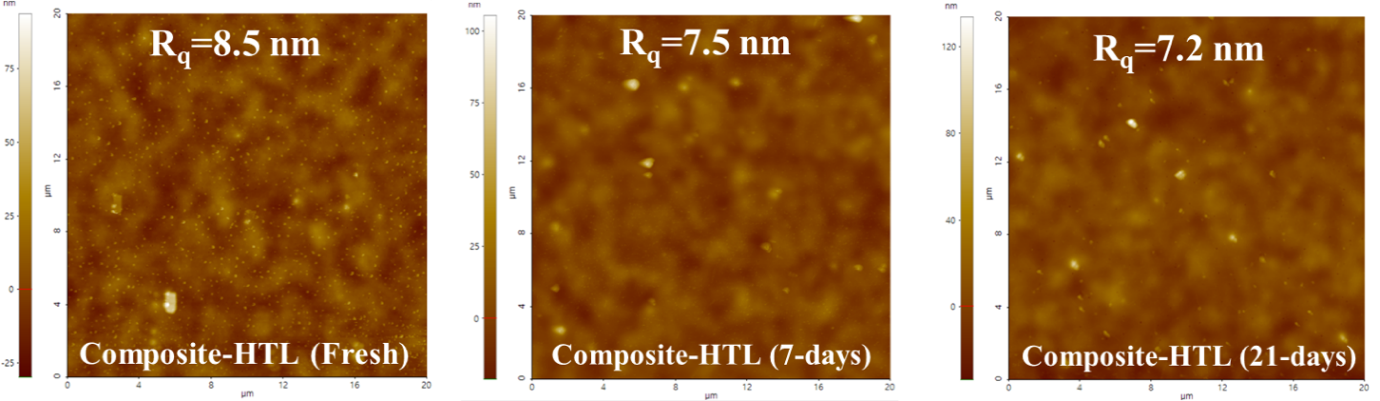


**Figure S39**. 2D AFM topography images of the perovskite/composite HTL films; fresh, after 7 days and 21 days.

**
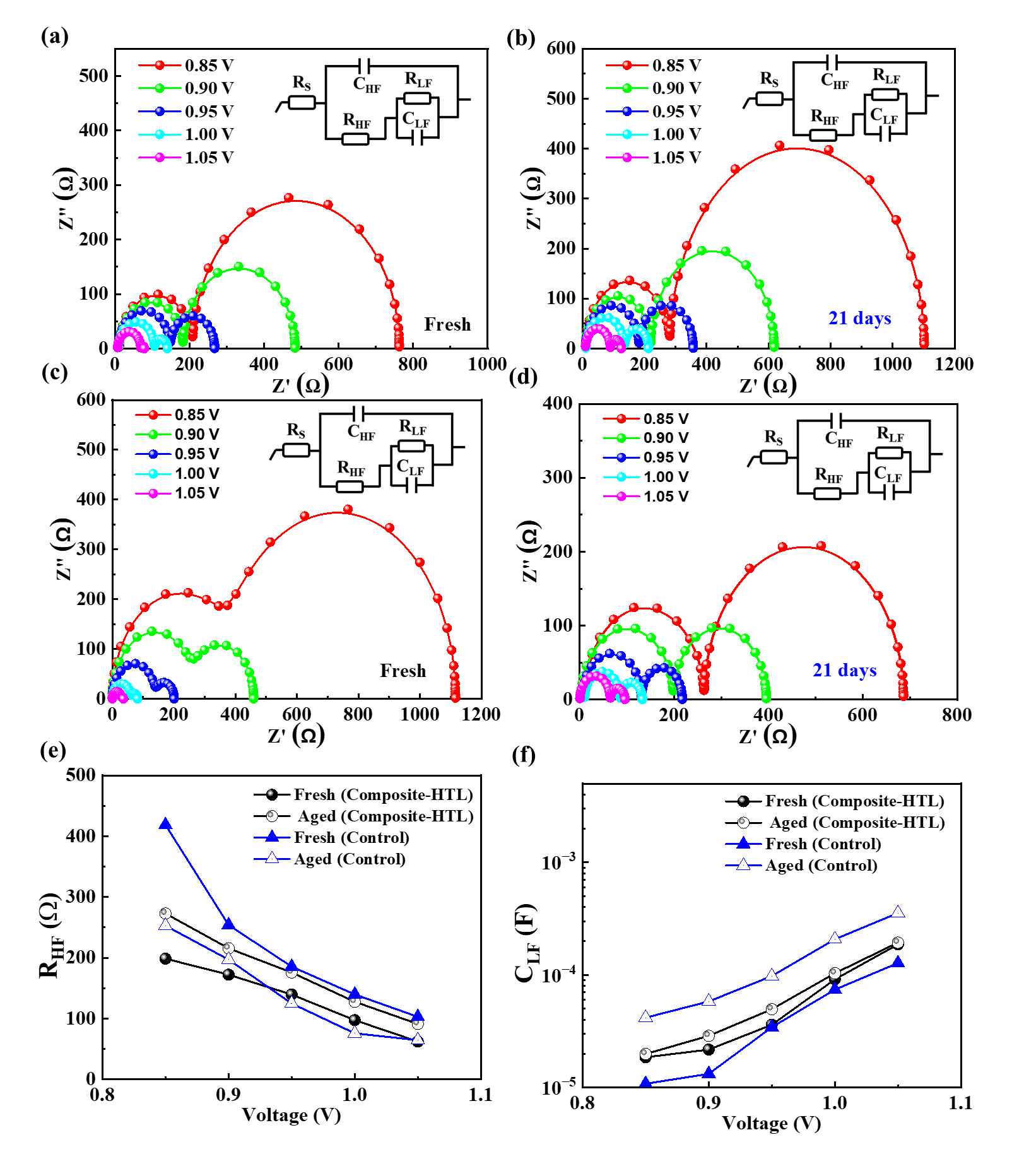
Figure S40.** Nyquist plots of the (a) fresh (1 day aged) devices with composite HTL, (b) aged devices (21 days aged) composite HTL, (c) fresh (1 day aged) devices with the control spiro-OMeTAD, and (d) aged devices (21 days aged) with the control spiro-OMeTAD at different bias under 1.5 G illumination. (e) R_HF_ and (f) C_LF_ of all the devise at different bias.


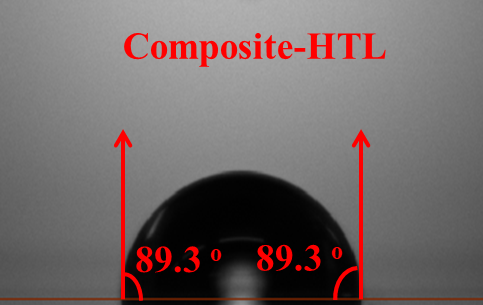


**Figure S41.** Water contact angle of the perovskite/composite HTL film.

| **Table S1.** X-ray diffraction data of liquid crystalline columnar hexagonal (Col_h_) phases of Bis-PF-Ni: 2D Lattice parameters as determined from temperature-dependent X-ray diffraction experiments. | | | | | | |
| --- | --- | --- | --- | --- | --- | --- |
| **Material** | *T*  [°C] | *2θ^o[a]^*  [°] | *d_obs_*^[b]^  [Å] | *d_calc_*^[b]^  [Å] | (*hkl*)^[c]^ | Mesophase parameters^[d]^ |
| **Bis-PF-Ni** | 30 | 3.25 | 27.20 | - | (100) | Col_h_ |
|  |  | 6.74 | 13.11 | 13.49 | (200) | a_hex_ = 31.41 Å |
|  |  | 8.79 | 10.06 | 11.68 | (210) |  |
|  |  | 9.93 | 9.00 | 9.07 | (300) |  |
|  |  | 11.20 | 7.90 | 7.86 | (310) |  |
|  |  | 19.30 | 4.60 |  |  |  |
|  |  | 26.13 | 3.41 |  |  |  |
| ^a^ 2*θ* Angle, ^b^Observed d spacing, ^c^ Calculated d spacing ^d^ Miller indices peaks | | | | | | |

| **Table S2.** Photovoltaic parameters of Bis-PF-Ni with different concentrations. | | | | | |
| --- | --- | --- | --- | --- | --- |
| **Device** | **HTLs**  **(mg/mL)** | **V_OC_**  **(V)** | **J_SC_**  **(mAcm^-2^)** | **FF**  **(%)** | **PCE**  **(%)** |
| Device 1 | 2 | 0.911 | 14.68 | 38.84 | 4.93 |
| Device 2 | 2 | 0.9168 | 11.82 | 32.01 | 3.46 |
| Device 3 | 2 | 0.615 | 16.82 | 42.75 | 4.39 |
| Device 4 | 2 | 0.815 | 15.64 | 35.25 | 4.49 |
| **Device 1** | **5** | **1.037** | **16.46** | **41.23** | **7.04** |
| Device 2 | 5 | 0.983 | 17.26 | 40.98 | 6.94 |
| Device 3 | 5 | 0.995 | 16.75 | 39.81 | 6.66 |
| Device 4 | 5 | 1.018 | 16.89 | 38.92 | 6.70 |
| Device 1 | 10 | 0.966 | 14.81 | 33.80 | 4.83 |
| Device 2 | 10 | 0.917 | 11.82 | 32.01 | 3.46 |
| Device 3 | 10 | 0.85 | 13.25 | 32.52 | 3.67 |
| Device 4 | 10 | 0.913 | 14.66 | 31.25 | 4.18 |

| **Table S3.** Photovoltaic parameters of the Bis-PF-Ni and spiro-OMeTAD based devices (forward and reverse) under different aging time. | | | | | | | | |
| --- | --- | --- | --- | --- | --- | --- | --- | --- |
| **HTL** | **Aging** | **Scan direction** | **V_OC_**  **(V)** | **J_SC_**  **(mA cm^-2^)** | **J_SC_ (EQE) (mA cm^-2^)** | **FF**  **(%)** | **PCE (%)** | **HI (%)** |
| Bis-PF-Ni | Fresh | Backward | 1.032 | 16.46 | 16.35 | 41.23 | 7.04 | 5.1 |
|  |  | Forward | 1.010 | 16.89 |  | 38.92 | 6.70 |  |
| Bis-PF-Ni | 7 days | Backward | 1.040 | 20.90 | 20.51 | 50.85 | 11.07 | 2.01 |
|  |  | Forward | 1.050 | 20.64 |  | 49.75 | 10.85 |  |
| Bis-PF-Ni | 14 days | Backward | 1.030 | 20.4 | 20.18 | 59.18 | 12.41 | 5.0 |
|  |  | Forward | 1.030 | 20.01 |  | 57.40 | 11.82 |  |
| Bis-PF-Ni | 21 days | Backward | 1.042 | 20.54 | 20.35 | 65.21 | 13.94 | 4.8 |
|  |  | Forward | 1.050 | 20.49 |  | 62.48 | 13.54 |  |
| Bis-PF-Ni | 28 days | Backward | 1.032 | 20.45 | 20.21 | 60.58 | 12.77 | 7.8 |
|  |  | Forward | 1.031 | 19.99 |  | 57.45 | 11.84 |  |
| Spiro-OMeTAD | Fresh | Backward | 1.15 | 22.45 | 22.36 | 72.37 | 18.82 | 5.4 |
|  |  | Forward | 1.139 | 22.40 |  | 69.98 | 17.85 |  |
| Spiro-OMeTAD | 7 days | Backward | 1.174 | 21.81 | 21.79 | 69.96 | 18.01 | 5.4 |
|  |  | Forward | 1.173 | 21.75 |  | 66.83 | 17.07 |  |
| Spiro-OMeTAD | 14 days | Backward | 1.164 | 21.45 | 21.24 | 64.50 | 16.14 | 5.0 |
|  |  | Forward | 1.140 | 21.42 |  | 64.02 | 15.93 |  |
| Spiro-OMeTAD | 21 days | Backward | 1.157 | 19.74 | 19.32 | 66.78 | 15.25 | 8.5 |
|  |  | Forward | 1.152 | 19.60 |  | 62.18 | 14.02 |  |
| Spiro-OMeTAD | 28 days | Backward | 1.158 | 19.31 | 18.80 | 67.73 | 15.14 | 8.7 |
|  |  | Forward | 1.155 | 19.17 |  | 62.96 | 13.94 |  |

| **Table S4.** Fitted parameters of PSCs devices from EIS. | | | |
| --- | --- | --- | --- |
| **HTM** | **R_s_^a^ (Ω)** | **R_SH_^b^ (kΩ)** | **R_REC_^c^ (kΩ)** |
| Bis-PF-Ni | 14.6 | 10.32 | 17.51 |
| Spiro-OMeTAD | 11.3 | 9.81 | 94.51 |
| ^a^ Series resistance, ^b^ Contact Resistance, ^c^ Recombination Resistance | | | |

| Table S5. Carrier lifetimes of HTMs deposited on perovskite film. | | |
| --- | --- | --- |
| Molecules | **τ_1_ (ns)** | **τ_2_ (ns)** |
| Bis-PF-Ni (Fresh) | 9.98 | 436.60 |
| Bis-PF-Ni (Aged) | 7.24 | 425.59 |

| **Table S6.** Photovoltaic parameters of the composite HTL-based devices (forward and reverse) under different aging times. | | | | | | | |
| --- | --- | --- | --- | --- | --- | --- | --- |
| **HTL** | **Aging** | **Scan direction** | **V_OC_**  **(V)** | **J_SC_**  **(mA cm^-2^)** | **FF**  **(%)** | **PCE (%)** | **HI (%)** |
| Bis-PF-Ni | Fresh | Backward | 1.125 | 19.99 | 61.25 | 13.78 | 3.2 |
|  |  | Forward | 1.099 | 20.02 | 60.71 | 13.35 |  |
| Bis-PF-Ni | 7 days | Backward | 1.132 | 20.85 | 72.55 | 17.08 | 3.5 |
|  |  | Forward | 1.116 | 20.68 | 71.76 | 16.58 |  |
| Bis-PF-Ni | 14 days | Backward | 1.144 | 22.29 | 72.98 | 18.61 | 4.4 |
|  |  | Forward | 1.142 | 21.80 | 74.89 | 18.44 |  |
| Bis-PF-Ni | 21 days | Backward | 1.150 | 22.49 | 76.38 | 19.70 | 5.6 |
|  |  | Forward | 1.144 | 22.29 | 72.98 | 18.61 |  |
| Bis-PF-Ni | 28 days | Backward | 1.153 | 22.30 | 76.18 | 19.59 | 5.7 |
|  |  | Forward | 1.147 | 22.20 | 75.55 | 18.48 |  |

| Table S7. Conductivity measurement of the control and composite HTL based films. | | | |
| --- | --- | --- | --- |
| Molecules (HTMs) | **Aging** | **Slope** | **Conductivity (S cm^-1^)** |
| Control | Fresh | 143.402 ± 0.0226 | 1.793 × 10^-5^ |
|  | Aged | 132.241 ± 0.0233 | 1.653 × 10^-5^ |
| Composite-HTL | Fresh | 125.392 ± 0.0236 | 1.567 × 10^-5^ |
|  | Aged | 153.630 ± 0.0341 | 1.921 × 10^-5^ |

**Table S8.** DFT/B3LYP/LanL2DZ optimized geometrical Cartesian coordinates of Bis-PF-Ni. The structure is at a global minimum (no imaginary frequency).

| **Symbol** | **X** | **Y** | **Z** |
| --- | --- | --- | --- |
| C | -3.14092 | -2.53932 | 0.46127 |
| C | -2.10829 | -3.44567 | 0.712727 |
| C | -0.86811 | -2.70678 | 0.586823 |
| N | -1.13674 | -1.39186 | 0.277656 |
| C | -2.50294 | -1.25846 | 0.198164 |
| C | -2.40726 | 3.22501 | -0.7223 |
| C | -3.35987 | 2.212247 | -0.54472 |
| C | -2.60786 | 0.991419 | -0.28216 |
| N | -1.25813 | 1.262255 | -0.30188 |
| C | -1.10638 | 2.602703 | -0.57015 |
| N | -3.19453 | -0.16547 | -0.05947 |
| C | 3.474175 | 2.529558 | -0.47648 |
| C | 2.438329 | 3.449469 | -0.68868 |
| C | 1.19638 | 2.711535 | -0.54966 |
| N | 1.465385 | 1.392944 | -0.26448 |
| C | 2.832787 | 1.249377 | -0.2113 |
| N | 0.016891 | 3.281619 | -0.68807 |
| C | 2.73625 | -3.21291 | 0.787104 |
| C | 3.680902 | -2.20685 | 0.573131 |
| C | 2.929689 | -0.99582 | 0.285801 |
| N | 1.581818 | -1.26154 | 0.32117 |
| C | 1.433582 | -2.59753 | 0.622508 |
| N | 0.310136 | -3.27285 | 0.750862 |
| N | 3.521075 | 0.156343 | 0.039002 |
| C | 4.80891 | 2.903636 | -0.53546 |
| C | 5.091075 | 4.242809 | -0.81836 |
| C | 4.055436 | 5.175883 | -1.03353 |
| C | 2.721467 | 4.782177 | -0.9692 |
| C | -2.80174 | 4.532647 | -0.98213 |
| C | -4.16345 | 4.80927 | -1.05764 |
| C | -5.11814 | 3.785688 | -0.87785 |
| C | -4.72164 | 2.469269 | -0.62077 |
| C | 3.141858 | -4.51587 | 1.091334 |
| C | 4.499204 | -4.77148 | 1.170723 |
| C | 5.450589 | -3.74793 | 0.95093 |
| C | 5.054138 | -2.44626 | 0.649893 |
| C | -4.48494 | -2.91914 | 0.489915 |
| C | -4.75554 | -4.25308 | 0.789839 |
| C | -3.71368 | -5.17349 | 1.050871 |
| C | -2.38807 | -4.7818 | 1.013091 |
| O | 6.41869 | 4.565297 | -0.86496 |
| O | -6.4665 | 3.996624 | -0.93354 |
| O | 6.752782 | -4.14604 | 1.05993 |
| O | -6.01095 | -4.78702 | 0.859246 |
| C | -7.13658 | -3.97171 | 0.544765 |
| C | 7.787015 | -3.19065 | 0.838014 |
| C | 9.125638 | -3.92093 | 0.981319 |
| O | 10.17765 | -2.95805 | 0.989206 |
| C | 9.379793 | -4.85742 | -0.18695 |
| O | 10.45212 | -5.71774 | 0.168414 |
| C | 10.90089 | -6.62521 | -0.7526 |
| C | 10.55884 | -2.34903 | 2.160542 |
| C | 10.351 | -6.80671 | -2.02244 |
| C | 10.89494 | -7.76933 | -2.87734 |
| C | 11.97757 | -8.56313 | -2.50079 |
| C | 12.51379 | -8.35911 | -1.22076 |
| C | 11.99044 | -7.40865 | -0.3563 |
| C | 9.966547 | -2.55548 | 3.407035 |
| C | 10.45629 | -1.87247 | 4.523441 |
| C | 11.52381 | -0.97972 | 4.433934 |
| C | 12.09512 | -0.78292 | 3.168791 |
| C | 11.62588 | -1.45212 | 2.047397 |
| C | 12.53139 | -9.62908 | -3.41964 |
| C | 11.91364 | -11.0213 | -3.18673 |
| C | 12.48172 | -12.0983 | -4.11686 |
| C | 11.87243 | -13.4869 | -3.89169 |
| C | 12.44448 | -14.558 | -4.82503 |
| C | 12.07022 | -0.27838 | 5.657206 |
| C | 13.23569 | -1.0332 | 6.325156 |
| C | 13.79173 | -0.31706 | 7.560379 |
| C | 14.95187 | -1.06245 | 8.230205 |
| C | 15.50368 | -0.34136 | 9.463539 |
| C | 6.808019 | 5.901754 | -1.16868 |
| C | -6.95135 | 5.319154 | -1.14623 |
| C | 8.339265 | 5.944874 | -1.16329 |
| O | 8.761022 | 7.304731 | -1.25155 |
| C | 8.925879 | 5.210572 | -2.35623 |
| O | 10.31235 | 5.025382 | -2.11406 |
| C | 11.07332 | 4.416721 | -3.07555 |
| C | 8.893557 | 8.069868 | -0.11784 |
| C | 10.57971 | 3.918468 | -4.28204 |
| C | 11.45598 | 3.312758 | -5.18688 |
| C | 12.81912 | 3.187019 | -4.9222 |
| C | 13.2905 | 3.699017 | -3.70429 |
| C | 12.4389 | 4.304904 | -2.79204 |
| C | 8.591076 | 7.650593 | 1.178353 |
| C | 8.773356 | 8.532029 | 2.247187 |
| C | 9.247712 | 9.830315 | 2.062016 |
| C | 9.5371 | 10.22951 | 0.749587 |
| C | 9.365262 | 9.369827 | -0.3262 |
| C | 13.74994 | 2.492444 | -5.89083 |
| C | 13.93851 | 0.99278 | -5.59143 |
| C | 14.88592 | 0.294498 | -6.57254 |
| C | 15.07877 | -1.1983 | -6.28158 |
| C | 16.02638 | -1.88984 | -7.26612 |
| C | 9.476478 | 10.7606 | 3.23206 |
| C | 10.91146 | 10.7018 | 3.790368 |
| C | 11.139 | 11.65042 | 4.971913 |
| C | 12.56399 | 11.59806 | 5.534913 |
| C | 12.78391 | 12.54876 | 6.7153 |
| C | -8.38275 | -4.84428 | 0.7302 |
| O | -9.50686 | -4.20153 | 0.132504 |
| C | -8.70391 | -5.04749 | 2.200646 |
| O | -9.64272 | -6.10748 | 2.300734 |
| C | -10.1345 | -6.43816 | 3.534631 |
| C | -9.81135 | -4.43206 | -1.1879 |
| C | -9.74404 | -5.83802 | 4.732546 |
| C | -10.3177 | -6.26235 | 5.934376 |
| C | -11.2744 | -7.27565 | 5.977881 |
| C | -11.6511 | -7.86107 | 4.760088 |
| C | -11.0957 | -7.45476 | 3.555541 |
| C | -8.99769 | -5.11562 | -2.09253 |
| C | -9.42188 | -5.2778 | -3.41376 |
| C | -10.6405 | -4.77223 | -3.86584 |
| C | -11.4359 | -4.08336 | -2.93971 |
| C | -11.0346 | -3.91254 | -1.62195 |
| C | -11.8586 | -7.75327 | 7.288489 |
| C | -11.1115 | -8.95896 | 7.89038 |
| C | -11.7113 | -9.44199 | 9.21503 |
| C | -10.9742 | -10.642 | 9.820819 |
| C | -11.5792 | -11.1193 | 11.14437 |
| C | -11.104 | -4.98784 | -5.28901 |
| C | -11.9671 | -6.25164 | -5.46766 |
| C | -12.4357 | -6.46358 | -6.91104 |
| C | -13.293 | -7.72044 | -7.09939 |
| C | -13.7576 | -7.92549 | -8.54426 |
| C | -8.47148 | 5.262484 | -1.21129 |
| O | -8.86314 | 4.730502 | -2.47854 |
| C | -9.06978 | 6.658771 | -1.06295 |
| O | -10.4745 | 6.505781 | -0.95096 |
| C | -11.261 | 7.626274 | -0.90289 |
| C | -9.70372 | 3.637873 | -2.53593 |
| C | -10.7793 | 8.935711 | -0.89814 |
| C | -11.6824 | 10.00117 | -0.84352 |
| C | -13.0606 | 9.797339 | -0.79095 |
| C | -13.5187 | 8.471397 | -0.79886 |
| C | -12.6406 | 7.398932 | -0.85431 |
| C | -9.54228 | 2.509922 | -1.7321 |
| C | -10.41 | 1.428929 | -1.88444 |
| C | -11.4318 | 1.436128 | -2.83836 |
| C | -11.5543 | 2.569816 | -3.65032 |
| C | -10.7074 | 3.663283 | -3.50291 |
| C | -14.0264 | 10.95695 | -0.692 |
| C | -14.4116 | 11.31267 | 0.756882 |
| C | -15.3955 | 12.48355 | 0.850427 |
| C | -15.7847 | 12.8444 | 2.288664 |
| C | -16.7684 | 14.01517 | 2.374189 |
| C | -12.3936 | 0.276307 | -2.96625 |
| C | -13.6559 | 0.434119 | -2.09629 |
| C | -14.6353 | -0.73648 | -2.23082 |
| C | -15.8972 | -0.58308 | -1.37384 |
| C | -16.8706 | -1.75729 | -1.51293 |
| H | 5.613474 | 2.198845 | -0.37049 |
| H | 4.289339 | 6.209569 | -1.24874 |
| H | 1.925138 | 5.497645 | -1.1334 |
| H | -2.06853 | 5.317723 | -1.12016 |
| H | -4.48128 | 5.82299 | -1.25741 |
| H | -5.46283 | 1.692332 | -0.48645 |
| H | 2.41456 | -5.30053 | 1.259108 |
| H | 4.869253 | -5.76273 | 1.403216 |
| H | 5.756978 | -1.64269 | 0.481207 |
| H | -5.26109 | -2.19563 | 0.283301 |
| H | -3.98969 | -6.19625 | 1.277266 |
| H | -1.58982 | -5.48684 | 1.209154 |
| H | -7.07839 | -3.61942 | -0.48968 |
| H | -7.18006 | -3.09781 | 1.206146 |
| H | 7.727467 | -2.38215 | 1.573068 |
| H | 7.700649 | -2.75456 | -0.16453 |
| H | 9.129254 | -4.51157 | 1.901208 |
| H | 9.635405 | -4.26712 | -1.0753 |
| H | 8.471077 | -5.43648 | -0.38607 |
| H | 9.512084 | -6.21286 | -2.36015 |
| H | 10.4589 | -7.89416 | -3.86389 |
| H | 13.36356 | -8.95261 | -0.89725 |
| H | 12.41372 | -7.25001 | 0.628442 |
| H | 9.130465 | -3.23089 | 3.531779 |
| H | 9.981133 | -2.04247 | 5.484753 |
| H | 12.92264 | -0.08882 | 3.058168 |
| H | 12.07165 | -1.29572 | 1.072361 |
| H | 13.61765 | -9.69569 | -3.2889 |
| H | 12.36428 | -9.33486 | -4.46182 |
| H | 10.8269 | -10.956 | -3.31871 |
| H | 12.07515 | -11.3146 | -2.14241 |
| H | 13.57012 | -12.1561 | -3.98361 |
| H | 12.32028 | -11.7987 | -5.16086 |
| H | 10.78531 | -13.4293 | -4.02506 |
| H | 12.03451 | -13.7867 | -2.84921 |
| H | 11.98968 | -15.535 | -4.63878 |
| H | 13.52561 | -14.6641 | -4.69002 |
| H | 12.26673 | -14.304 | -5.87489 |
| H | 11.26667 | -0.14271 | 6.389667 |
| H | 12.40881 | 0.726856 | 5.381165 |
| H | 14.03743 | -1.1758 | 5.590648 |
| H | 12.89695 | -2.03849 | 6.603111 |
| H | 12.9851 | -0.17383 | 8.291519 |
| H | 14.12565 | 0.68986 | 7.276992 |
| H | 15.75757 | -1.20539 | 7.499935 |
| H | 14.61808 | -2.0679 | 8.514007 |
| H | 16.32858 | -0.89839 | 9.916526 |
| H | 14.72977 | -0.21485 | 10.22735 |
| H | 15.87797 | 0.654406 | 9.205295 |
| H | 6.420567 | 6.597801 | -0.41843 |
| H | 6.42689 | 6.202651 | -2.15214 |
| H | -6.56634 | 5.720999 | -2.08985 |
| H | -6.63023 | 5.967135 | -0.32153 |
| H | 8.719679 | 5.476158 | -0.25197 |
| H | 8.767635 | 5.806717 | -3.26334 |
| H | 8.420692 | 4.244407 | -2.46527 |
| H | 9.529979 | 3.994665 | -4.53265 |
| H | 11.05686 | 2.934645 | -6.12319 |
| H | 14.34801 | 3.627162 | -3.46872 |
| H | 12.8098 | 4.70499 | -1.85586 |
| H | 8.213458 | 6.656544 | 1.378446 |
| H | 8.529395 | 8.190068 | 3.24834 |
| H | 9.90242 | 11.2352 | 0.565278 |
| H | 9.592476 | 9.683718 | -1.33802 |
| H | 14.72922 | 2.984572 | -5.87307 |
| H | 13.36635 | 2.606912 | -6.91081 |
| H | 12.95931 | 0.499347 | -5.6101 |
| H | 14.31695 | 0.876473 | -4.56874 |
| H | 15.86304 | 0.795082 | -6.5526 |
| H | 14.50448 | 0.416321 | -7.59501 |
| H | 14.10293 | -1.69846 | -6.30166 |
| H | 15.46053 | -1.32019 | -5.26071 |
| H | 16.14178 | -2.95153 | -7.03077 |
| H | 17.02174 | -1.43475 | -7.24344 |
| H | 15.65342 | -1.81597 | -8.2927 |
| H | 8.771477 | 10.51728 | 4.034787 |
| H | 9.25462 | 11.79018 | 2.928664 |
| H | 11.61859 | 10.93892 | 2.986401 |
| H | 11.13371 | 9.672278 | 4.095792 |
| H | 10.42617 | 11.4115 | 5.772221 |
| H | 10.91161 | 12.67875 | 4.661225 |
| H | 13.27612 | 11.83725 | 4.735826 |
| H | 12.79096 | 10.57112 | 5.845953 |
| H | 13.80841 | 12.48701 | 7.092759 |
| H | 12.10985 | 12.31233 | 7.544802 |
| H | 12.59893 | 13.58822 | 6.426096 |
| H | -8.21174 | -5.82393 | 0.276308 |
| H | -9.12173 | -4.1203 | 2.611689 |
| H | -7.78145 | -5.29737 | 2.737506 |
| H | -9.0066 | -5.04654 | 4.748925 |
| H | -10.007 | -5.78139 | 6.856939 |
| H | -12.4004 | -8.64684 | 4.754852 |
| H | -11.3963 | -7.9052 | 2.617016 |
| H | -8.03811 | -5.51924 | -1.79736 |
| H | -8.77357 | -5.80725 | -4.10521 |
| H | -12.389 | -3.67149 | -3.25667 |
| H | -11.6563 | -3.38445 | -0.90877 |
| H | -12.9107 | -8.02389 | 7.142254 |
| H | -11.8501 | -6.93098 | 8.012614 |
| H | -10.0591 | -8.68868 | 8.03888 |
| H | -11.114 | -9.78072 | 7.164167 |
| H | -12.7658 | -9.70671 | 9.061158 |
| H | -11.7093 | -8.61534 | 9.937772 |
| H | -9.92105 | -10.3775 | 9.97512 |
| H | -10.9768 | -11.468 | 9.099299 |
| H | -11.0312 | -11.9749 | 11.54893 |
| H | -12.6226 | -11.4243 | 11.01535 |
| H | -11.5585 | -10.3256 | 11.89799 |
| H | -10.2325 | -5.05495 | -5.9498 |
| H | -11.6768 | -4.11507 | -5.62247 |
| H | -12.8376 | -6.18809 | -4.80355 |
| H | -11.3943 | -7.12523 | -5.13397 |
| H | -11.5608 | -6.52145 | -7.57205 |
| H | -13.0061 | -5.58512 | -7.24053 |
| H | -14.1671 | -7.66263 | -6.43949 |
| H | -12.7228 | -8.59799 | -6.77118 |
| H | -14.3655 | -8.82905 | -8.64429 |
| H | -12.9051 | -8.02207 | -9.22416 |
| H | -14.3607 | -7.07983 | -8.88982 |
| H | -8.84762 | 4.632711 | -0.40097 |
| H | -8.81648 | 7.263413 | -1.94244 |
| H | -8.66828 | 7.148443 | -0.16586 |
| H | -9.71825 | 9.143723 | -0.93957 |
| H | -11.2922 | 11.0144 | -0.84656 |
| H | -14.5862 | 8.275426 | -0.7682 |
| H | -12.9994 | 6.376708 | -0.86834 |
| H | -8.73133 | 2.463273 | -1.01584 |
| H | -10.273 | 0.553426 | -1.2571 |
| H | -12.33 | 2.602103 | -4.40944 |
| H | -10.8116 | 4.543104 | -4.1263 |
| H | -14.9373 | 10.72241 | -1.25466 |
| H | -13.5872 | 11.83906 | -1.1711 |
| H | -13.501 | 11.55011 | 1.320201 |
| H | -14.845 | 10.42854 | 1.239715 |
| H | -16.3027 | 12.24184 | 0.281135 |
| H | -14.9585 | 13.36522 | 0.363153 |
| H | -14.8786 | 13.08653 | 2.857252 |
| H | -16.2218 | 11.96403 | 2.775272 |
| H | -17.0252 | 14.24653 | 3.411694 |
| H | -17.6993 | 13.78942 | 1.844318 |
| H | -16.3458 | 14.92063 | 1.927111 |
| H | -11.8831 | -0.6534 | -2.69274 |
| H | -12.6956 | 0.166903 | -4.01417 |
| H | -14.1619 | 1.368885 | -2.36638 |
| H | -13.3554 | 0.544113 | -1.04737 |
| H | -14.1261 | -1.66989 | -1.95671 |
| H | -14.9263 | -0.84761 | -3.28388 |
| H | -16.4071 | 0.348514 | -1.64784 |
| H | -15.607 | -0.47201 | -0.32206 |
| H | -17.7586 | -1.61632 | -0.89044 |
| H | -16.4007 | -2.69887 | -1.21156 |
| H | -17.2063 | -1.87251 | -2.54853 |
| Ni | 0.162841 | 0.000017 | 0.007411 |
